# A Brønsted base-promoted diastereoselective dimerization of azlactones

**DOI:** 10.3762/bjoc.13.264

**Published:** 2017-12-13

**Authors:** Danielle L J Pinheiro, Gabriel M F Batista, Pedro P de Castro, Leonã S Flores, Gustavo F S Andrade, Giovanni W Amarante

**Affiliations:** 1 Chemistry Department, Federal University of Juiz de Fora, Cidade Universitária, São Pedro, Juiz de Fora, MG, CEP 36036-900, Brazil

**Keywords:** azlactones, dimerization, diasteoreselective synthesis, kinetics, streptopyrrolidine analogue

## Abstract

A novel Brønsted base system for the diastereoselective dimerization of azlactones using trichloroacetate salts and acetonitrile has been developed. Desired products were obtained in good yields (60–93%) and with up to >19:1 dr after one hour of reaction. Additionally, the relative stereochemistry of the major dimer was assigned as being *trans*, by X-ray crystallographic analysis. The kinetic reaction profile was determined by using ^1^H NMR reaction monitoring and revealed a second order overall kinetic profile. Furthermore, by employing this methodology, a diastereoselective total synthesis of a functionalized analogue of streptopyrrolidine was accomplished in 65% overall yield.

## Findings

Azlactones have been acknowledged as a common nucleophilic reagent to introduce a quaternary carbon stereocenter in the α-position of α-amino acid derivatives. To this end, different catalytic transformations involving azlactones have been established [1–19]. For example, our research group has explored the potential of these heterocycles in the presence of Michael and Mannich acceptors [20–23].

**Figure 1:**
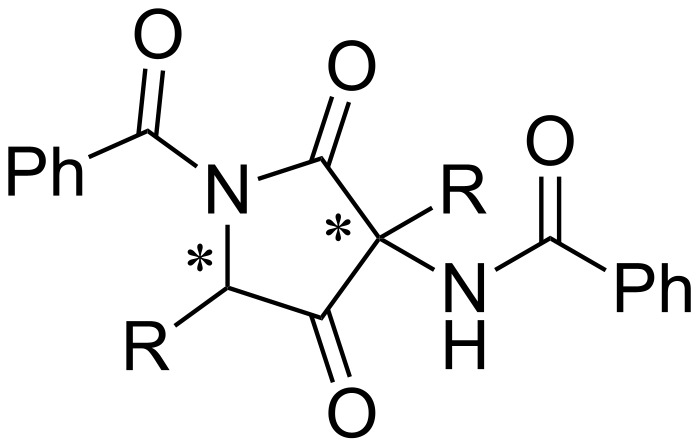
Structure of an azlactone dimer.

Enolate anions derived from azlactone are effective intermediates for the functionalization of α-amino acids. However, the acylation of the enolate ion by another azlactone has been less reported in the literature [24–26]. This transformation affords an azlactone dimer, which is a versatile heterocycle containing two stereogenic centers (Figure 1).

Kobayashi and co-workers demonstrated the rich reactivity of azlactones conducting the dimerization reaction using strong bases as catalysts; however, higher temperatures and long reaction times were required [27]. Moreover, no evidence regarding either the diastereoselectivity and stereochemistry were reported. In 1998, Mazurkiewicz and co-workers developed a kinetic study of the base-catalyzed dimerization of azlactones. In this investigation, it was established that the dimerization reaction rates are strongly dependent on the base, as well as the substituent at the C2 position [24]. Once again, no comments concerning the stereochemistry were addressed.

Thus, we started this study envisioning the trichloromethylation of azlactones through the decarboxylative potassium trichloroacetate (KTCA) in DMSO. Although no trichloromethylation product was observed, we found out that by switching the solvent, the isolated major product comes from dimerization of azlactones. At this point, we turned our attention towards this product, envisioning the development of an atom-economic reaction for stereoselective C–C bond formation, such as a dimerization process.

We herein report a diastereoselective dimerization of azlactones using potassium or sodium trichloroacetate salts and acetonitrile as solvent. Besides, a reaction mechanism is proposed based on NMR studies and the relative stereochemistry of the major diastereomer was assigned by X-ray analysis. Finally, one of the products was selectively reduced to provide a functionalized analogue of streptopyrrolidine, a marine natural product isolated from *Streptomyces* sp.

As shown in Table 1, our studies started with the synthesis of dimer **2a** using azlactone **1a** in the presence of potassium trichlororacetate salt (2 equiv), DMSO as solvent, at room temperature. As preliminary results, we observed the formation of several undesired products, detected by ^1^H NMR analyses of the crude reaction mixtures. Several salts were evaluated, with great influence in reaction yields and dr; it is also worth mentioning that when employing KTFA, NaTFA and NaHCO_3_ no product was observed (Table 1, entries 16, 17 and 18). Besides, different solvents were also evaluated and the most promising result was found using acetonitrile (Table 1, entry 6). This probably was due to the formation of an intermediate between acetonitrile and the trichloroacetate salt (see mechanism discussion below). Under these reaction conditions the desired product was isolated in excellent conversion and with moderate dr 2:1. Encouraged by this result, we have then evaluated the effect of the salt concentration on the reaction outcome. Decreasing the amount of KTCA to 0.3 equiv, the diastereoselectivity slightly increased to 4:1 (Table 1, entry 9). Finally, NaTCA as salt gave 98% conversion and a good dr (6:1, Table 1, entry 13). Different basic conditions were evaluated without any improvements in terms of yields and selectivities.

**Table 1:** Optimization reaction conditions of azlactones dimerization.

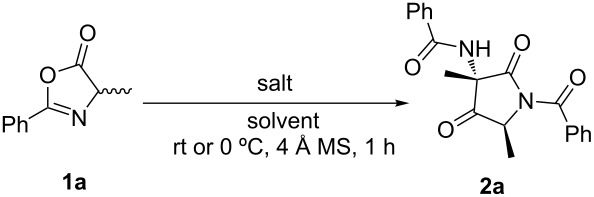						

Entry	Salt/base	Equiv	Solvent	Temp. (°C)	Conv.^a^ ( %)	dr^a^

**1**	KTCA^b^	2	DMSO	rt	mix of products	–
**2**	KTCA	1	DMSO	rt	mix of products	–
**3**	KTCA	2	DMF	rt	mix of products	–
**4**	KTCA	2	DMF	0	–	–
**5**	KTCA	2	CH_2_Cl_2_	rt	–	–
**6**	KTCA	2	CH_3_CN	rt	95	2:1
**7**	KTCA	1	CH_3_CN	rt	95	2:1
**8**	KTCA	0.7	CH_3_CN	rt	95	3:1
**9**	KTCA	0.3	CH_3_CN	rt	94	4:1
**10**	KTCA	0.3	CH_3_CN	0	–	–
**11**	KTCA	0.2	CH_3_CN	rt	82	4:1
**12**	NaTCA^c^	0.2	CH_3_CN	rt	94	6:1
**13**	NaTCA	0.3	CH_3_CN	rt	98	6:1
**14**	NaTCA	0.3	CH_3_CN	0	–	–
**15**	LiTCA^d^	0.3	CH_3_CN	rt	88	3:1
**16**	KTFA^e^	0.3	CH_3_CN	rt	–	–
**17**	NaTFA^f^	0.3	CH_3_CN	rt	–	–
**18**	NaHCO_3_	0.3	CH_3_CN	rt	–	–
**19**	Et_3_N	0.3	CH_3_CN	rt	34	1:1
**20**	NaOH	0.3	CH_3_CN	rt	10	1:1
**21**	NaOH	0.5	CH_3_CN	rt	98	2:1

^a^Measured by ¹H NMR analysis of the crude reaction mixture. ^b^ Potassium trichloroacetate. ^c^Sodium trichloroacetate. ^d^Lithium trichloroacetate. ^e^Potassium trifluoroacetate. ^f^Sodium trifluoroacetate.

**Scheme 1:**
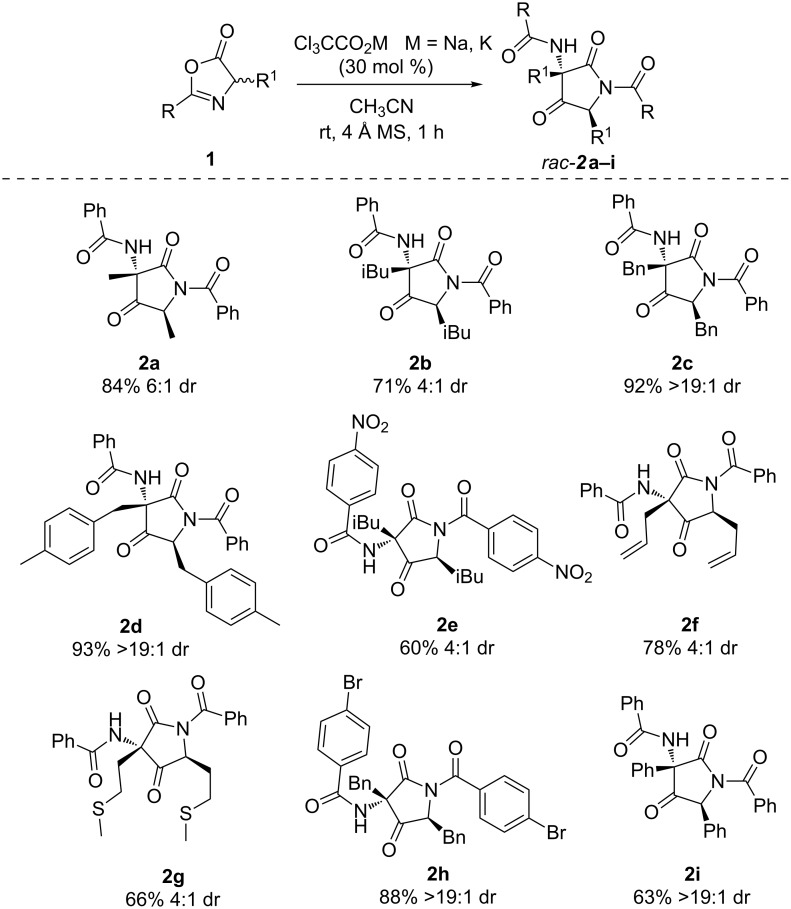
Diastereoselective dimerization of azlactones. Reactions were carried out using 0.45 mmol of **1** and 0.14 mmol of salt. Diastereomer ratio measured by ^1^H NMR analysis of the crude reaction mixture.

With the optimal reaction conditions established, the scope of the reaction was then investigated (Scheme 1). The results revealed that the dimerization reaction shows tolerance to changes of functional groups at both the substituents R and R^1^. For example, in the presence of an electron-withdrawing aryl ring substituent R, the reaction worked well and afforded the product **2e** in good yield and diastereoselectivity. When R^1^ was a benzyl group, the reaction tolerated the use of both electron-donating substituents and a non-substituted phenyl ring, affording the desired products in excellent yields and with up to dr >19:1 (products **2c**, **2d** and **2h**). Substrates bearing an alkyl chain as an R^1^ group were well tolerated (**2a** and **2b**). Importantly, azlactones containing a thioether or allyl substituents could be also accessed in good isolated yields (**2f** and **2g**).

**Figure 2:**
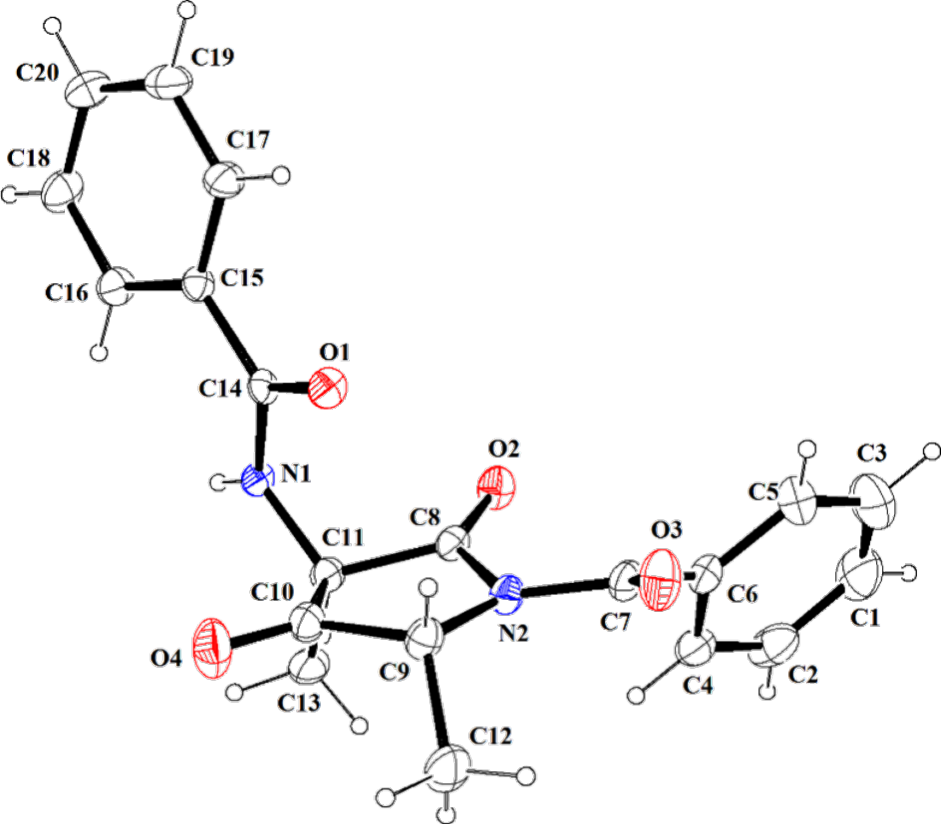
X-ray crystallographic structure of **2a** (30% ellipsoids probability).

The major diastereomer was isolated after purification through column chromatography or simple recrystallization in good to excellent yields. The relative configuration to C11 and C9 of the major diastereomer was assigned by X-ray crystallographic analysis of compound **2a** (Figure 2) [28]. The other products were assigned by analogy.

**Scheme 2:**
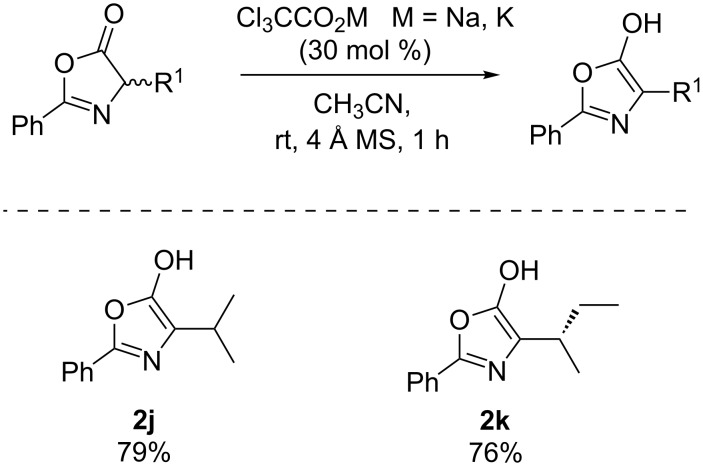
Sterically bulky azlactone enol derivatives.

An interesting observation was found when employing azlactones derived from more sterically bulky amino acids, such as valine or isoleucine. In this case, the dimerization process does not occur. We isolate the corresponding azlactone enol derivatives **2j** and **2k**, which are very stable and unreactive species (Scheme 2) [29].

**Figure 3:**
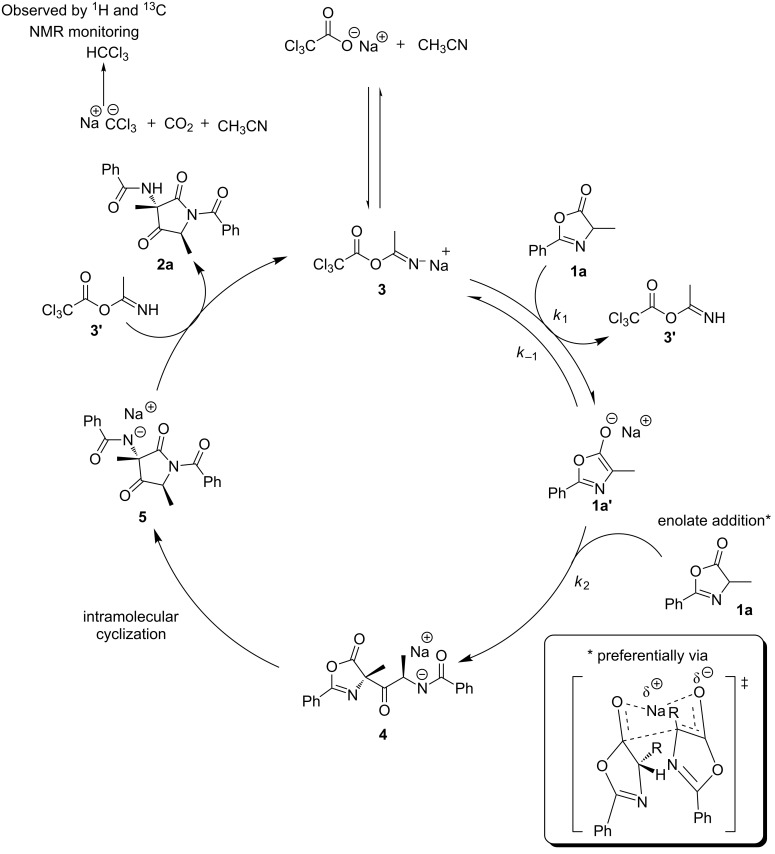
Plausible mechanism for the dimerization of azlactone.

Although the mechanistic details of dimerization remain to be elucidated, a plausible mechanism was proposed based on ^1^H NMR evidence (Figure 3). The NMR experiments revealed that KTCA reacts with CH_3_CN to form a basic species **3** [30]. The enolate azlactone **1a’** attacks the carbonyl carbon of azlactone **1a** forming intermediate **4** [27]. The diastereoselectivity is installed at this stage and kept the same until to the end of the reaction. Steric effects might be involved in this process and the biggest substituents positioned far away from each other, as shown in Figure 3. Furthermore, a six-membered ring involving the salt cation, the azlactone ring and the enolate might be involved in the addition step. Irreversible intramolecular cyclization of **4** gave **5** (for a reaction reversibility study of product **2h**, see Supporting Information File 1), eventually affording **2a** after protonation and thus regenerating the catalyst intermediate **3**. ^1^H NMR analysis using CD_3_CN showed high levels of HCCl_3_, which could be form after decarboxylation of the basic species.

In an attempt to obtain a better understanding at this dimerization reaction and to give new insights into this kind of transformation, we carried out some kinetics experiments. Taking into account the dimerization kinetic studies suggested by Mazurkiewicz et al. and experiments by NMR spectroscopy, we report here kinetic studies revealing mechanistic features on the dimerization reaction catalyzed by base species from trichloroacetate sodium salt [24].

The integrated rate law, suggested by Mazurkiewicz et al. was proposed for a ^3^/_2_ reaction order for the azlactone that results from the above mechanism, which is:

[1]
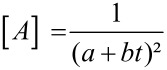


Where


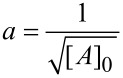


and


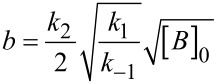


so that [A], [A]_0_ and [B]_0_ are, respectively, azlactone **1a** concentration, initial azlactone **1a** concentration, and initial base species **3** concentration; *k*_1_ and *k*_−1_ are the direct and reverse reaction rate constants for the formation of an azlactone enolate derivative **1’** and the azlactone **1a**, respectively.

**Figure 4:**
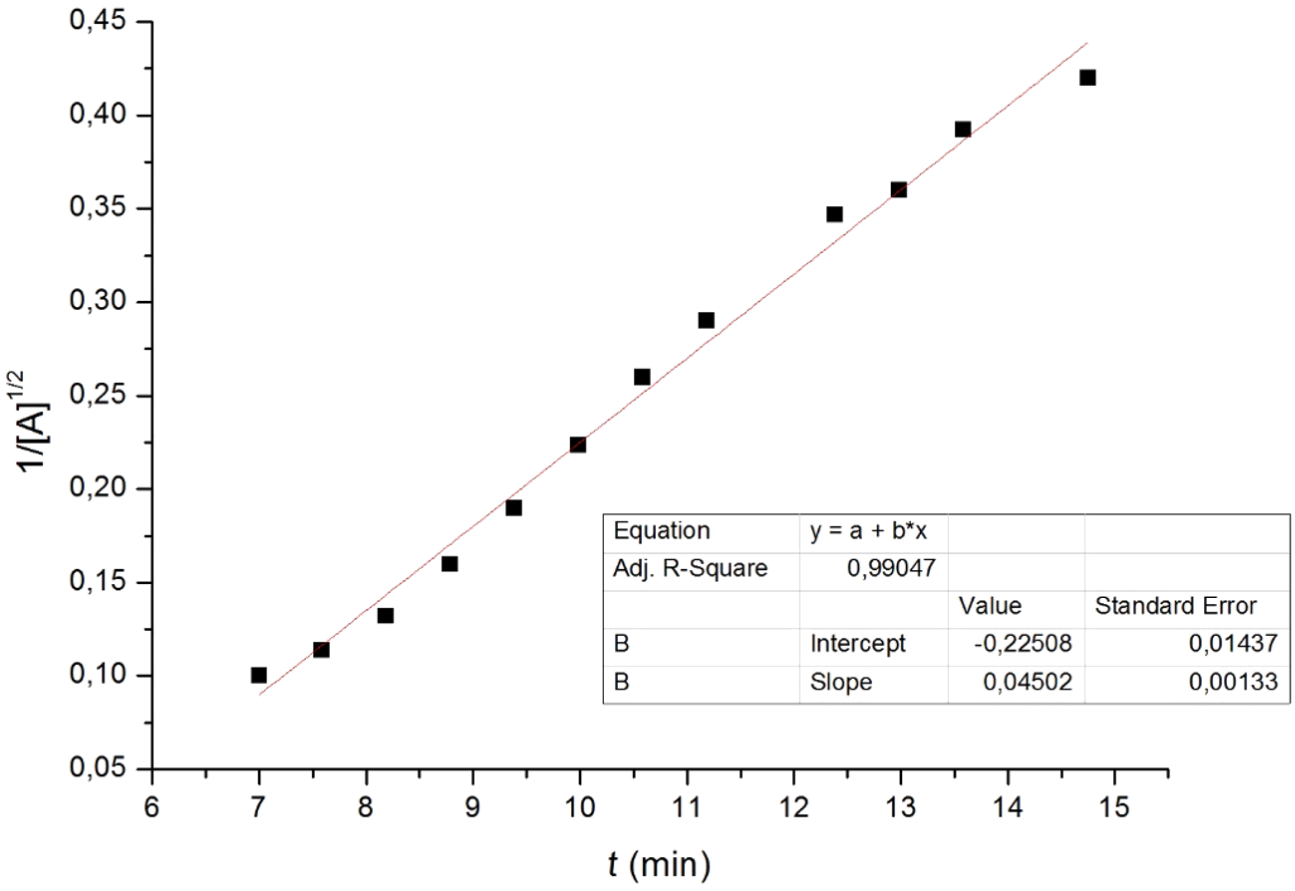
Plot of 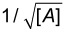 vs time for the dimerization of azlactone **1a**.

In situ reaction monitoring by NMR spectroscopy allowed us to follow the disappearance of the characteristic signal of the azlactone **1a** (q, 4.45 ppm) as well as the appearance of the characteristic signal of the dimer **2a** (q, 5.18 ppm). The values found were plotted in Figure 4 and a linear relationship between


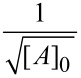


and the reaction time was obtained with high correlation factor (R^2^ = 0.9905).

We then reasoned the second order kinetic proﬁle to the global reaction, resulting from a ^3^/_2_ order for the azlactone **1a** and ^1^/_2_ order for the base species **3** as could be shown in Equation 2 (please, refer to Supporting Information File 1 for a demonstration of the discussed equations):

[2]
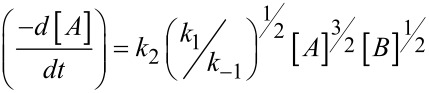


The ^3^/_2_ reaction order for azlactone has also been confirmed using the technique of initial rates varying the initial concentrations of azlactone **1a** (See Supporting Information File 1).

**Scheme 3:**
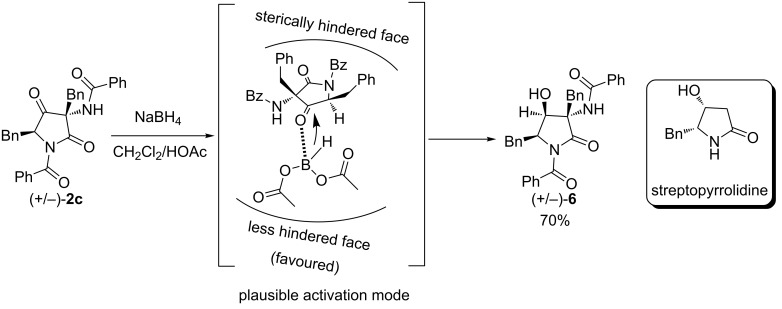
Reduction of **2c**.

Finally, the synthetic utility of the dimerization products was demonstrated by stereocontrolled reduction of the dimer **2c** (Scheme 3). Compound **2c** was dissolved in a mixture of acetic acid and NaBH_4_, cooled to 0 °C to afford the highly functionalized (+/−)-streptopyrrolidine analogue **6** in 70% yield as a unique diastereomer. Streptopyrrolidine was isolated from the marine bacterium *Streptomyces* sp. and has exhibited a significant anti-angiogenesis activity [31]. It is worth mentioning that this product has three consecutive stereocenters and different sites of variation from the original natural product.

**Figure 5:**
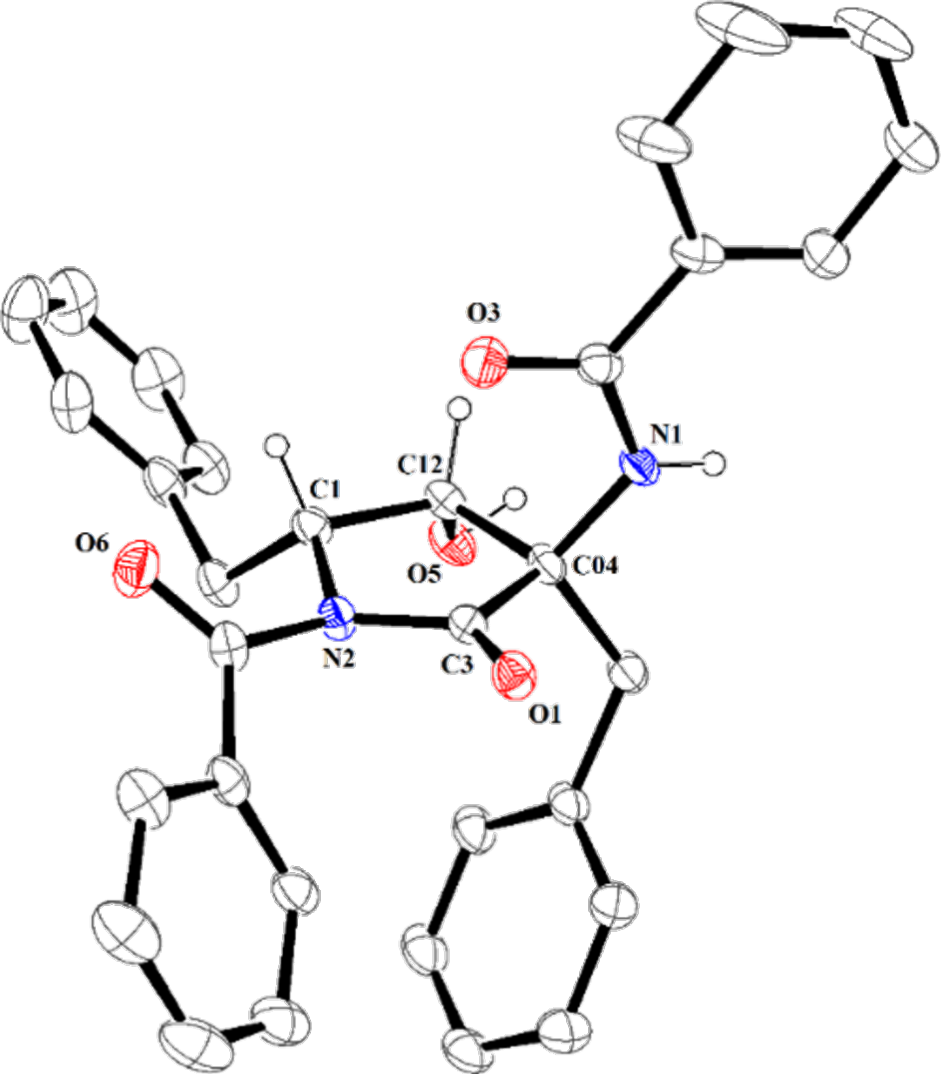
X-ray crystallographic structure of **6** (30% ellipsoids probability).

The hydride comes from the less hindered side of the ketone moiety, leading to the formation of a hydroxy group in *cis*-position relative to both the benzyl substituents [32]. The relative stereochemistry of **6** was assigned by X-ray analysis (Figure 5).

## Conclusion

In summary, we have demonstrated the first diastereoselective dimerization of azlactones mediated by a combination of acetonitrile and potassium or sodium trichloroacetate salts, which provides a straightforward and practical access to azlactone dimers with good to excellent yields and good diastereoselectivities. In addition, the relative stereochemistry of major azlactone dimer was assigned as being *trans* by X-ray analysis. A plausible mechanism was proposed by ^1^H NMR spectroscopic monitoring and revealed that the diastereoselectivity was determined during C–C bond formation step. A diastereoselective streptopyrrolidine analogue, containing three contiguous stereogenic centers, was smoothly obtained as a sole diastereomer in 70% yield.

## Supporting Information

File 1 Experimental procedures, characterization data, copies of ^1^H and ^13^C NMR spectra for the final products, NMR kinetic experiments as well as crystallographic data for **2a** and **6**. Demonstration of kinetic equations and results of the initial rate kinetic study.
