# Supplementary material for: A Brønsted base-promoted diastereoselective dimerization of azlactones
Source: Beilstein J Org Chem. 2017 Dec 13;13:2663–70. doi: 10.3762/bjoc.13.264 (PMC5753058; doi:10.3762/bjoc.13.264)
Supplement: File 1 — Experimental procedures, characterization data, copies of 1H and 13C NMR spectra for the final products, NMR kinetic experiments as well as crystallographic data for 2a and 6. Demonstration of kinetic equations and results of the initial rate kinetic study. [file Beilstein_J_Org_Chem-13-2663-s001.pdf]

Supporting Information

for

A Brønsted base-promoted diastereoselective dimerization of azlactones

Danielle L. J. Pinheiro, Gabriel M. F. Batista, Pedro P. de Castro, Leonã S. Flores, Gustavo F. S. Andrade and Giovanni W. Amarante*

Address: Chemistry Department, Federal University of Juiz de Fora, Cidade Universitária, São Pedro, Juiz de Fora, MG, CEP 36036-900, Brazil

Email: Giovanni W. Amarante - giovanni.amarante@ufjf.edu.br
*Corresponding author

Experimental procedures, characterization data, copies of ^{1}H and ^{13}C NMR spectra for the final products, NMR kinetic experiments as well as crystallographic data for 2a and 6. Demonstration of kinetic equations and results of the initial rate kinetic study

1. Experimental section S2
2. ^{1}H and ^{13}C NMR spectra of dimerization products S8
3. ^{1}H and ^{13}C NMR spectra of product **6** S21
4. Crude NMR data employed for dr measurement S24
5. Mechanism and kinetic experiments by NMR monitoring S29
6. Reaction reversibility evaluation S40
7. X-ray data S41
8. References S53

1- Experimental section

General information

Unless otherwise noted, all reagents were obtained commercially and used without further purification. Unless otherwise noted, all reaction mixtures were carried out in flame-dried flask under a positive pressure of dry nitrogen. Analytical thin layer chromatography (TLC) was performed on precoated glass-backed TLC plates (silica gel 60 F254) and visualized by UV lamp (254 nm). Yields refer to chromatographically purified or by recrystallization and spectroscopically pure compounds, unless stated otherwise. ^{1}H and ^{13}C spectra were recorded on a 500 MHz spectrometer. Chemical shifts are reported in ppm. ^{1}H NMR spectra were referenced to $CDCl_3$ (7.26 ppm) and ^{13}C NMR spectra were referenced to $CDCl_3$ (77.0 ppm). All ^{13}C spectra were measured with complete proton decoupling. Peak multiplicities are designated by the following abbreviations: s, singlet; d, doublet; dd, double doublet; t, triplet; m, multiplet; br, broad; and *J*, coupling constant in Hz. High-resolution mass spectra were acquired in the positive ion mode using a mass spectrometer equipped with an electrospray ionization source. Azlactones, were prepared according to literature procedure [1].

General procedure and characterization data for the dimerization of azlactones 2a–i

In a flamed-dried screw-cap vial, with 4 Å molecular sieves as additive, under nitrogen atmosphere, was added CH_3CN (to provide a solution 0.06 mol L^{-1} in azlactone), and azlactone (0.3 mmol). To this solution, potassium or sodium trichloroacetate salts (0.09 mmol) were added. The reaction was kept at room temperature for 1 h and monitored by thin layer chromatography. The crude reaction mixture was diluted with CH_2Cl_2 (10 mL) and washed with water (5 mL). The organic layer was dried over anhydrous Na_2SO_4, filtered off and concentrated under reduced pressure. At this point, an aliquot was dissolved in $CDCl_3$ and directly analyzed by ^{1}H NMR in order to see the corresponding dr. Then the major diastereomer was obtained after recrystallization or purification through column chromatography (hexanes/ethyl acetate 3:1).

(+/−) *N*-((3S,5S)-1-Benzoyl-3,5-dimethyl-2,4-dioxopyrrolidin-3-yl)benzamide (2a)

Diastereomeric ratio (dr) from ^{1}H NMR analysis of crude reaction mixture: 6/1 (trans/cis). The product was purified by recrystallization (EtOH/H_2O 5:1) to afford **2a** (major diastereomer, 22 mg, 84%) as a white solid (m.p. 226ºC). ^{1}H NMR (500 MHz, $CDCl_3$) δ: 7.74-7.72 (m, 2H), 7.70-7.68 (m, 2H), 7.56 (tt, 1H, *J* = 1.3 Hz, *J* = 5.0 Hz), 7.53 (tt, 1H, *J* = 1.3 Hz, *J* = 5.0 Hz), 7.47-7.44 (m, 2H), 7.42-7.39 (m, 2H), 6.85 (s, 1H), 5.18 (q, 1H, *J* = 7.0 Hz), 1.70 (s, 3H), 1.67 (d, 3H, *J* = 7.1 Hz); ^{13}C NMR (125 MHz, $CDCl_3$) δ: 205.7, 172.3, 169.7, 167.4, 134.4, 132.7, 132.5, 130.9, 128.8, 128.7, 128.1, 127.5, 60.8, 60.5, 19.6, 16.9. **2a'** (minor diastereomer, 3.5 mg, 14%). ^{1}H NMR (500 MHz, $CDCl_3$) δ: 7.83-7.79 (m, 4H), 7.58-7.53 (m, 2H), 7.48-7.41 (m, 4H), 6.69 (s, 1H), 4.94 (q, 1H, *J* = 8.0 Hz), 1.77 (d, 3H, *J* = 7.0 Hz), 1.57 (s, 3H); ^{13}C NMR (125 MHz, $CDCl_3$) δ: 206.0, 170.8, 170.3, 167.3, 134.2, 132.9, 132.7, 131.3, 129.8, 128.8, 128.0, 127.5, 60.1, 59.1, 19.1, 14.6. For IR and HRMS data, see reference [2].

(+/−) *N*-((3S,5S)-1-Benzoyl-3,5-diisobutyl-2,4-dioxopyrrolidin-3-yl)benzamide (2b)

Diastereomeric ratio (dr) from ^{1}H NMR analysis of crude reaction mixture: 4/1 (trans/cis). The product was purified by recrystallization (EtOH/H_2O 5:1) to afford **2b** (major diastereomer, 23 mg, 71%) as a white solid (m.p. 124ºC). ^{1}H NMR (500 MHz, $CDCl_3$) δ: 7.72-7.70 (m, 2H), 7.69-7.67 (m, 2H), 7.56 (tt, 1H, *J* = 1.2 Hz, *J* = 5.0 Hz), 7.52 (tt, 1H, *J* = 1.2 Hz, *J* = 5.0 Hz), 7.47-7.44 (m, 2H), 7.42-7.39 (m, 2H), 6.89 (s, 1H), 5.15 (dd, 1H, *J* = 4.7 Hz, *J* = 9.1 Hz), 2.10-2.02 (m, 2H), 1.94-1.92 (m, 2H), 1.85-1.71 (m, 2H), 1.14 (d, 3H, *J* = 6.6 Hz), 1.10-1.09 (m, 6H), 1.02 (d, 3H, *J* = 6.6 Hz); ^{13}C NMR (125 MHz, $CDCl_3$) δ: 204.3, 171.9, 169.9, 167.13, 134.7, 132.6, 132.5, 128.9, 128.7, 128.6, 128.1, 127.4, 64.5, 62.7, 41.8, 41.4, 25.1, 24.4, 24.2, 24.1, 23.1, 21.7.For IR and HRMS data, see reference [2].

(+/−) *N*-((3S,5S)-1-Benzoyl-3,5-dibenzyl-2,4-dioxopyrrolidin-3-yl)benzamide (2c)

Diastereomeric ratio (dr) from ^{1}H NMR analysis of crude reaction mixture: >19/1 (trans/cis). The product was purified by recrystallization (EtOH/H_2O 5:1) to afford **2c** (major diastereomer, 34 mg, 92%) as a white solid (m.p. 150ºC). ^{1}H NMR (500 MHz, $CDCl_3$) δ 7.75-7.73 (m, 2 H), 7.61 (tt, 1H, *J* = 1.3 Hz, *J* = 5.0 Hz), 7.52-7.46 (m, 5H), 7.43-7.38 (m, 3H), 7.36-7.33 (m, 4H), 7.30-7.28 (m, 1H), 7.23-7.21 (m, 2H), 7.19-7.17 (m, 2H), 6.72 (s, 1H), 5.52 (dd, 1H, *J* = 3.6 Hz, *J* = 5.7 Hz), 3.40 (dd, 1H, *J* = 5.7 Hz, *J* = 14.0 Hz), 3.28 (dd, 1H, *J* = 3.6 Hz, *J* = 14.0 Hz), 2.43 (d, 1H, *J* = 14.0 Hz), 2.26 (d, 1H, *J* = 14.0 Hz); ^{13}C NMR (125 MHz, $CDCl_3$) δ: 204.3, 171.8, 170.0, 167.0, 135.9,

134.5, 132.7, 132.6, 131.9, 131.0, 130.7, 130.4, 129.2, 128.7, 128.6, 128.5, 128.1, 127.7, 127.2, 66.8, 62.2, 36.4, 34.0. For IR and HRMS data, see reference [3].

(+/−) *N*-((3S,5S)-1-Benzoyl-3,5-bis(4-methylbenzyl)-2,4-dioxopyrrolidin-3-yl)benzamide (2d)

Diastereomeric ratio (dr) from ^{1}H NMR analysis of crude reaction mixture: >19/1 (trans/cis). The product was purified by column chromatography on silica gel (hexanes/EtOAc 3:1) to afford **2d** (major diastereomer, 36 mg, 93%) as a white solid (m.p. 140°C). IR (TlBr-TlI): 1720, 1683, 1508, 1468, 1258. ^{1}H NMR (500 MHz, $CDCl_3$) δ: 7.73-7.71 (m, 2H), 7.53-7.46 (m, 6H), 7.36-7.27 (m, 2H) 7.21-7.20 (m, 2H), 7.14-7.04 (m, 6H), 6.51 (s, 1H), 5.46 (dd, 1H, *J* = 3.5 Hz, *J* = 5.6 Hz), 3.32 (dd, 2H, *J* = 5.7 Hz, *J* = 14.4 Hz), 3.23 (dd, 2H, *J* = 3.5 Hz, *J* = 14.4 Hz), 2.35 (s, 3H), 2.29 (s, 3H); ^{13}C NMR (125 MHz, $CDCl_3$) δ: 204.4, 171.9, 170.1, 167.0, 138.4, 137.3, 134.5, 132.7, 132.6, 132.5, 131.1, 130.5, 130.2, 129.9, 129.7, 129.5, 129.3, 129.2, 129.0, 128.8, 128.6, 128.1, 127.5, 127.3, 66.9, 62.2, 35.9, 33.5, 21.1, 21.0; HRMS: calcd for $[C34H30N2O4]^+$ ($[M+Na]^+$): *m/z* 553.2103, found 553.2092.

(+/−)*N*-((3S,5S)-3,5-Diisobutyl-1-(4-nitrobenzoyl)-2,4-dioxopyrrolidin-3-yl)-4-nitrobenzamide (2e)

Diastereomeric ratio (dr) from ^{1}H NMR analysis of crude reaction mixture: 4/1 (trans/cis). The product was purified by column chromatography on silica gel (hexanes/EtOAc 3:1) to afford **2e** (major diastereomer, 36 mg, 60%) as a white oil. IR (TlBr-TlI): 1749, 1650, 1635, 1521, 1461, 1283. ^{1}H NMR (500 MHz, $CDCl_3$) δ: 8.32 (d, 2H, *J* = 8.8 Hz), 8.30 (d, 2H, *J* = 8.8 Hz), 7.90 (d, 2H, *J* = 8.9 Hz), 7.80 (d, 2H, *J* = 8.9 Hz), 6.79 (s, 1H), 5.10 (dd, 1H, *J* = 4.7, *J* = 9.6 Hz), 2.09-2.04 (m, 2H), 2.01-1.99 (m, 2H), 1.97-1.91 (m, 2H), 1.19 (d, 3H, *J*= 6.0 Hz), 1.15-1.14 (m, 6H), 1.05 (d, 3H, *J* = 6.6 Hz); ^{13}C NMR (125 MHz, $CDCl_3$) δ: 203.0, 171.4, 168.0, 165.4, 150.3, 149.7, 140.3, 136.5, 129.4, 128.6, 124.0, 64.6, 62.8, 41.9, 41.2, 29.7, 25.0, 24.5, 24.2, 23.2, 21.5; HRMS: calcd for $[C26H28N4O8]^+$ ($[M+H]^+$): *m/z* 525.1985, found 525.1971.

(+/−) *N*-((3S,5S)-3,5-Diallyl-1-benzoyl-2,4-dioxopyrrolidin-3-yl)benzamide (2f)

Diastereomeric ratio (dr) from ^{1}H NMR analysis of crude reaction mixture: 4/1 (trans/cis). The product was purified by column chromatography on silica gel (hexanes/EtOAc 3:1) to afford **2f** (mixture of diastereomer, 13 mg, 78%) as a white oil. IR (TlBr-TlI): 1743, 1688, 1642, 1524, 1283. ^{1}H NMR (500 MHz, $CDCl_3$) δ: 7.87-7.85 (m, 2H minor), 7.80-7.78 (m, 2H minor), 7.72-7.68 (m, 3H major + 4H minor), 7.59-7.55 (m, 1H major + 2H minor), 7.52 (tt, *J* = 1.0 Hz, *J* = 7.5 Hz, 1H major), 7.48-7.39 (m, 5H

major), 6.77 (s, 1H major), 6.74 (s, 1H minor), 6.17-6.09 (m, 1H minor), 6.04-5.96 (m, 1H major), 5.87-5.76 (m, 1H major + 1H minor), 5.46-5.44 (m, 1H major), 5.40-5.37 (m, 2H minor), 5.32-5.30 (m, 1H major), 5.25 (dd, *J* = 1.5 Hz, *J* = 10.0 Hz, 1H major), 5.19 (dd, *J* = 1.5 Hz, *J* = 17.0 Hz, 1H major), 5.16-5.08 (m, 2H minor), 4.89 (dd, *J* = 4.5 Hz, *J* = 6.5 Hz, 1H minor), 3.08-3.03 (m, 1H minor), 3.01-2.96 (m, 1H major), 2.91-2.89 (m, 1H major), 2.88-2.86 (m, 1H minor), 2.81 (dd, *J* = 10.0 Hz, *J* = 15.0 Hz, 1H major + 1H minor), 2.72 (dd, *J* = 10.0 Hz, *J* = 15.0 Hz, 1H major + 1H minor); ^{13}C NMR (125 MHz, $CDCl_3$) δ: 204.36, 171.78, 169.95, 167.09, 134.40, 133.11, 132.97, 132.69, 132.66, 132.60, 131.99, 131.05, 130.15, 129.35, 128.99, 128.73, 128.71, 128.30, 128.19, 127.94, 127.46, 127.33, 123.08, 122.53, 120.93, 119.04, 65.11, 62.72, 61.66, 37.81, 35.94, 34.06, 33.12; HRMS: calcd for $[C24H22N2O4]^+$ ($[M+Na]^+$): *m/z* 425.1477, found 425.1470.

(+/−)-*N*-((3S,5S)-1-Benzoyl-3,5-bis(2-(methylthio)ethyl)-2,4-dioxopyrrolidin-3-yl)benzamide (2g)

Diastereomeric ratio (dr) from ^{1}H NMR analysis of crude reaction mixture: 4/1 (trans/cis). The product was purified by column chromatography on silica gel (hexanes/EtOAc 4:1) to afford **2g** (major diastereomer, 23 mg, 66%) as a white oil. IR (TlBr-TlI): 1743, 1704, 1634, 1508, 1283. ^{1}H NMR (500 MHz, $CDCl_3$) δ: 8.66 (s, 1H), 7.79 (d, 2H, *J* = 8.0 Hz), 7.71 (d, 2H, *J* = 8.0 Hz), 7.58-7.52 (m, 2H), 7.48-7.42 (m, 4H), 5.29 (dd, 1H, *J* = 4.0 Hz, *J* = 8.0 Hz), 2.96 (t, 2H, *J* = 6.0 Hz), 2.77-2.76 (m, 1H), 2.66-2.63 (m, 1H), 2.44-2.39 (m, 4H), 2.29 (s, 3H), 2.15 (s, 3H); ^{13}C NMR (125 MHz, $CDCl_3$) δ: 204.3, 171.4, 169.9, 167.2, 134.3, 132.7, 132.6, 130.9, 128.9, 128.7, 128.2, 127.5, 63.8, 63.6, 30.2, 29.9, 29.8, 27.4, 15.8, 15.2; HRMS: calcd for $[C24H26N2O4S2]^+$ ($[M+H]^+$): *m/z* 471.1412, found 471.1387.

(+/−) 4-Bromo-*N*-((3*S*,5*S*)-3,5-dibenzyl-1-(4-bromobenzoyl)-2,4-dioxopyrrolidin-3-yl)benzamide (2h)

Diastereomeric ratio (dr) from ^{1}H NMR analysis of crude reaction mixture: >19/1 (trans/cis). The product was purified by column chromatography on silica gel (hexanes/EtOAc 3:1) to afford **2h** (major diastereomer, 40 mg, 88%) as a yellow solid (m.p. 163ºC). IR (TlBr-TlI): 3263, 1749, 1678, 1622, 1587, 1520, 1266, 1230, 743. ^{1}H NMR (500 MHz, $CDCl_3$) δ: 8.80 (s, 1H), 7.59 (t, 1H, *J* = 6.6 Hz), 7.55-7.46 (m, 5H), 7.39 (d, 1H, *J* = 8.4 Hz), 7.33-7.19 (m, 8H), 7.08-7.03 (m, 3H), 5.36 (dd, 1H, *J* = 3.9 Hz,

J = 5.6 Hz), 3.25 (dd, 1H, *J* = 6.9 Hz, *J* = 14.3 Hz), 3.06 (dd, 1H, *J* = 4.3 Hz, *J* = 14.3 Hz), 2.36 (d, 1H, *J* = 14.0 Hz), 2.19 (d, 1H, *J* = 14.1 Hz); ^{13}C NMR (125 MHz, $CDCl_3$) δ: 204.2, 171.9, 169.2, 166.3, 135.9, 133.3, 132.1, 131.8, 131.6, 130.9, 130.7, 130.5, 130.0, 129.8, 129.4, 129.2, 128.9, 128.8, 128.4, 127.9, 127.7, 126.9, 67.0, 62.3, 36.6, 34.2; HRMS: calcd for $[C32H25Br2N2O4]^+$ ($[M+H]^+$): *m/z* 659.0181, found 659.0181.

(+/−) *N*-((3S,5S)-1-Benzoyl-3,5-diphenyl-2,4-dioxopyrrolidin-3-yl)benzamide (2i)

Diastereomeric ratio (dr) from ^{1}H NMR analysis of crude reaction mixture: >19/1 (trans/cis). The product was purified by column chromatography on silica gel (hexanes/EtOAc 3:1) to afford **2i** (major diastereomer, 19 mg, 63%) as a yellow oil. ^{1}H NMR (500 MHz, $CDCl_3$) δ: 8.35-8.29 (m, 1H), 8.17-8.11 (m, 1H), 8.02-7.97 (m, 1H), 7.93 (d, 1H, *J* = 7.3 Hz), 7.86 (t, 1H, *J* = 7.4 Hz), 7.82-7.80 (m, 1H), 7.70-7.68 (m, 2H), 7.44-7.51 (m, 8H), 7.37-7.34 (m, 5H), 5.77 (d, 1H, *J* = 6.8 Hz); ^{13}C NMR (125 MHz, $CDCl_3$) δ: 199.4, 170.3, 167.3, 166.4, 134.3, 133.7, 133.4, 133.1, 132.7, 130.6, 129.9, 129.0, 128.9, 128.8, 128.7, 128.2, 128.1, 128.0, 127.5, 126.7, 68.7, 67.0; HRMS: calcd for $[C30H23N2O4]^+$ ($[M+H]^+$): *m/z* 475.1658, found 475.1636. For IR and HRMS data, see reference [2].

4-Isopropyl-2-phenyloxazol-5-ol (2j)

The product was purified by column chromatography on silica gel (hexanes/EtOAc 3:1) to afford **2j** (53 mg, 79%) as a white solid (m.p. 125ºC). IR (TlBr-TlI): 3255, 2957, 2918, 1723, 1670, 1495, 1483, 1450, 1269, 1138. ^{1}H NMR (500 MHz, $CDCl_3$) δ: 8.44 (s, 1H), 7.92-7.81 (m, 2H), 7.66-7.59 (m, 1H), 7.57-7.48 (m, 2H), 3.67 (hept, 1H, *J* = 6.9 Hz), 1.29 (d, 6H, *J* = 6.8 Hz); ^{13}C NMR (125 MHz, $CDCl_3$) δ: 179.9, 165.2, 133.3, 133.2, 129.2, 127.7, 35.1, 18.9; HRMS: calcd for $[C24H25N2O4]^+$ ($[dimer+H]^+$): *m/z* 405.1814, found 405.1797.

(*S*)-4-*sec*-Butyl-2-phenyloxazol-5-ol (2k)

The product was purified by column chromatography on silica gel (hexanes/EtOAc 3:1) to afford **2k** (48 mg, 76%) as a green solid (m.p. 116ºC). IR (TlBr-TlI): 3269, 2970, 2924, 2872, 1723, 1670, 1646, 1509, 1483, 1450, 1261, 1138. ^{1}H NMR (500 MHz, $CDCl_3$) δ: 8.76 (s, 1H), 7.92-7.80 (m, 2H), 7.59 (td, 1H, *J* = 0.9 Hz, *J* = 7.7 Hz), 7.48 (t,

2H, J = 7.7 Hz), 3.49 (h, 1H, J = 6.8 Hz), 1.90-1.76 (m, 1H), 1.57-1.43 (m, 1H), 1.23 (dd, 3H, J = 0.6 Hz, J = 6.8 Hz), 0.97 (t, 3H, J = 7.2 Hz); ^{13}C NMR (125 MHz, $CDCl_3$) δ: 179.7, 165.6, 133.2, 129.0, 128.7, 127.8, 127.5, 41.7, 26.6, 16.4, 11.7; HRMS: calcd for [C26H29N2O4]$^+$ ([dimer+H]$^+$): *m/z* 433.2127, found 433.2110.

Preparation and characterization of product 6

Product **2c** (40 mg, 0.12 mmol) was dissolved in acetic acid/dichloromethane (242.4:3.5, 0.24 mL), cooled to 0 °C, and $NaBH_4$ (5.5 mg, 0.14 mmol) was added. The reaction mixture was stirred for 3 h. The crude reaction mixture was diluted with CH_2Cl_2 (5 mL) and washed with water (10 mL). The organic layer was dried over anhydrous Na_2SO_4, filtered off and concentrated under reduced pressure. At this point, the crude reaction mixture was dissolved in $CDCl_3$ and directly send to ^{1}H NMR in order to see the corresponding dr.

Diastereomeric ratio from ^{1}H NMR analysis of crude reaction mixture: >19:1 (trans/cis). The crude product was subjected to flash column chromatography (hexanes/AcOEt 3:1) and the compound **6** was obtained as a white solid, 28 mg, 70% yield (m.p. 140ºC). IR (TlBr-TlI): 3352, 1719, 1680, 1648, 1618, 1266. ^{1}H NMR (500 MHz, $CDCl_3$) δ: 7.62-7.60 (m, 2H), 7.56 (tt, 1H, J = 1.2 Hz, J = 7.5 Hz), 7.51-7.49 (m, 3H), 7.45- 7.42 (m, 4H), 7.37-7.34 (m, 2H), 7.29-7.26 (m, 3H), 7.25-7.24 (m, 2H), 7.19 (tt, 1H, J = 1.2 Hz, J = 7.3 Hz), 7.13-7.11 (m, 2H), 6.68 (s, 1H), 5.56 (d, 1H, J = 1.7 Hz), 5.20 (dt, 1H, J = 6.2 Hz, J = 9.3 Hz), 4.84 (dd, 1H, J = 1.6 Hz, J = 9.3 Hz), 3.89 (d, 1H, J = 14.5 Hz), 3.68 (dd, 1H, J = 6.6 Hz, J = 14.0 Hz), 3.15 (d, 1H, J = 14.4 Hz), 3.09 (dd, 1H, J = 5.9 Hz, J = 14.0 Hz); ^{13}C NMR (125 MHz, $CDCl_3$) δ: 173.3, 170.0, 168.7, 138.4, 134.7, 134.2, 133.1, 132.8, 132.4, 130.5, 130.1, 128.8, 128.5, 128.4, 128.2, 127.5, 127.0, 126.9, 73.7, 67.4, 58.9, 37.0, 36.2; HRMS: calcd for $[C_{32}H_{28}N_2O_4]^+$ ([M+H]$^+$): *m/z* 505.2127, found 505.2112.

2- ^{1}H and ^{13}C NMR spectra of dimerization products:

^{1}H NMR of **2a** ($CDCl_3$, 500 MHz).

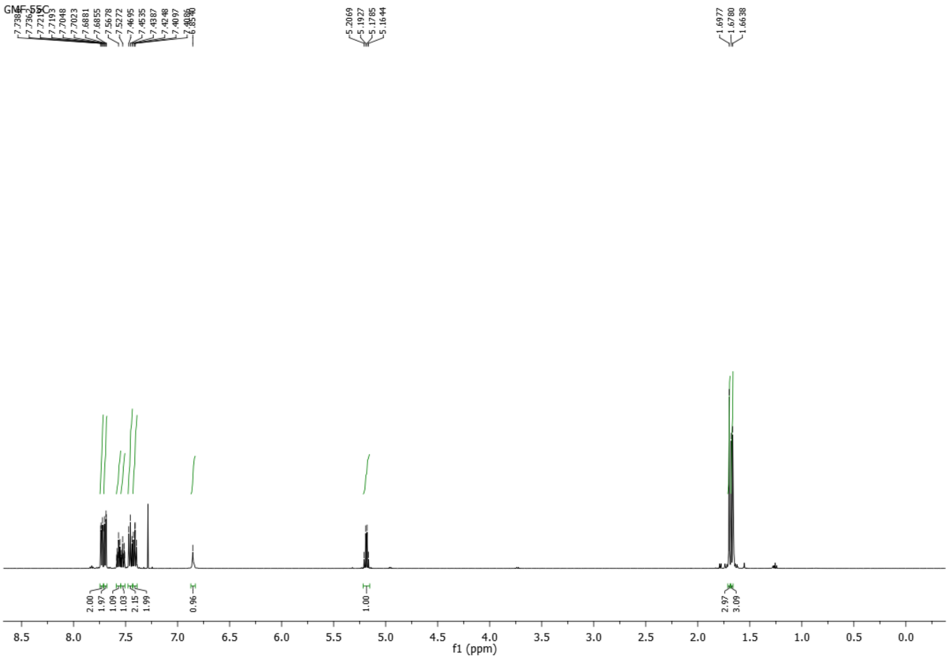

^{13}C NMR of **2a** ($CDCl_3$, 125 MHz).

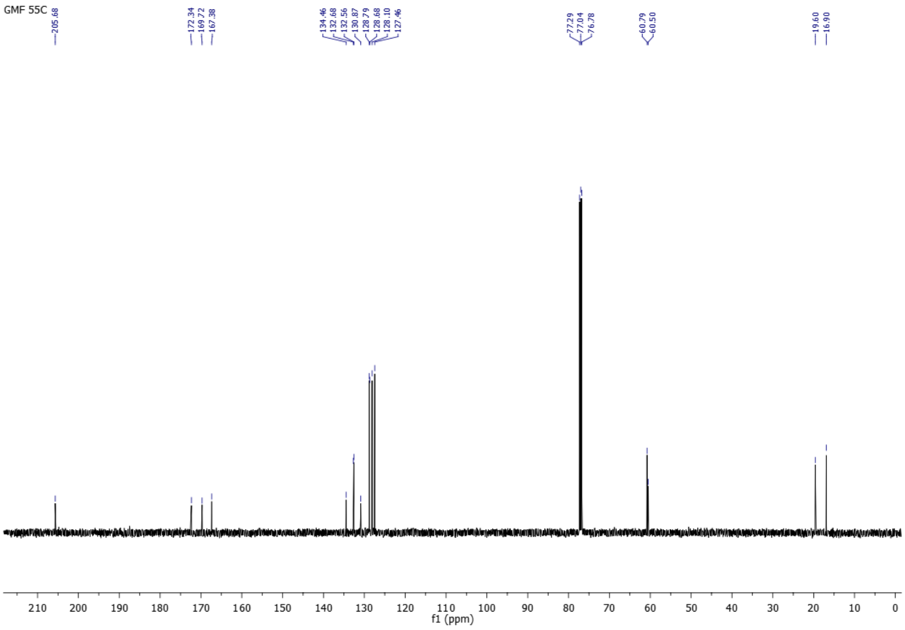

¹H NMR of **2a’** ($CDCl_3$, 500 MHz).

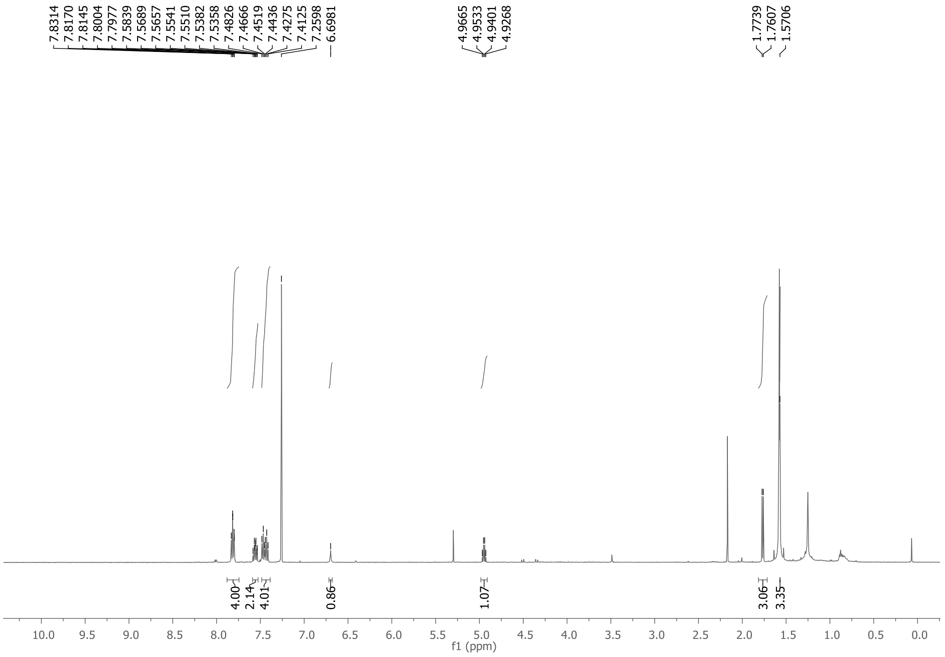

¹³C NMR of **2a’** ($CDCl_3$, 125 MHz).

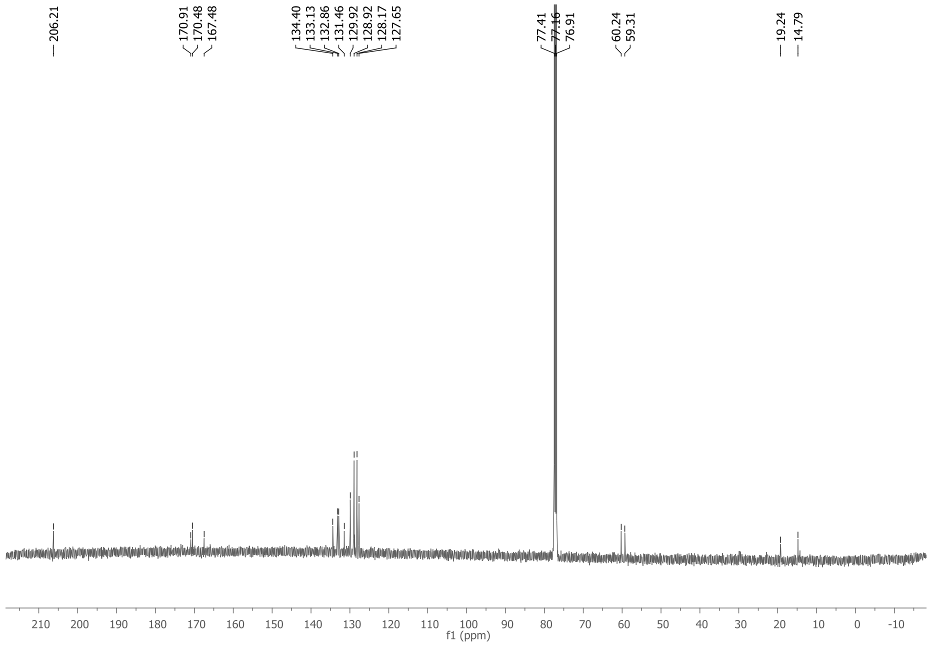

^{1}H NMR of **2b** ($CDCl_3$, 500 MHz).

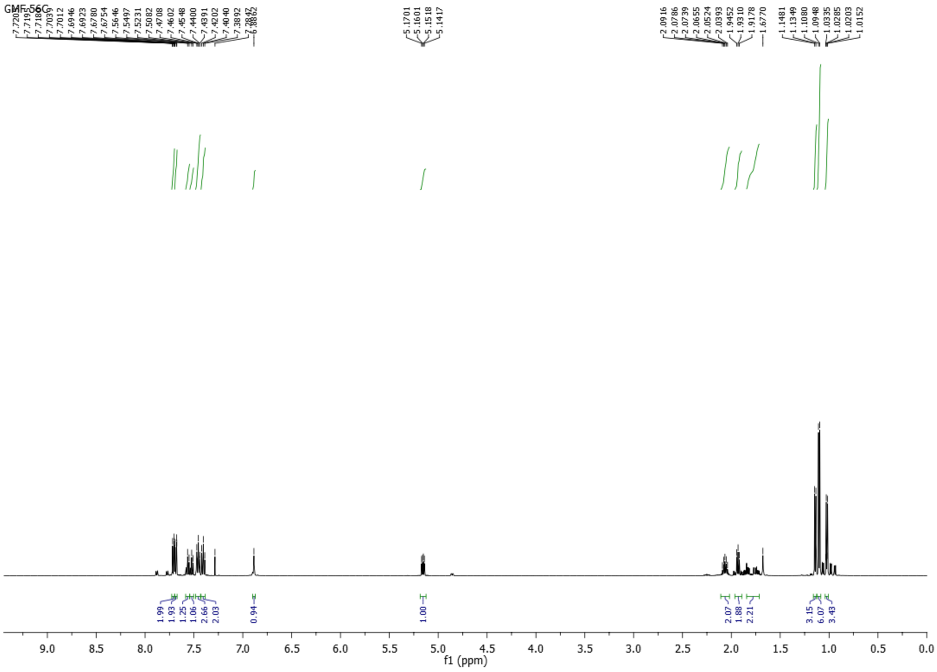

^{13}C NMR of **2b** ($CDCl_3$, 125 MHz).

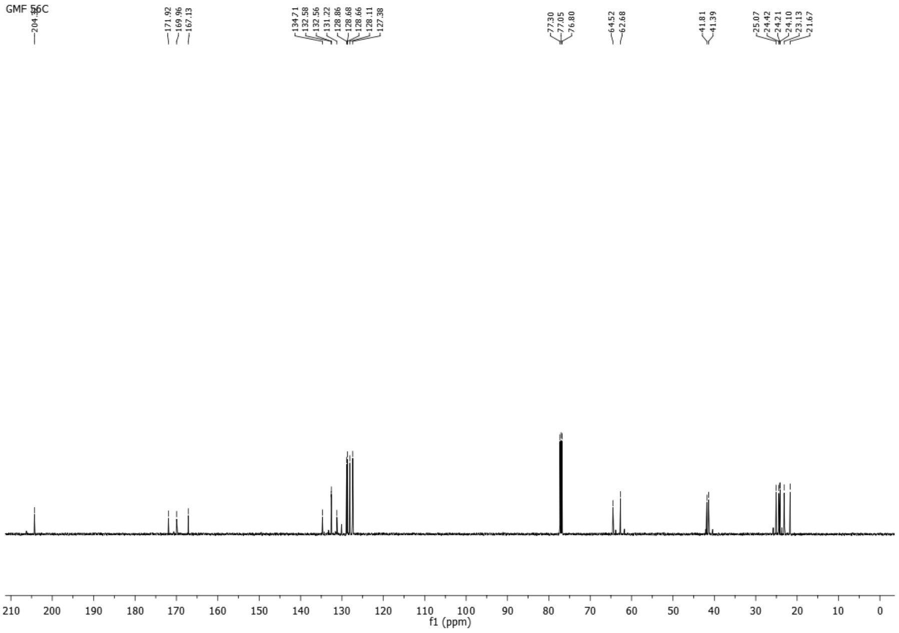

^{1}H NMR of **2c** ($CDCl_3$, 500 MHz).

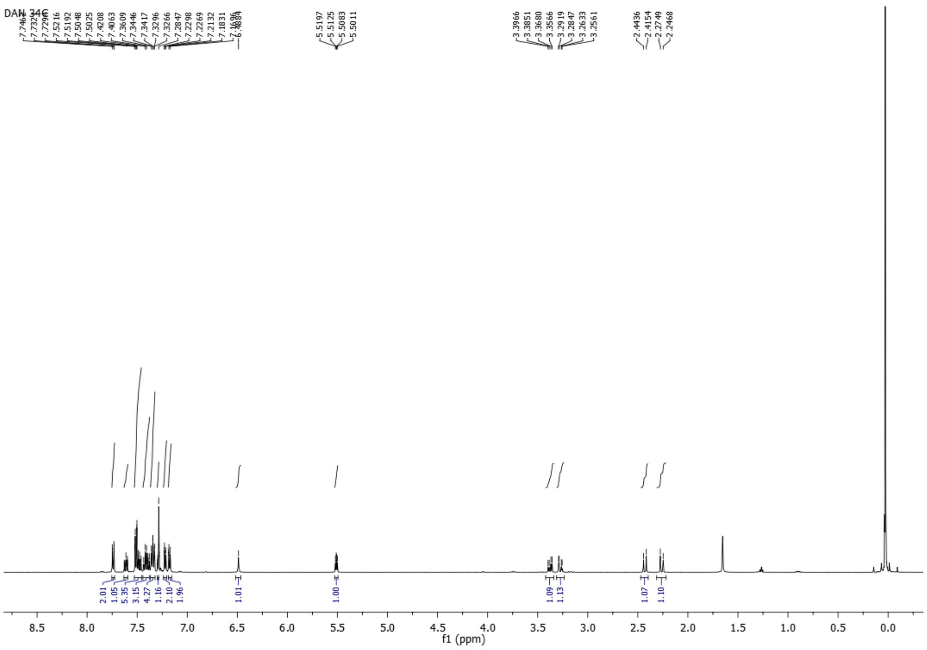

^{13}C NMR of **2c** ($CDCl_3$, 125 MHz).

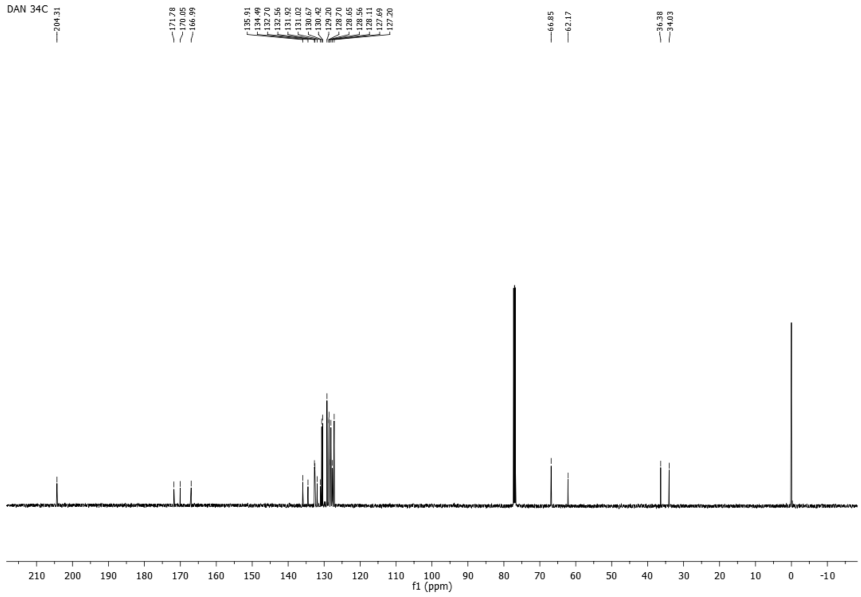

^{1}H NMR of **2d** ($CDCl_3$, 500 MHz).

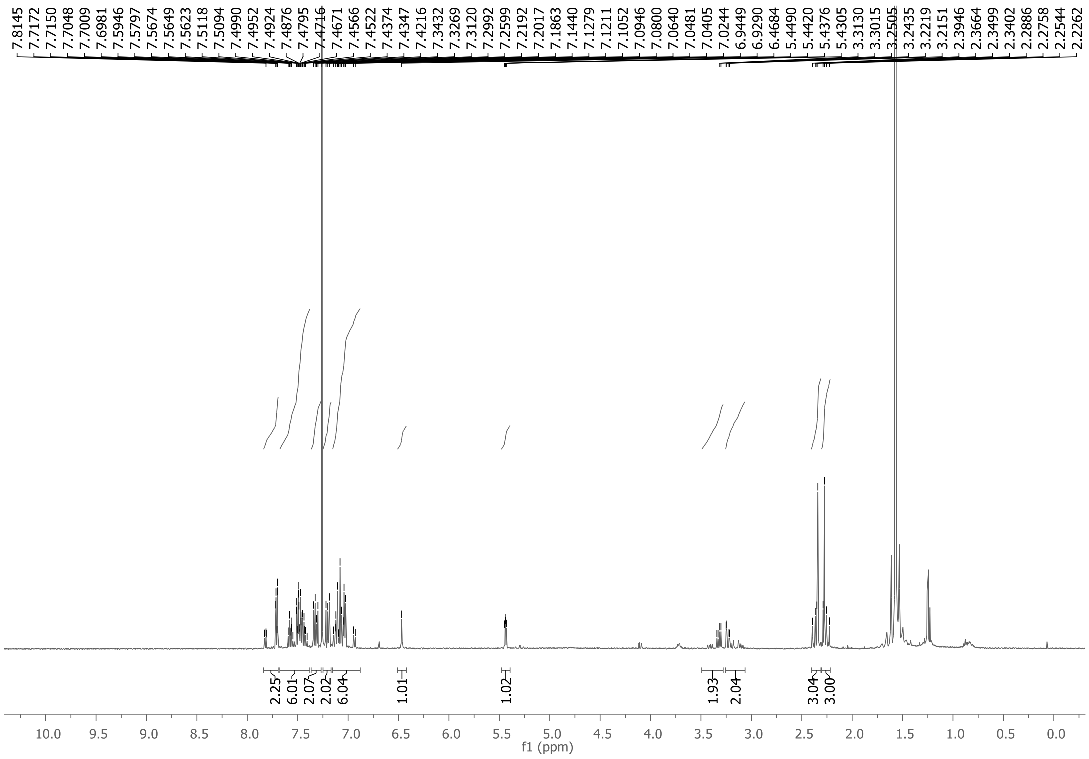

^{13}C NMR of **2d** ($CDCl_3$, 125 MHz).

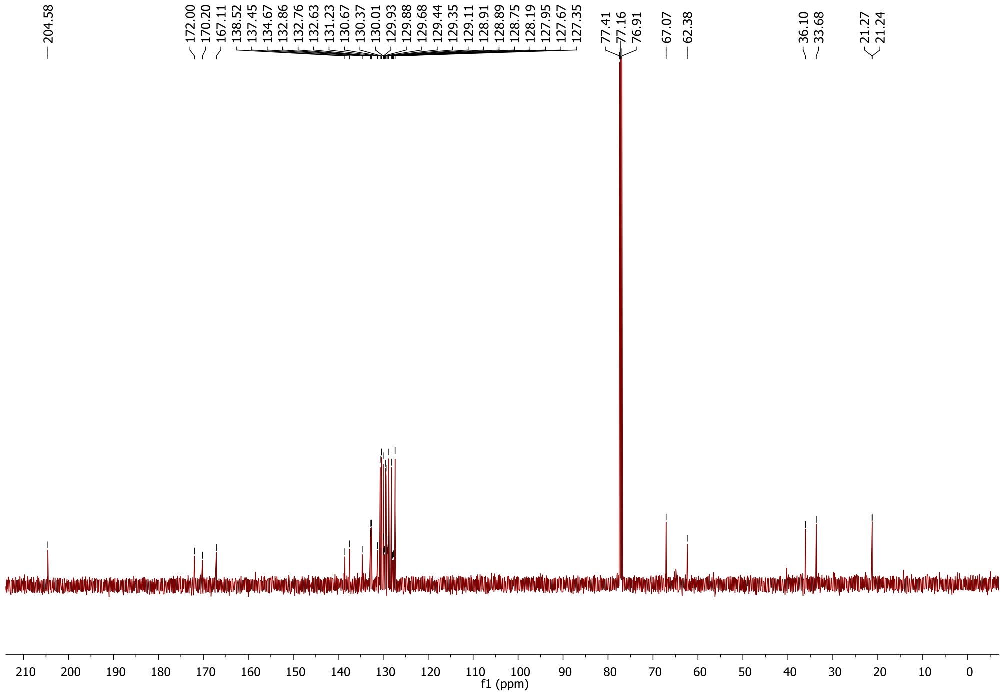

^{1}H NMR of **2e** ($CDCl_3$, 500 MHz).

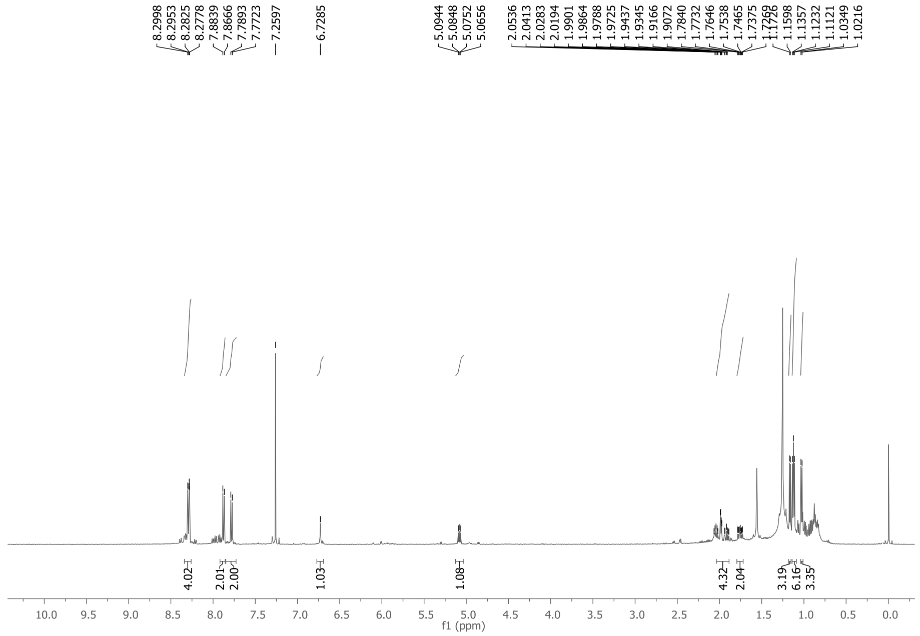

^{13}C NMR of **2e** ($CDCl_3$, 125 MHz).

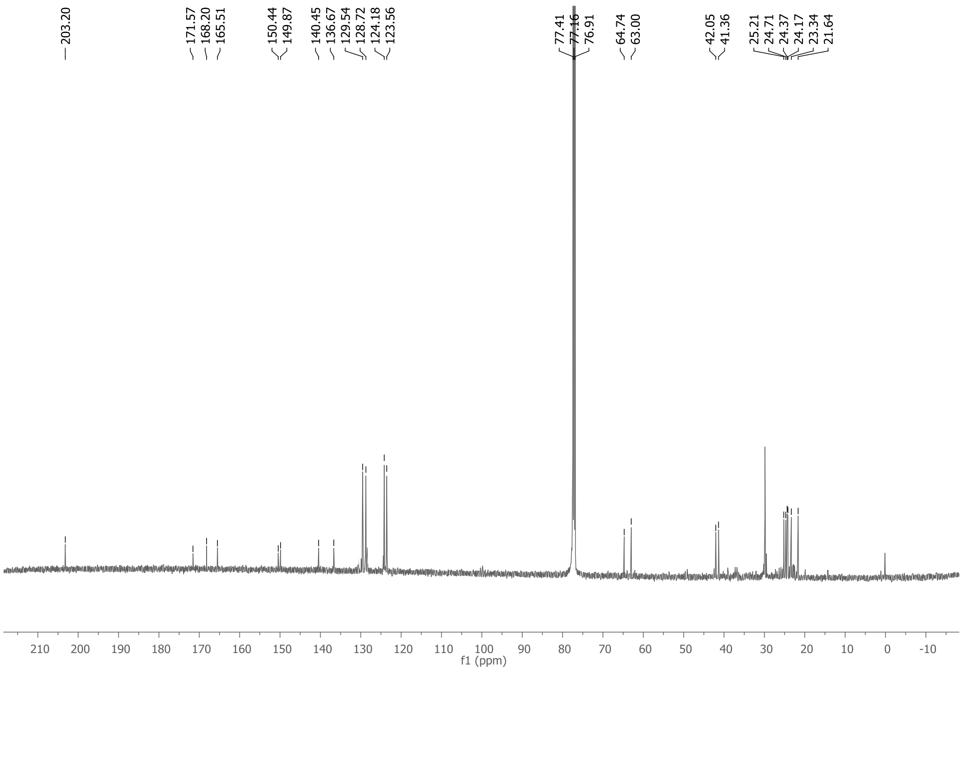

^{1}H NMR of **2f** ($CDCl_3$, 500 MHz).

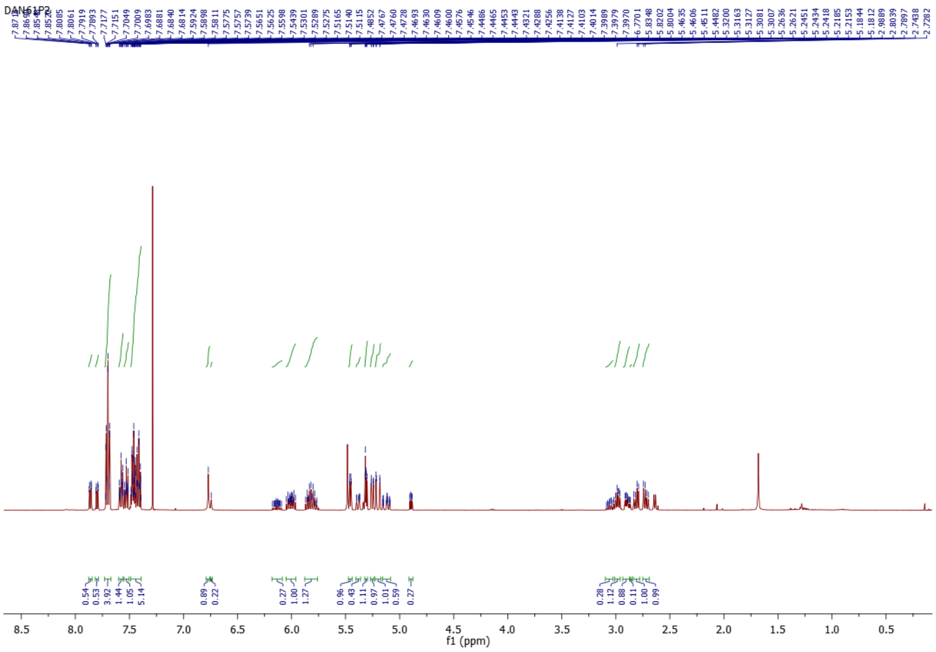

^{13}C NMR of **2f** ($CDCl_3$, 125 MHz).

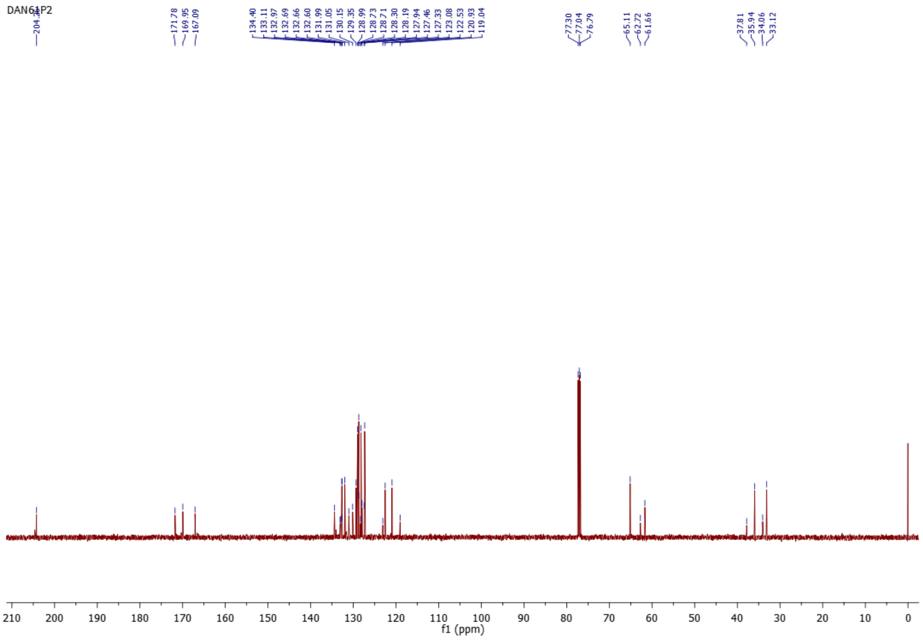

^{1}H NMR of **2g** ($CDCl_3$, 500 MHz).

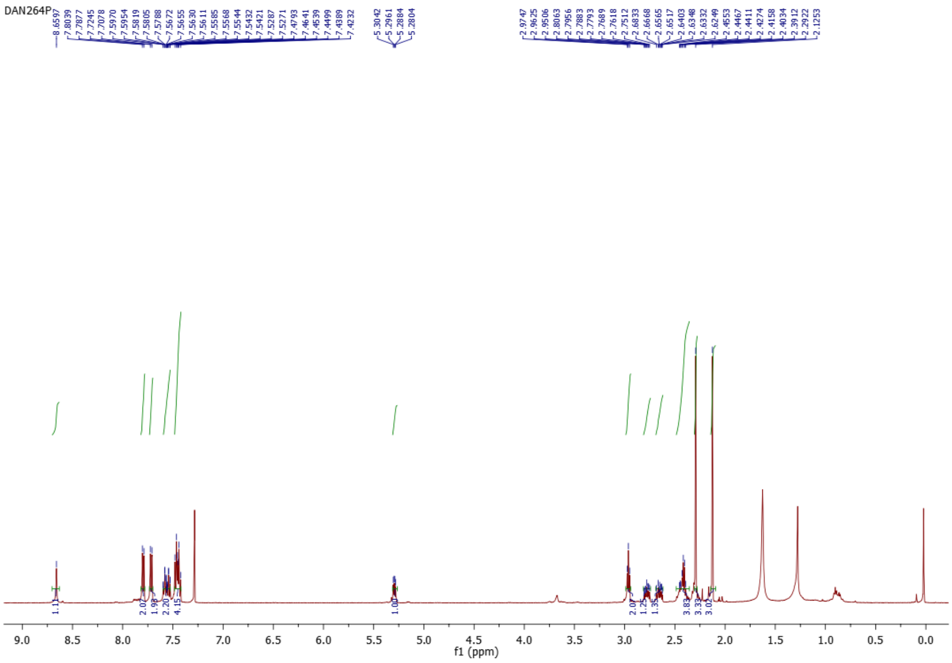

^{13}C NMR of **2g** ($CDCl_3$, 125 MHz).

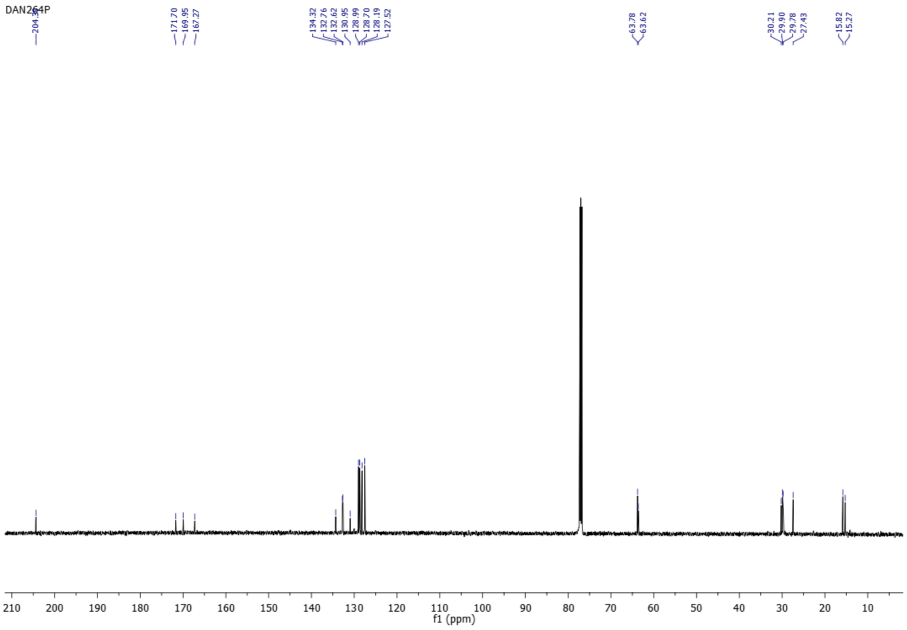

^{1}H NMR of **2h** ($CDCl_3$, 500 MHz).

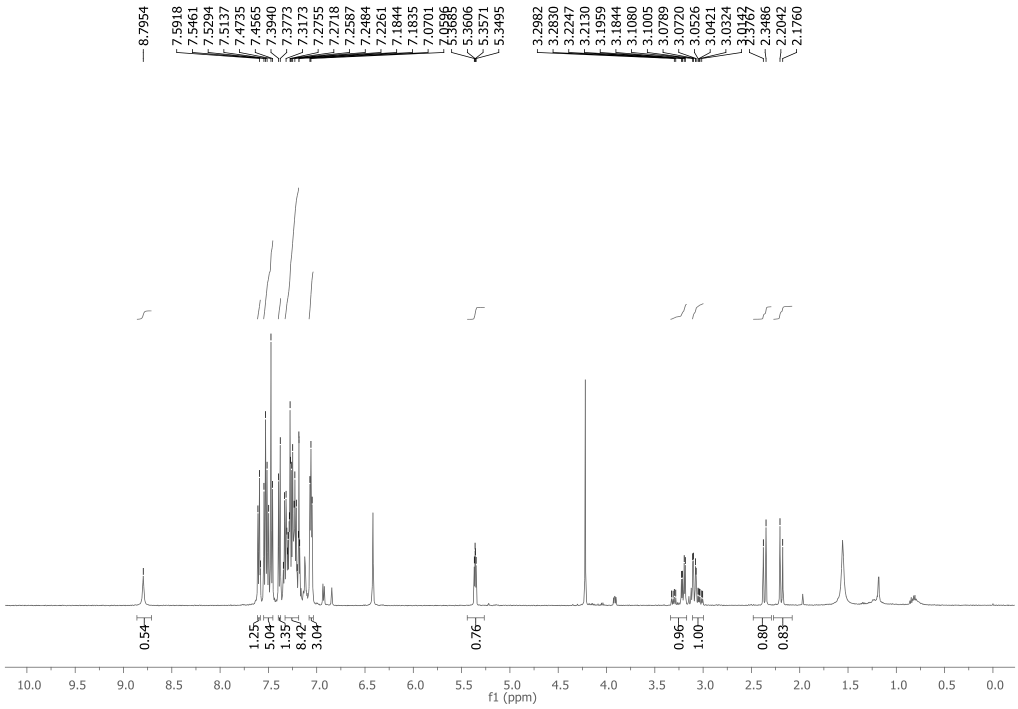

^{13}C NMR of **2h** ($CDCl_3$, 125 MHz).

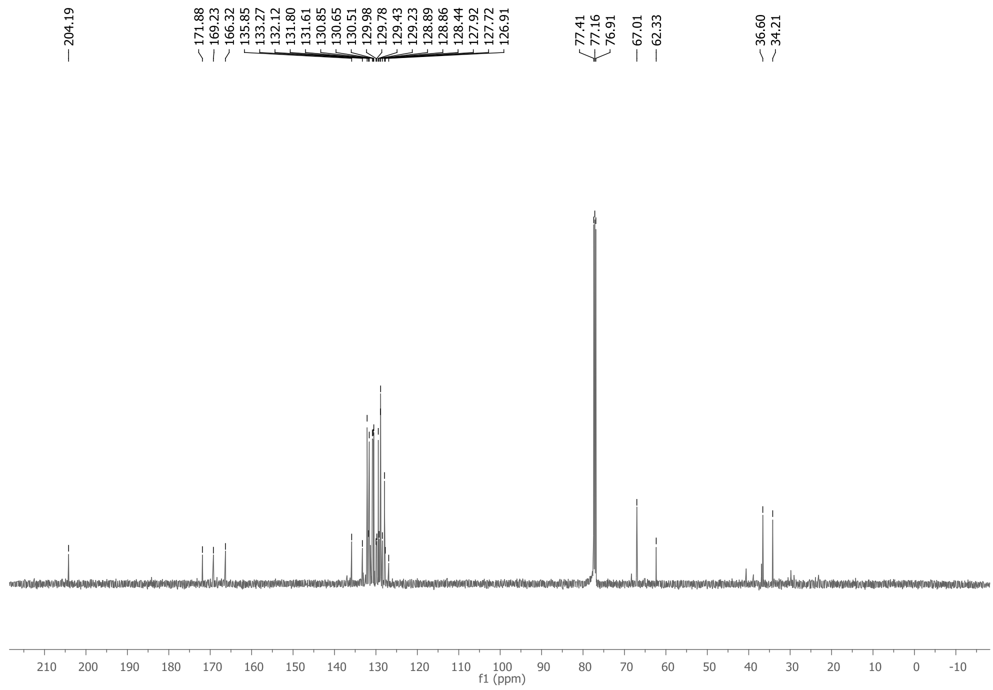

^{1}H NMR of **2i** ($CDCl_3$, 500 MHz).

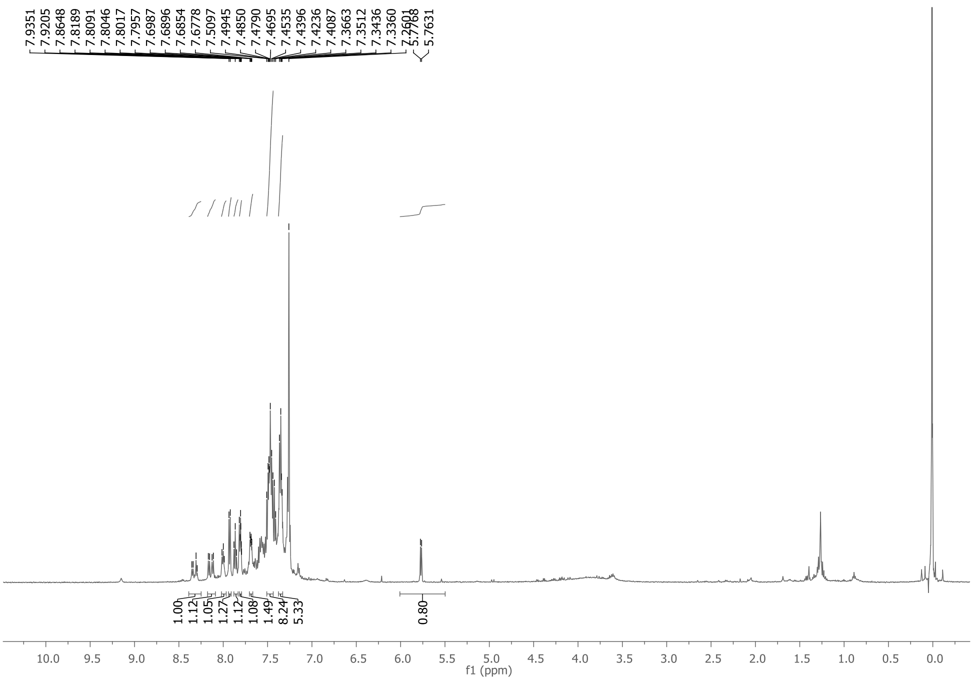

^{13}C NMR of **2i** ($CDCl_3$, 125 MHz).

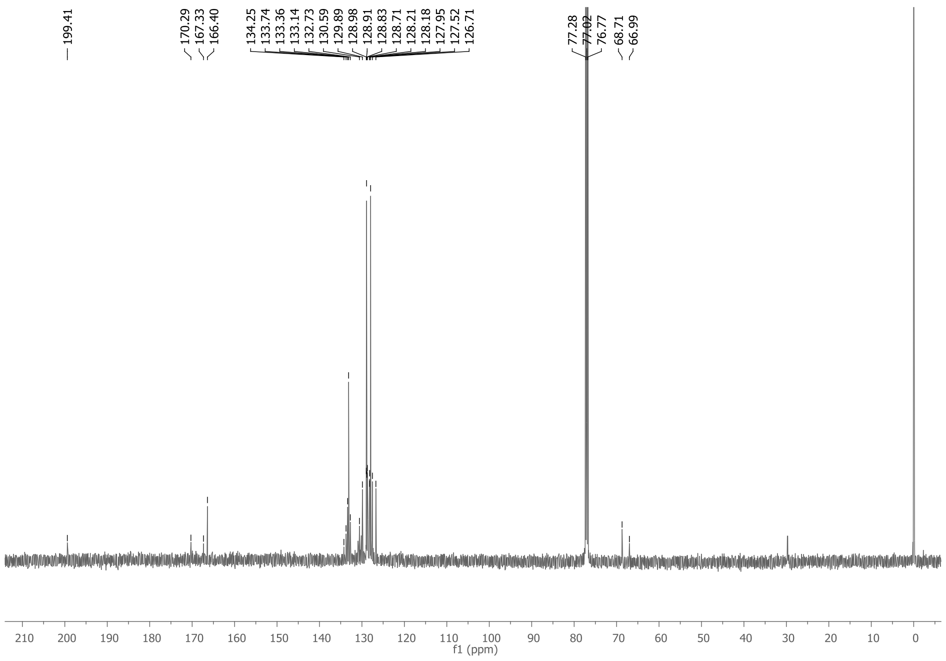

^{1}H NMR of **2j** ($CDCl_3$, 500 MHz).

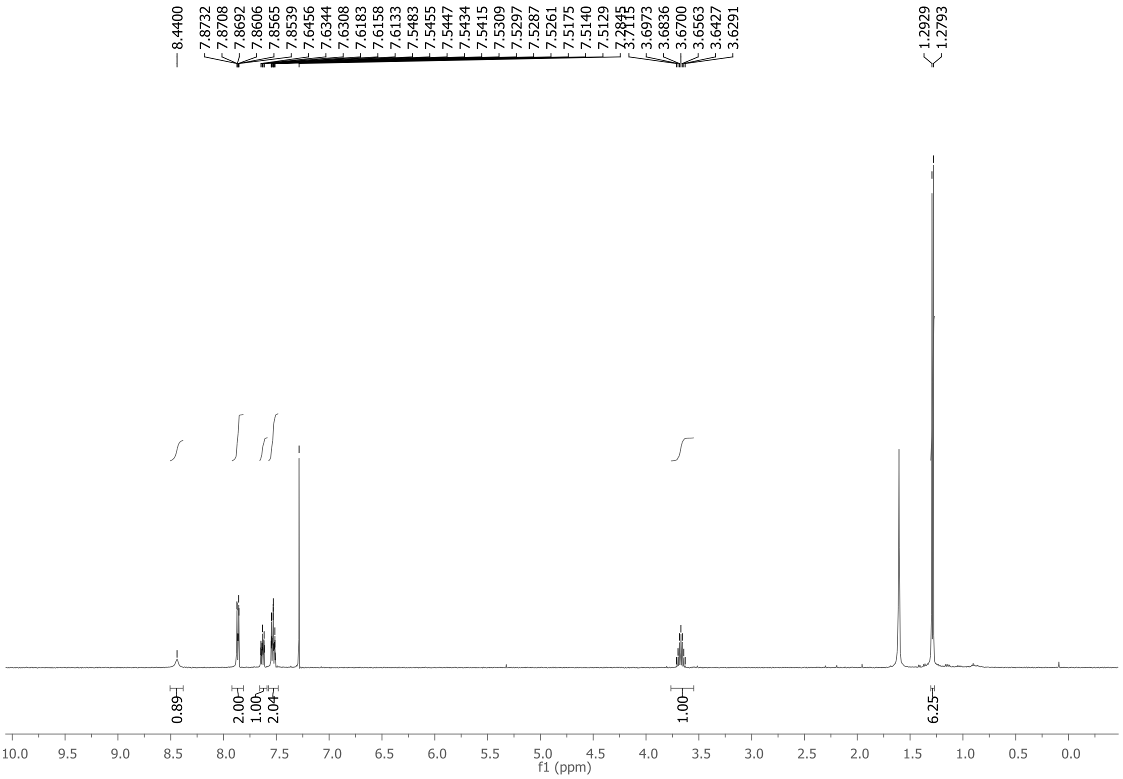

^{13}C NMR of **2j** ($CDCl_3$, 125 MHz).

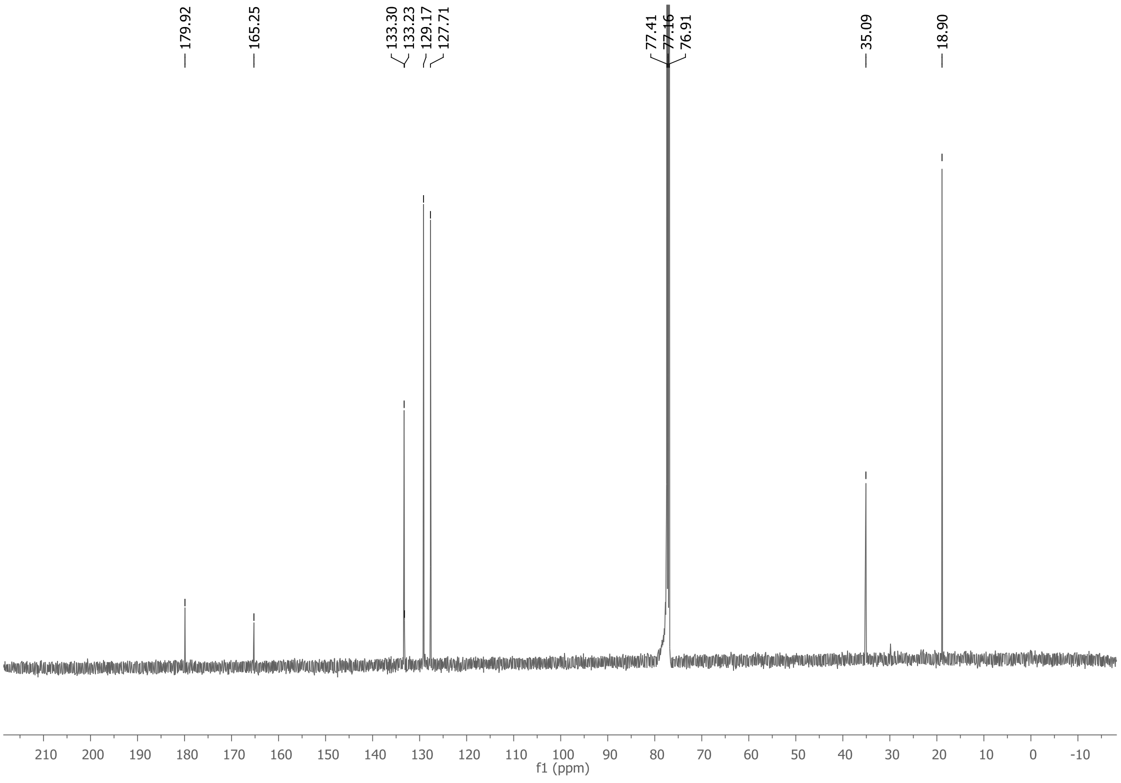

^{1}H NMR of **2k** ($CDCl_3$, 500 MHz).

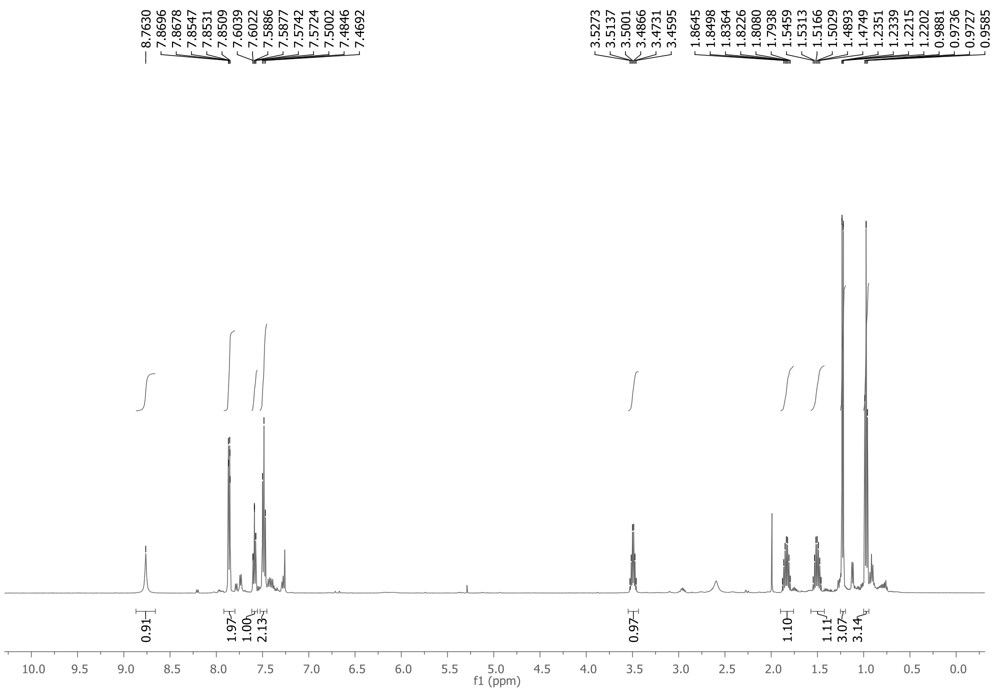

^{13}C NMR of **2k** ($CDCl_3$, 125 MHz).

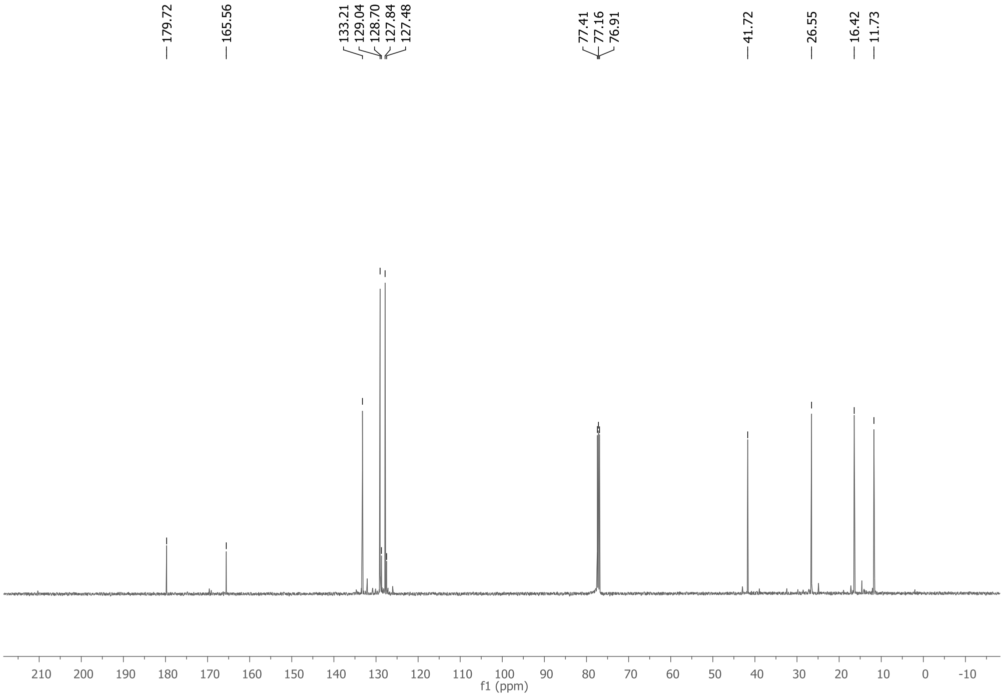

DEPT135 of **2k** ($CDCl_3$, 125 MHz).

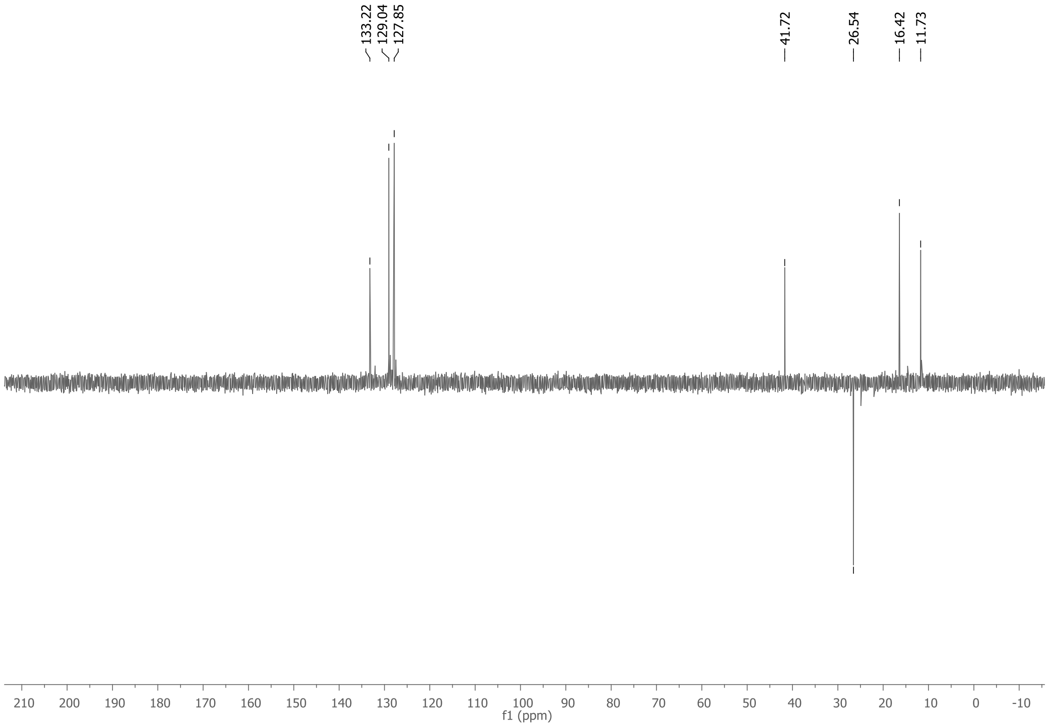

3- ^{1}H and ^{13}C NMR spectra of product 6:

^{1}H NMR of **6** ($CDCl_3$, 500 MHz).

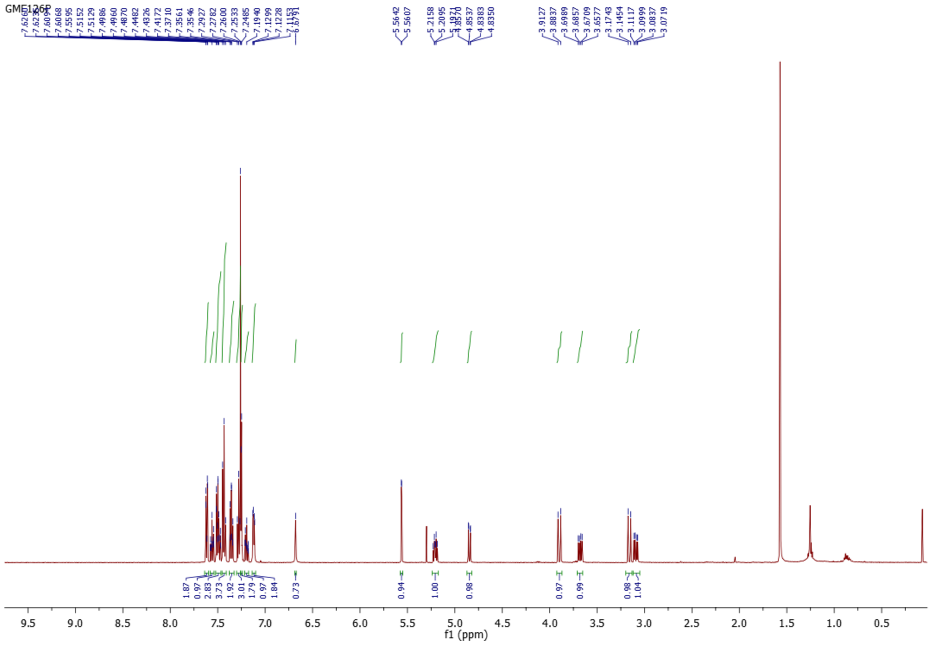

^{13}C NMR of **6** ($CDCl_3$, 125 MHz).

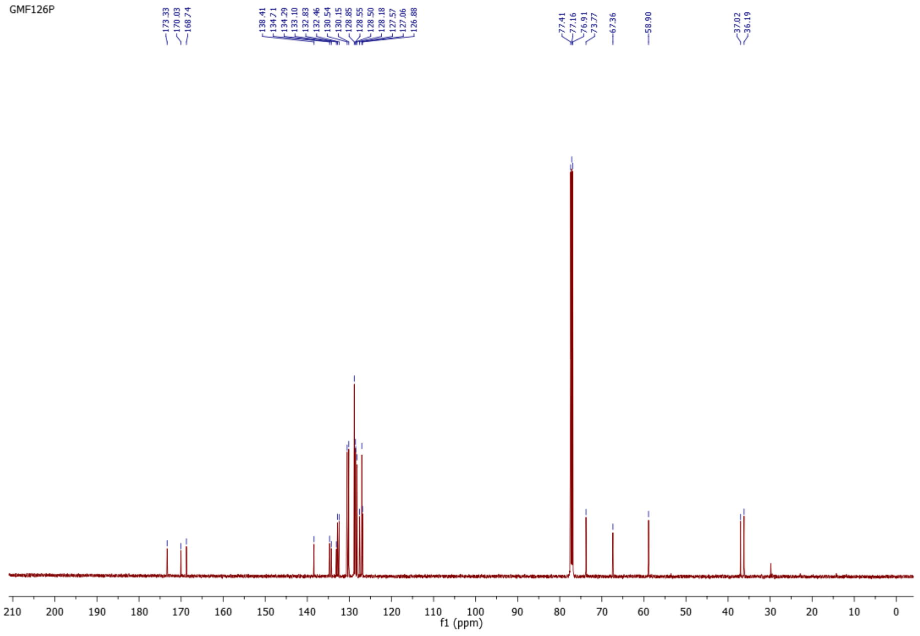

DEPT135 of **6** ($CDCl_3$, 125 MHz).

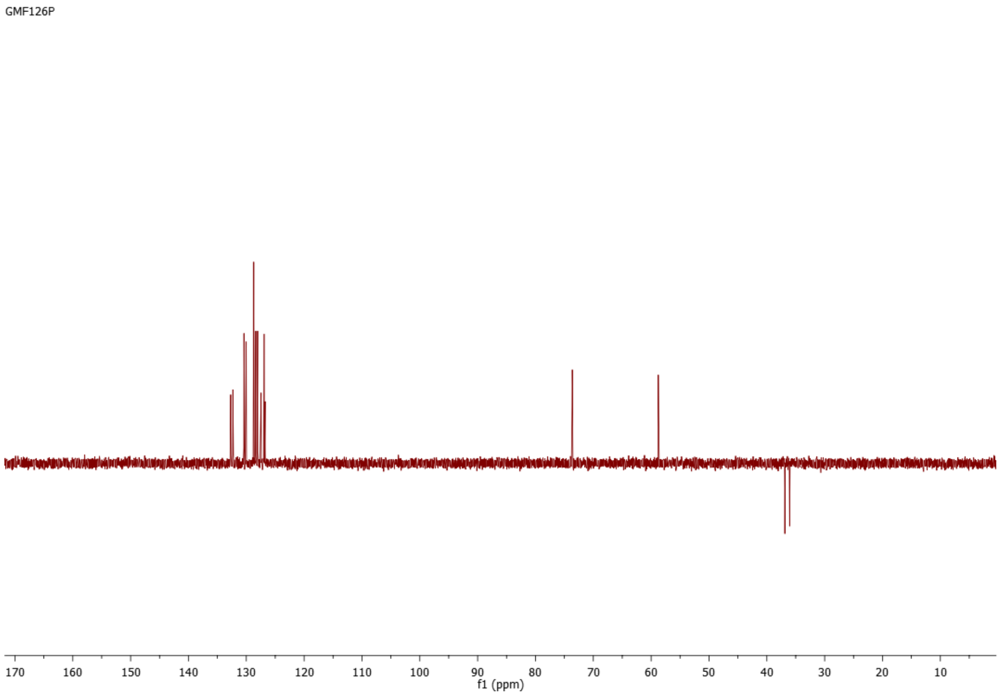

HSQC of **6**

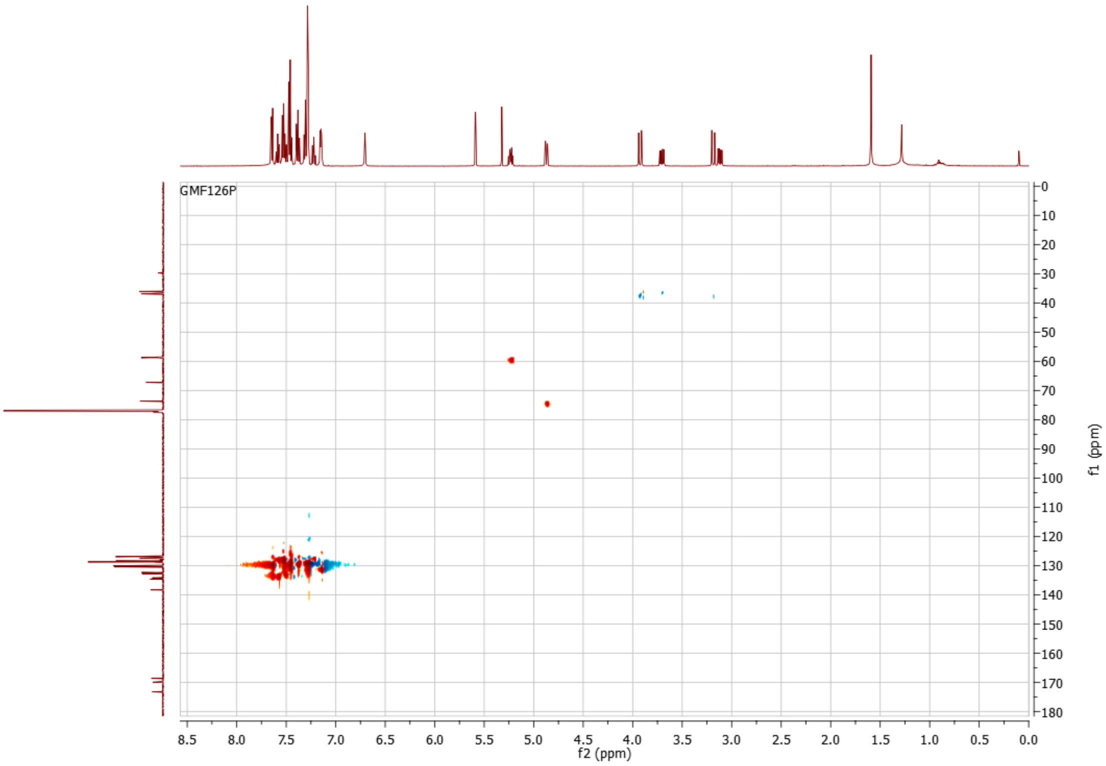

HMBC of **6**

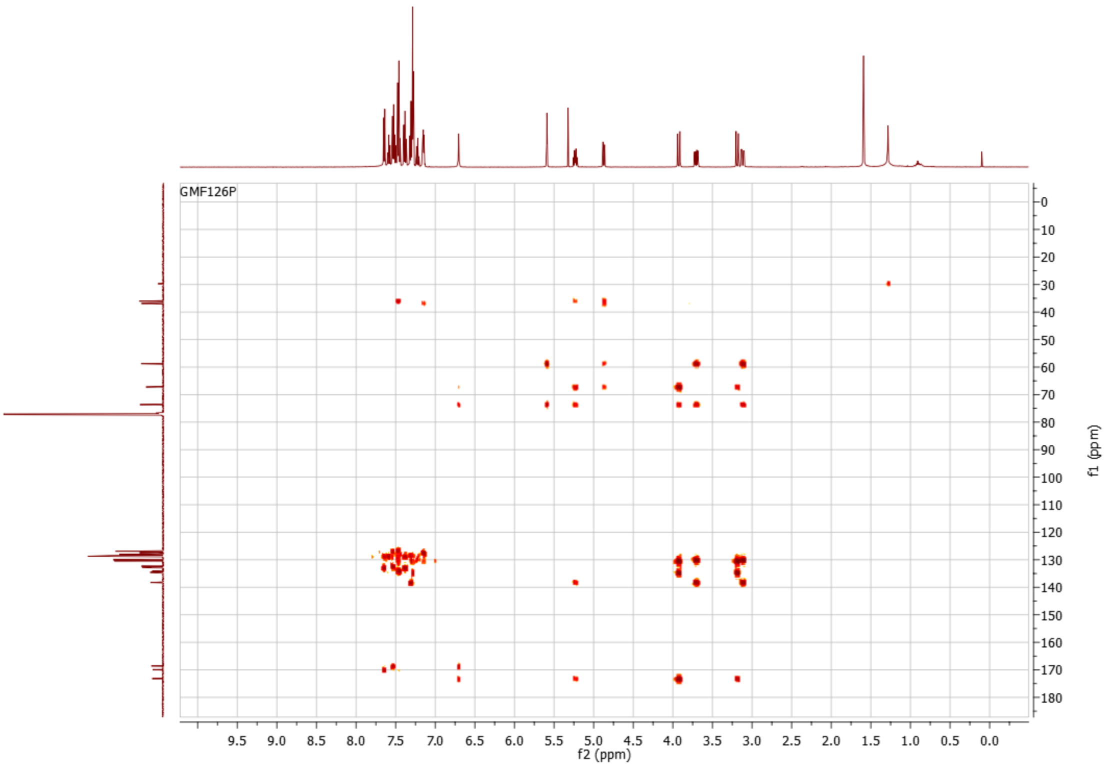

NOESY of **6**

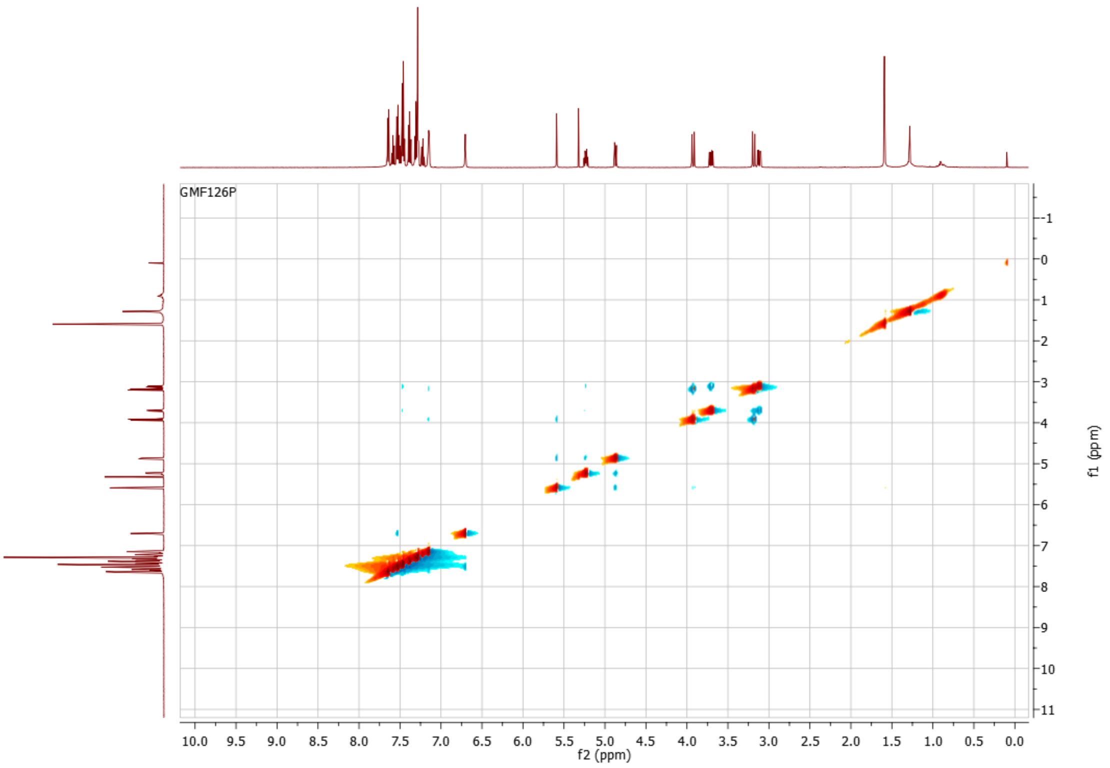

4- Crude NMR data employed for dr calculation

Crude NMR data of compound **2a**

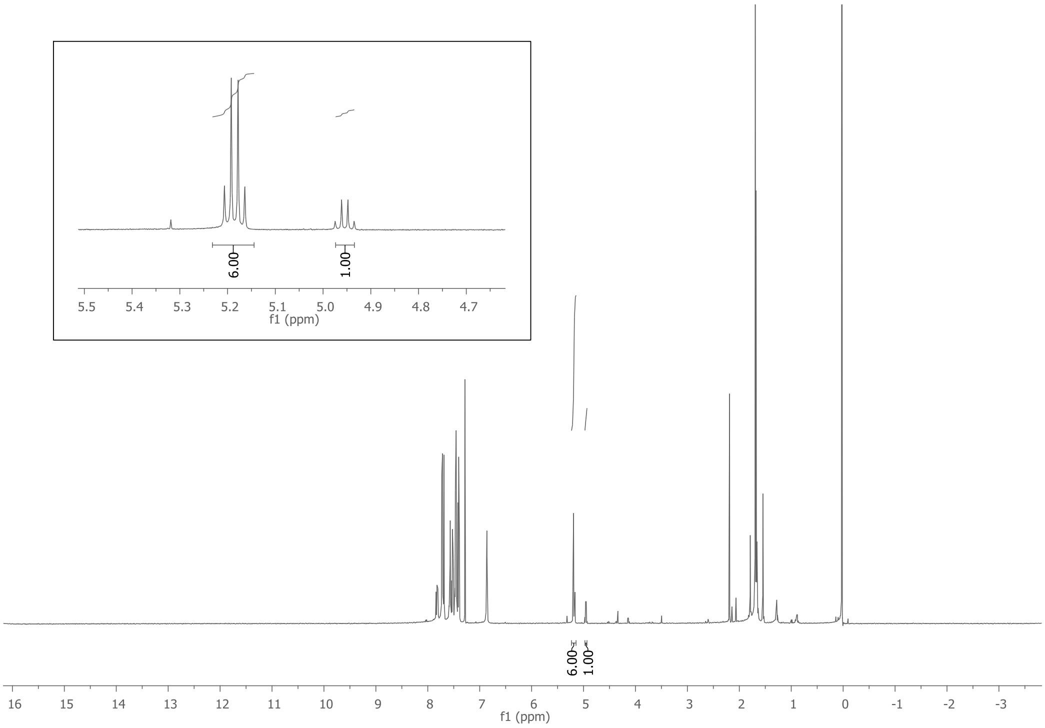

Crude NMR data of compound **2b**

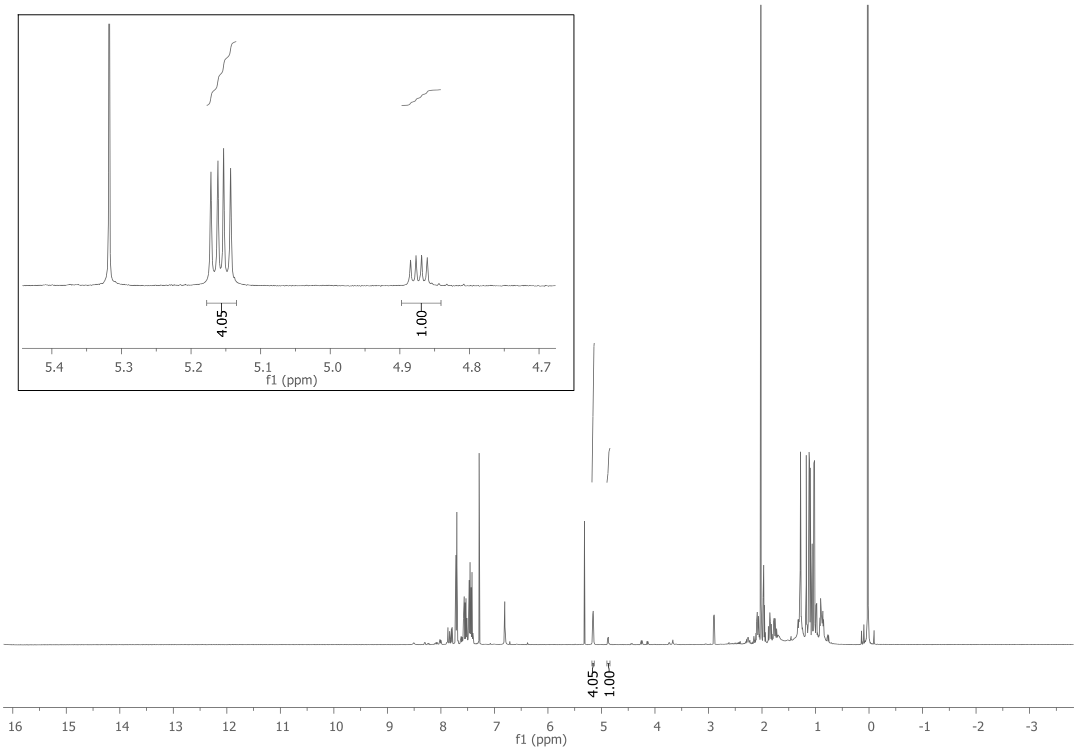

Crude NMR data of compound **2c**

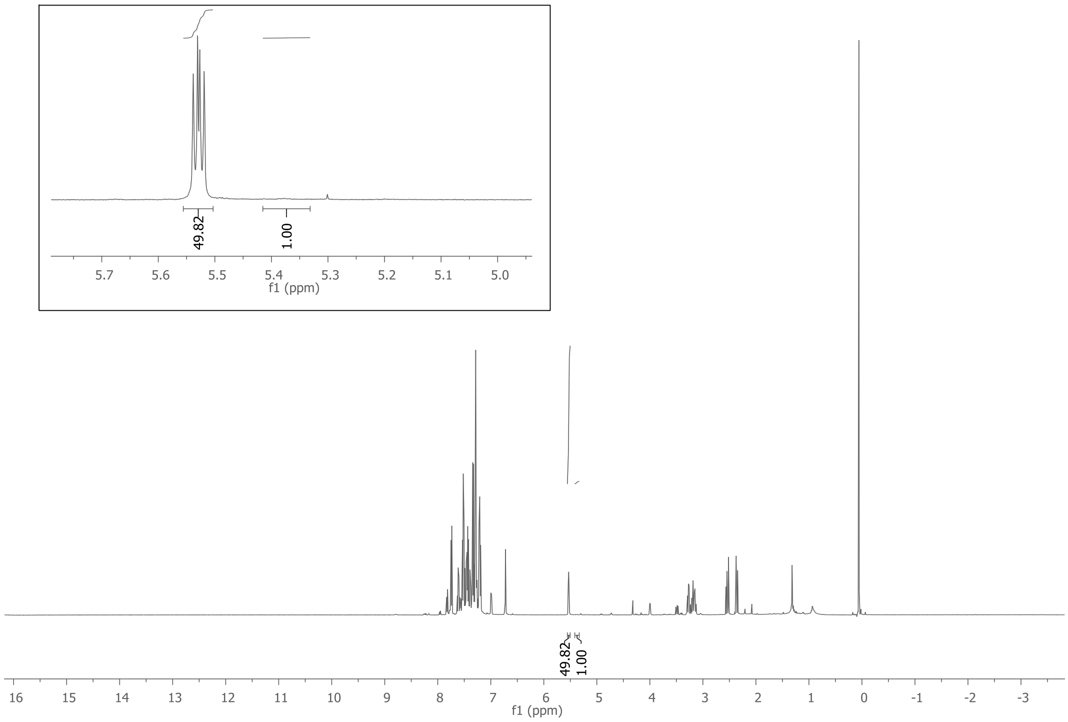

Crude NMR data of compound **2d**

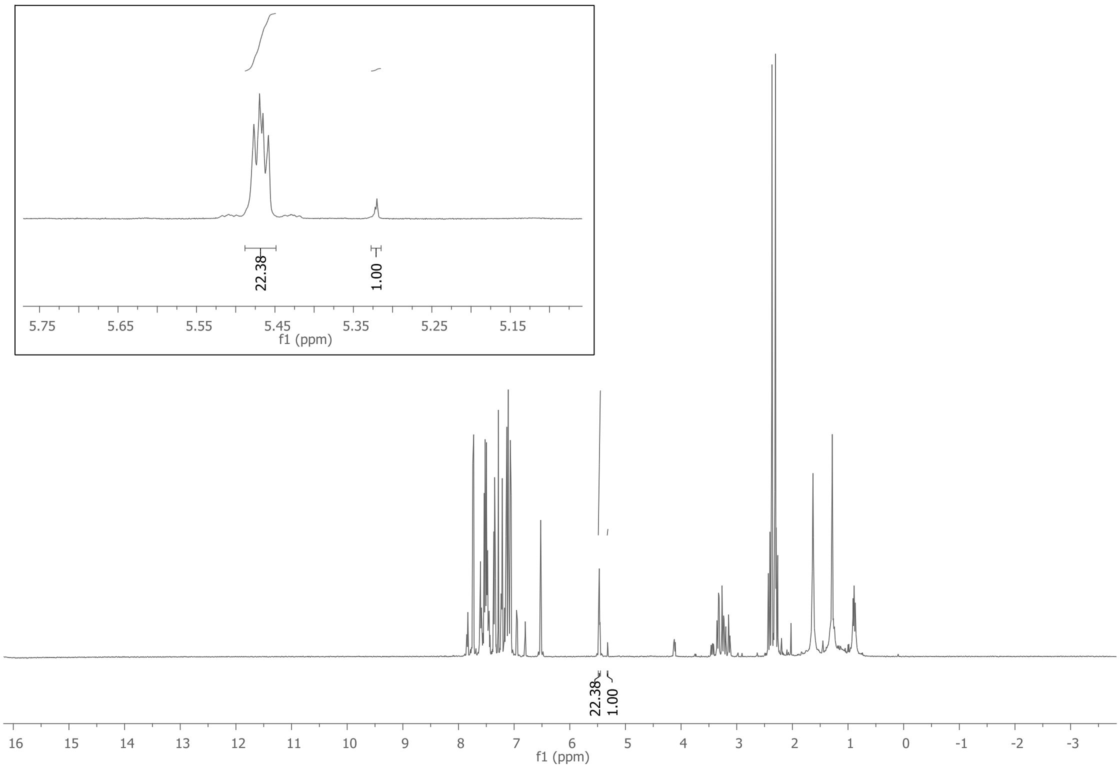

Crude NMR data of compound **2e**

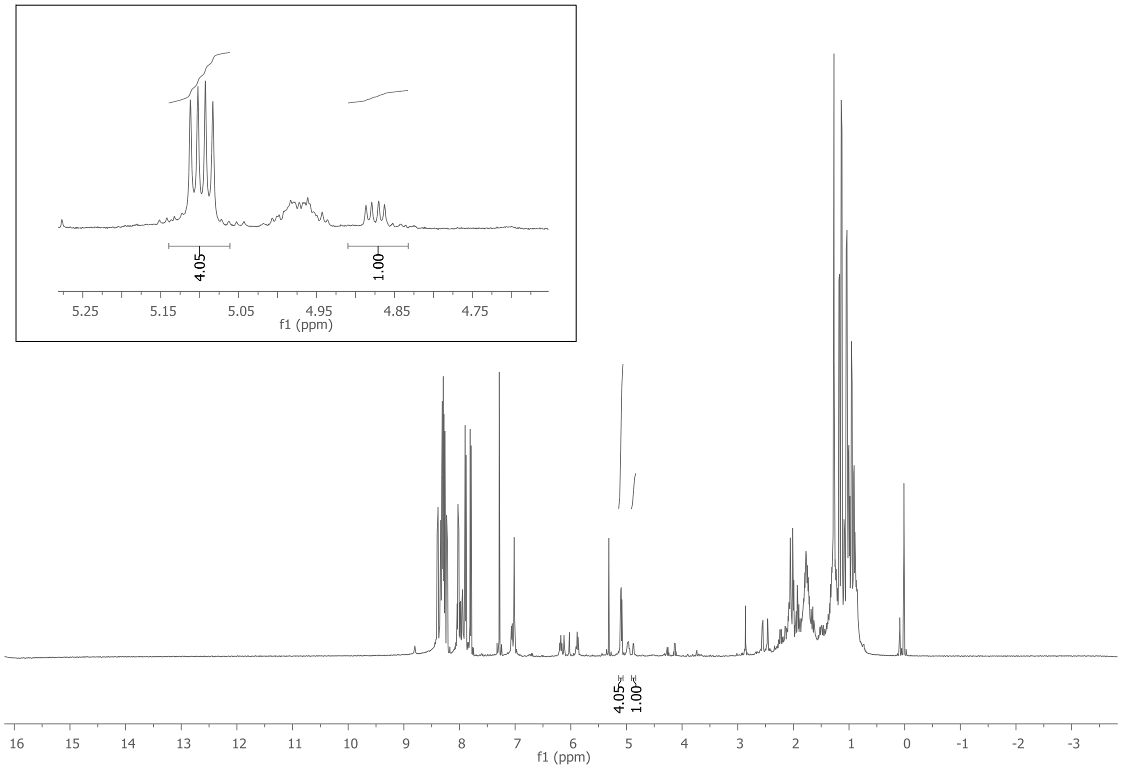

Crude NMR data of compound **2f**

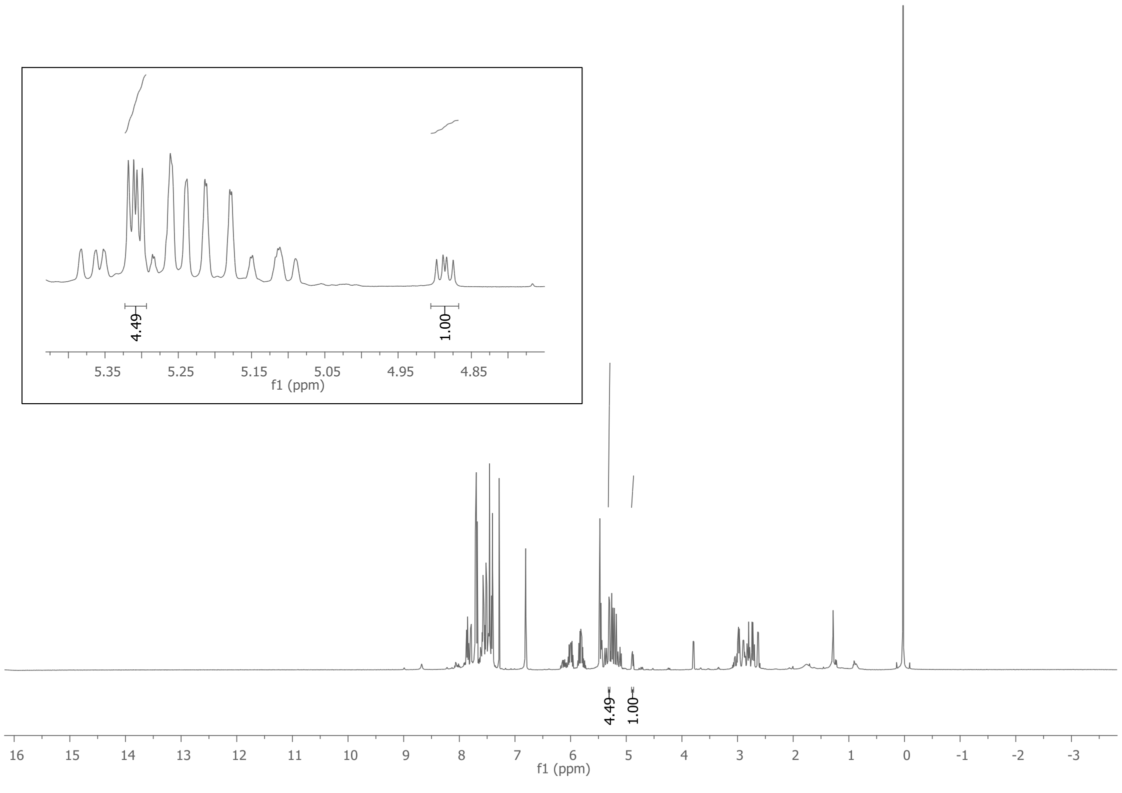

Crude NMR data of compound **2g**

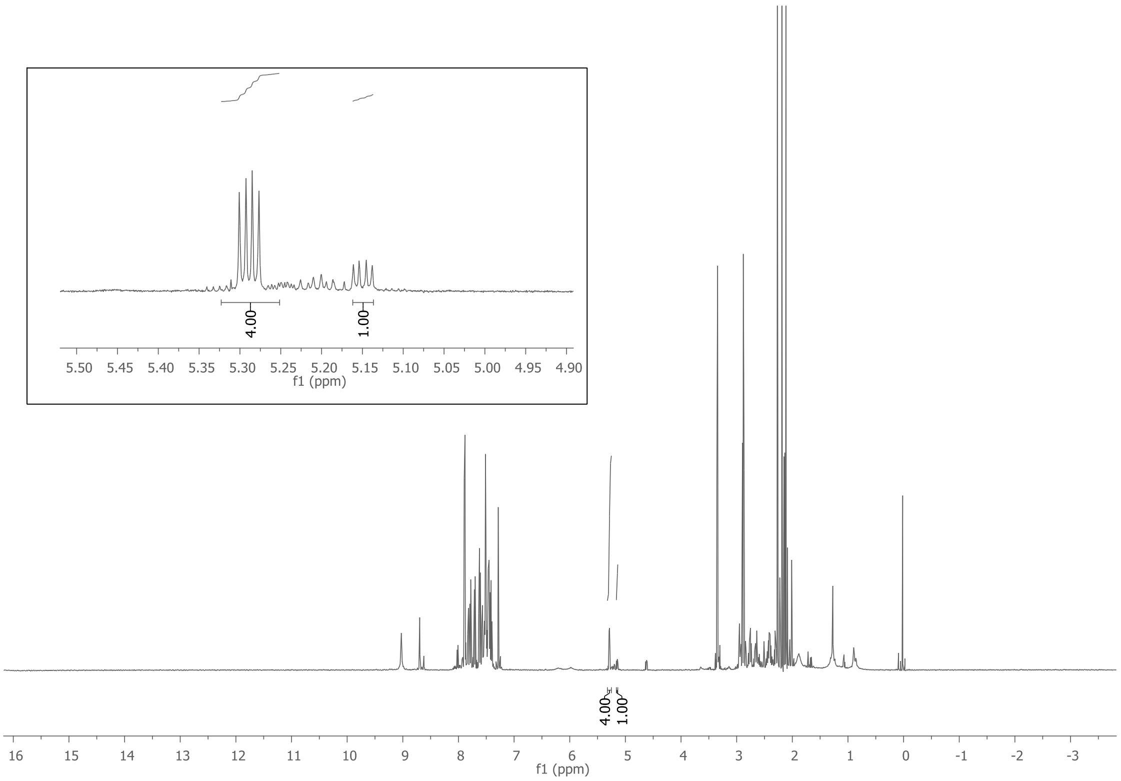

Crude NMR data of compound **2h**

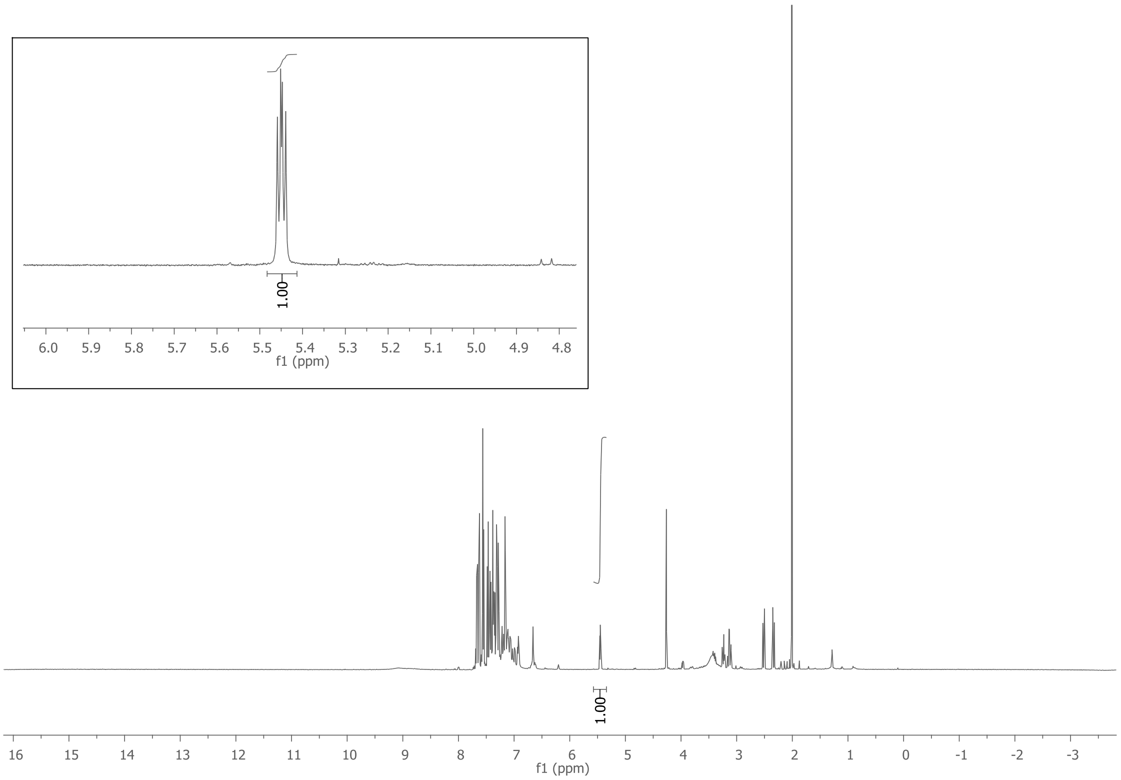

Crude NMR data of compound **2i**

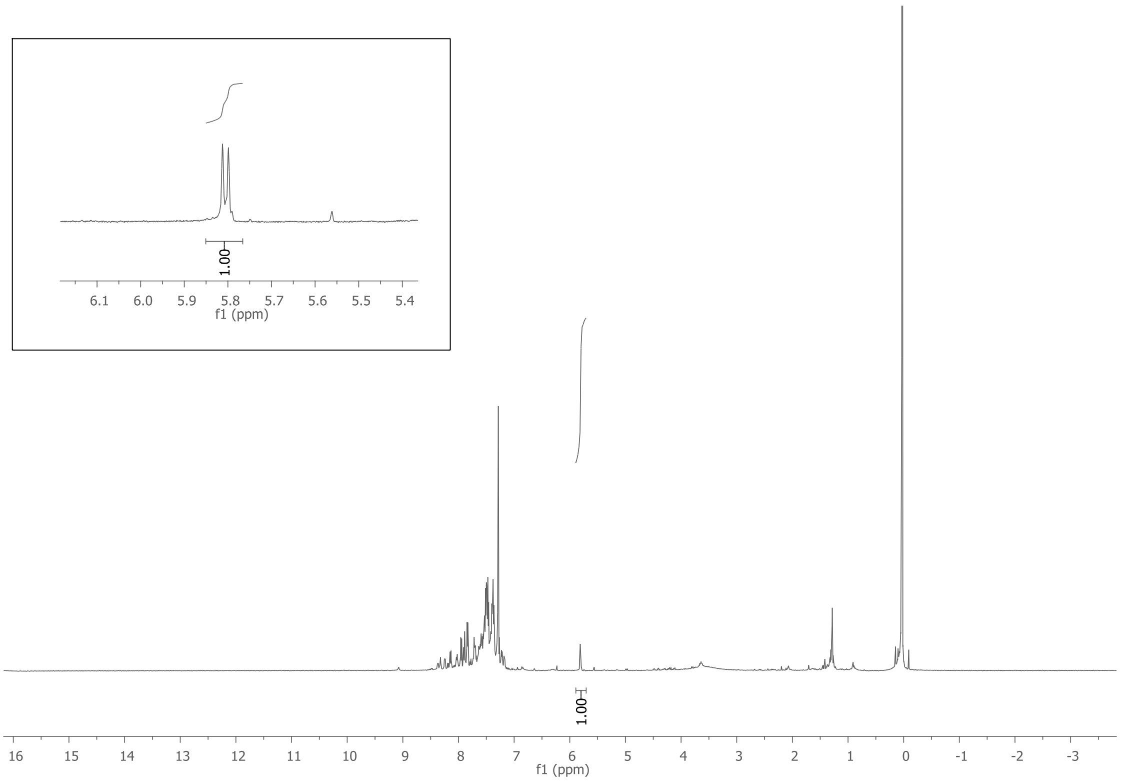

5- Mechanism and kinetic experiments by NMR monitoring:

Reactions were carried out using NMR technique on a 500 MHz spectrometer. Solutions of azlactone **1a** (10.5 mg; 0.06 mmol) and sodium trichloroacetate salt (30 mol %; 1.6 mg) were prepared individually in deuterated acetonitrile (1 mL). The solutions were added to a NMR tube and the reaction was maintained at 25 ºC and monitored by a single pulse ^{1}H NMR for 15 min. A plot of $1/\sqrt{[A]}$ vs time showed that the reaction exhibited a 3/2 order behavior in azlactone such as was suggested by Mazurkiewick et al. [4]. Results based on average of three runs.

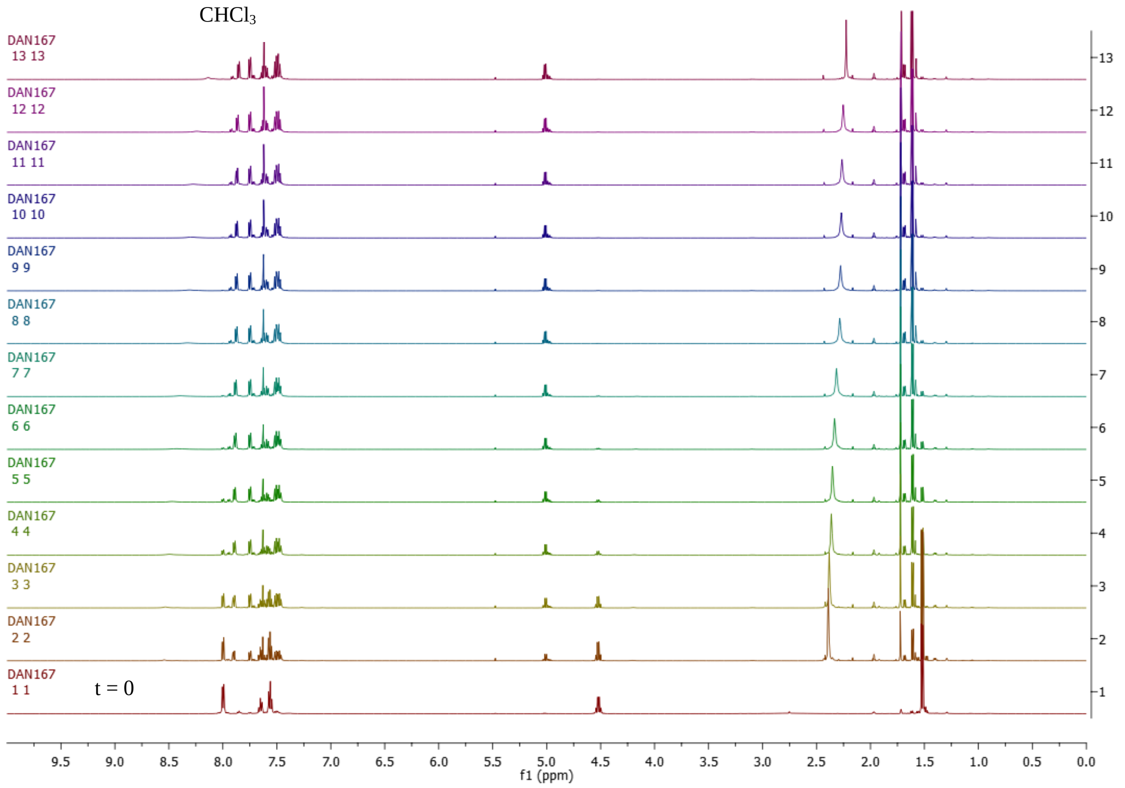

^{1}H NMR spectra copies which show the formation of $CHCl_3$. The corresponding trichloroacetate salts are freshly prepared, which we believe a little bit of water remains:

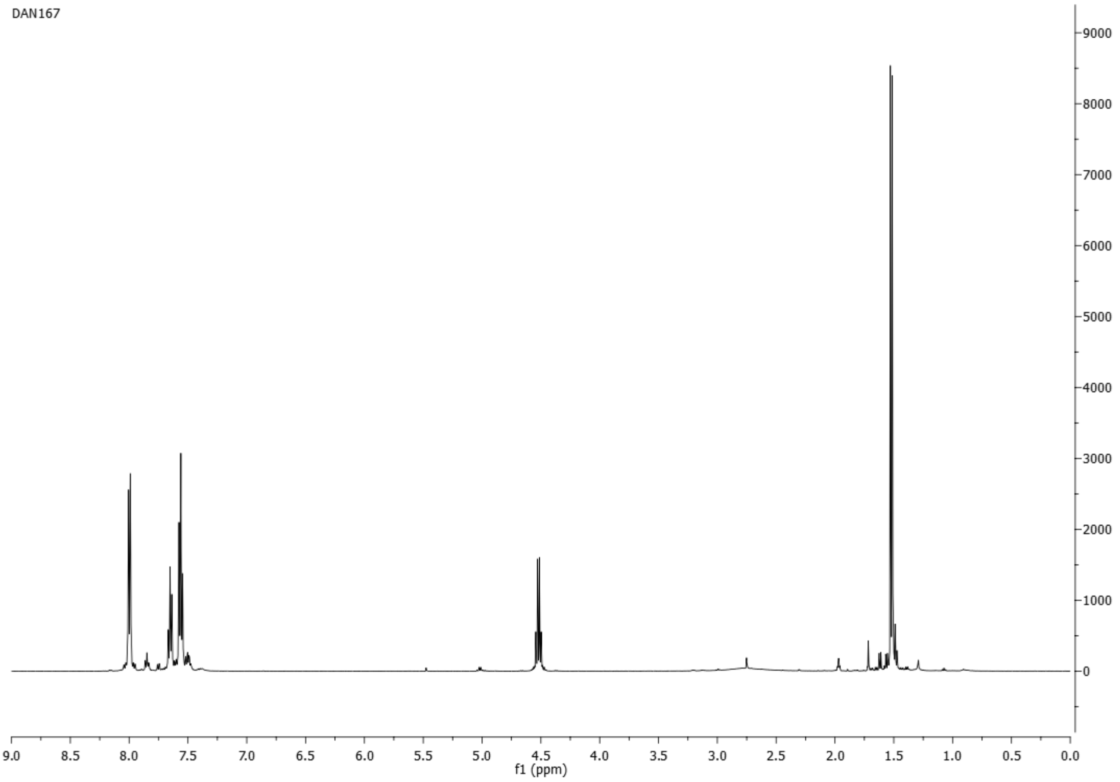
DAN167
f1 (ppm)

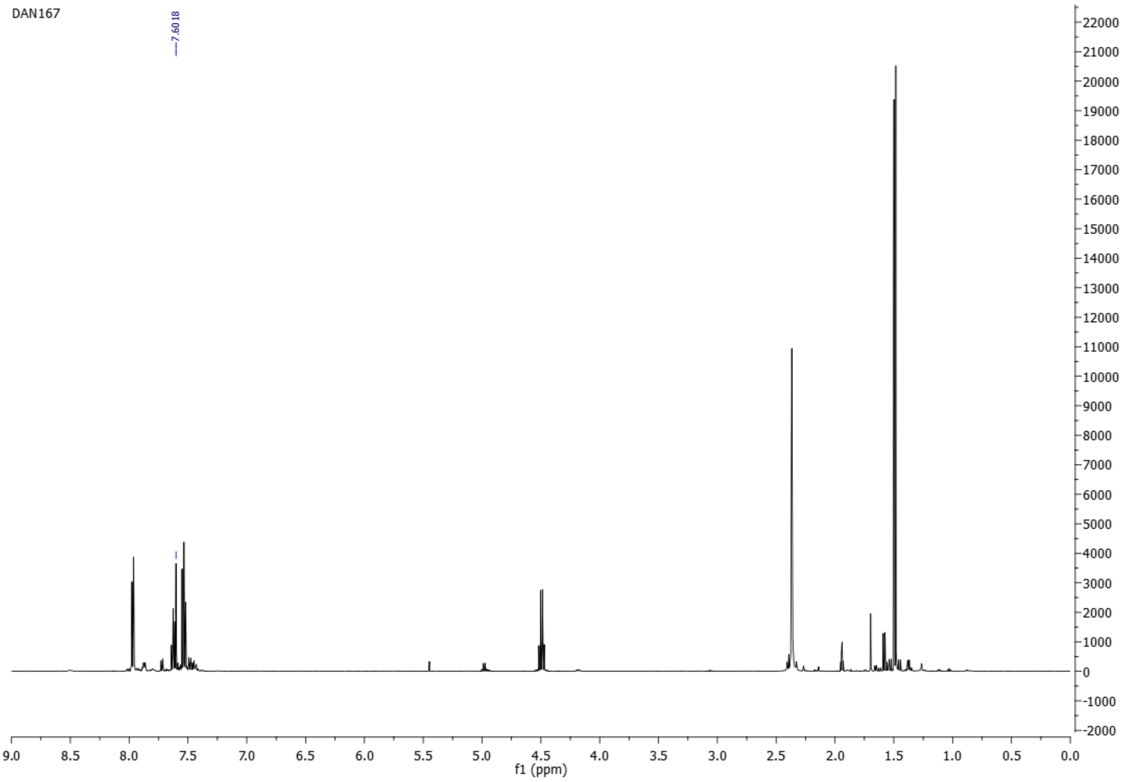
DAN167
7.60 18
f1 (ppm)

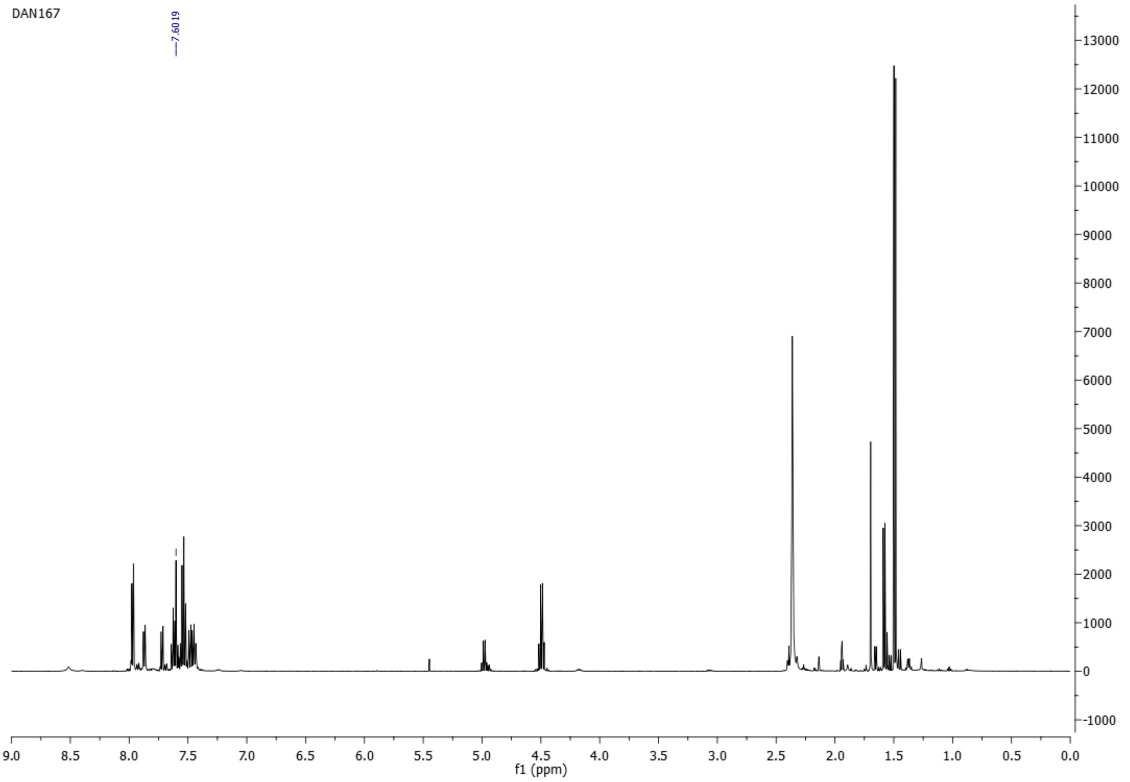
DAN167
7.6019
f1 (ppm)

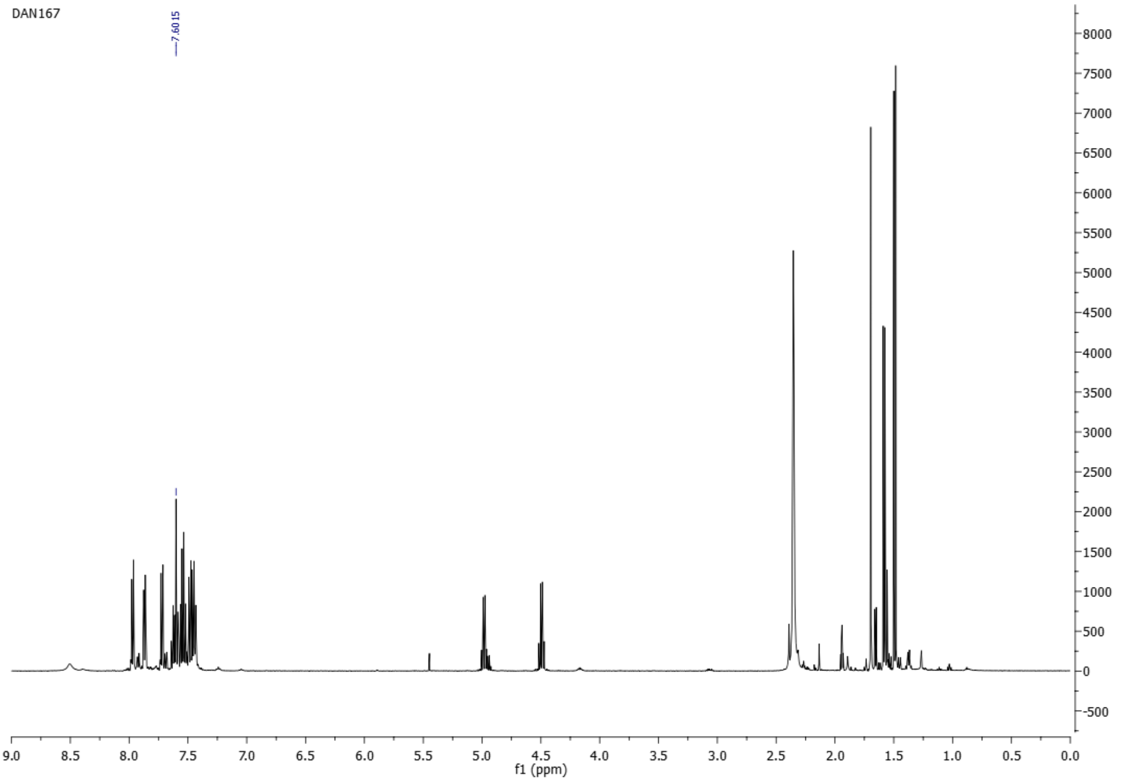
DAN167
7.6015
f1 (ppm)

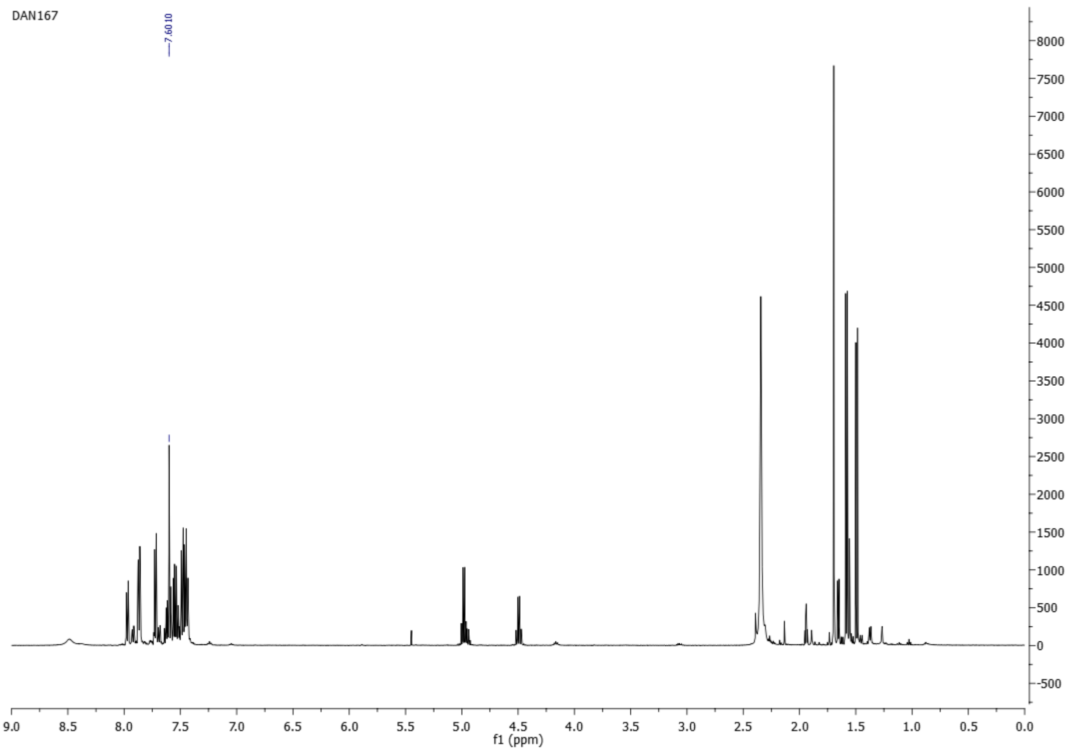
DAN167
7.6010
f1 (ppm)

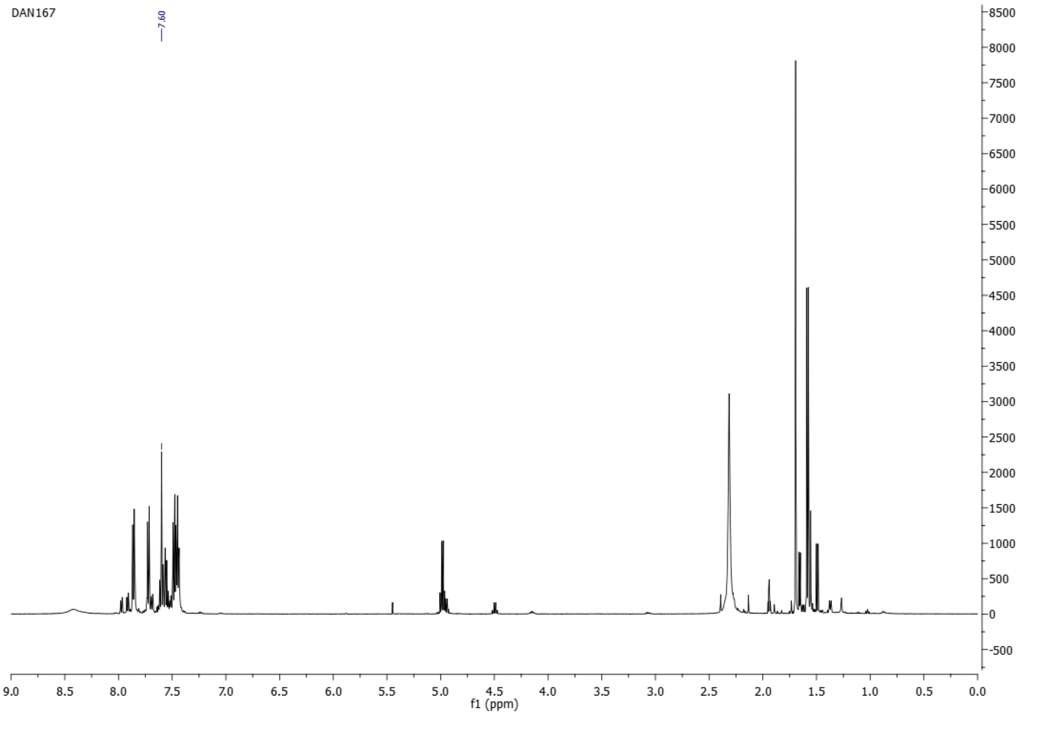
DAN167
7.60
f1 (ppm)

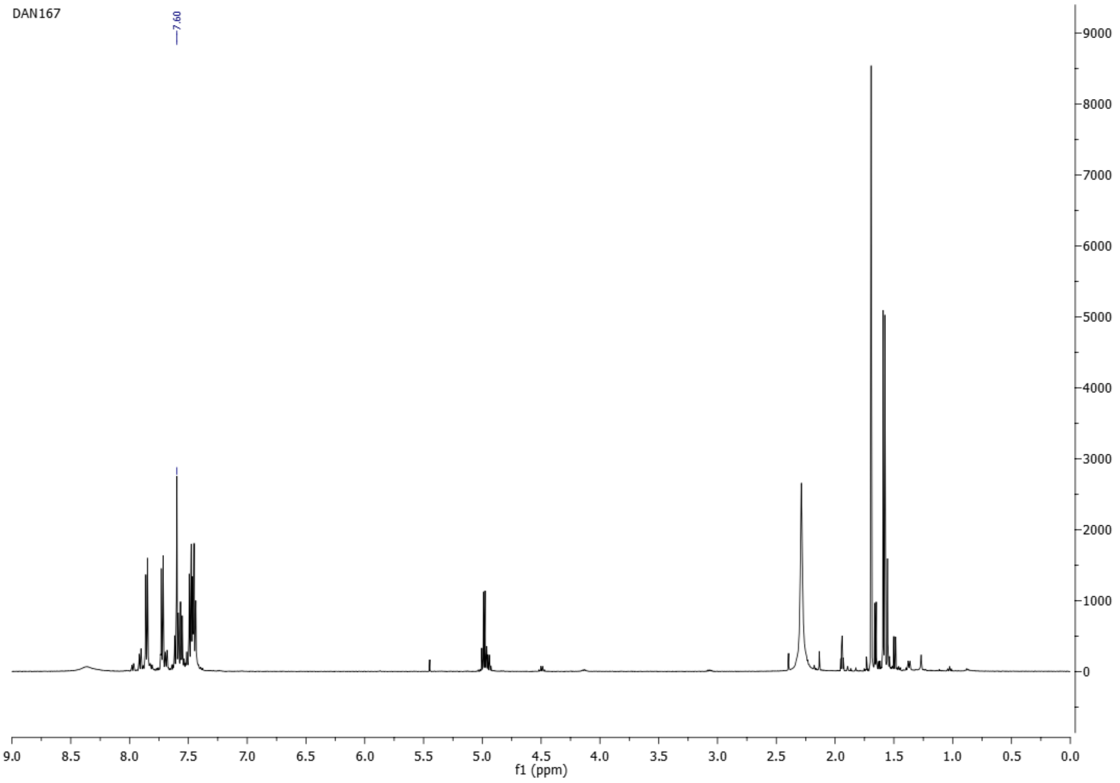
DAN167
7.60
9000
8000
7000
6000
5000
4000
3000
2000
1000
0
9.0 8.5 8.0 7.5 7.0 6.5 6.0 5.5 5.0 4.5 4.0 3.5 3.0 2.5 2.0 1.5 1.0 0.5 0.0
f1 (ppm)

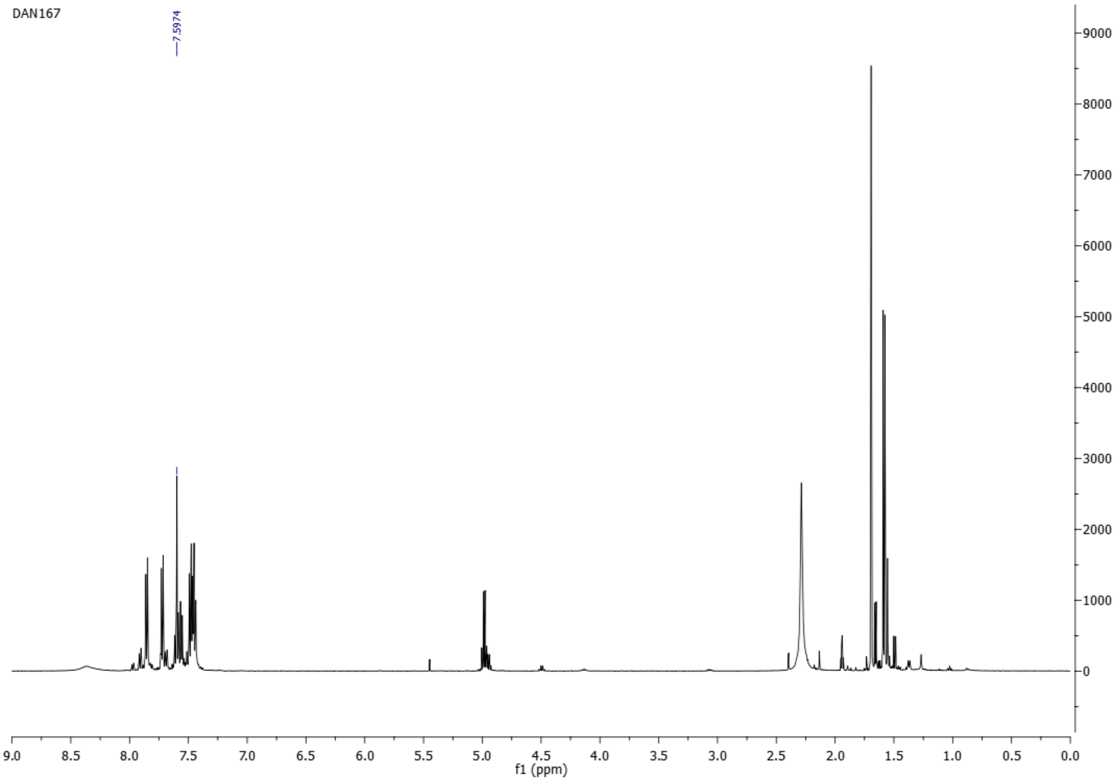
DAN167
7.5974
9000
8000
7000
6000
5000
4000
3000
2000
1000
0
9.0 8.5 8.0 7.5 7.0 6.5 6.0 5.5 5.0 4.5 4.0 3.5 3.0 2.5 2.0 1.5 1.0 0.5 0.0
f1 (ppm)

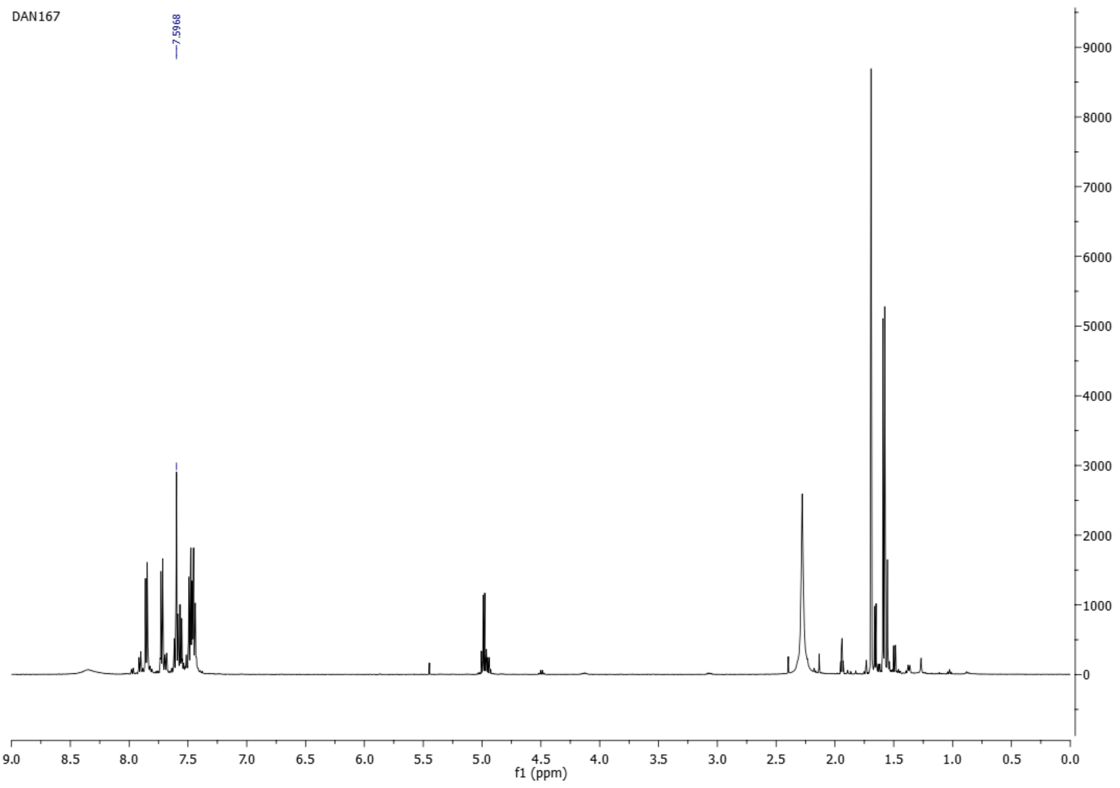
DAN167
7.5968
f1 (ppm)

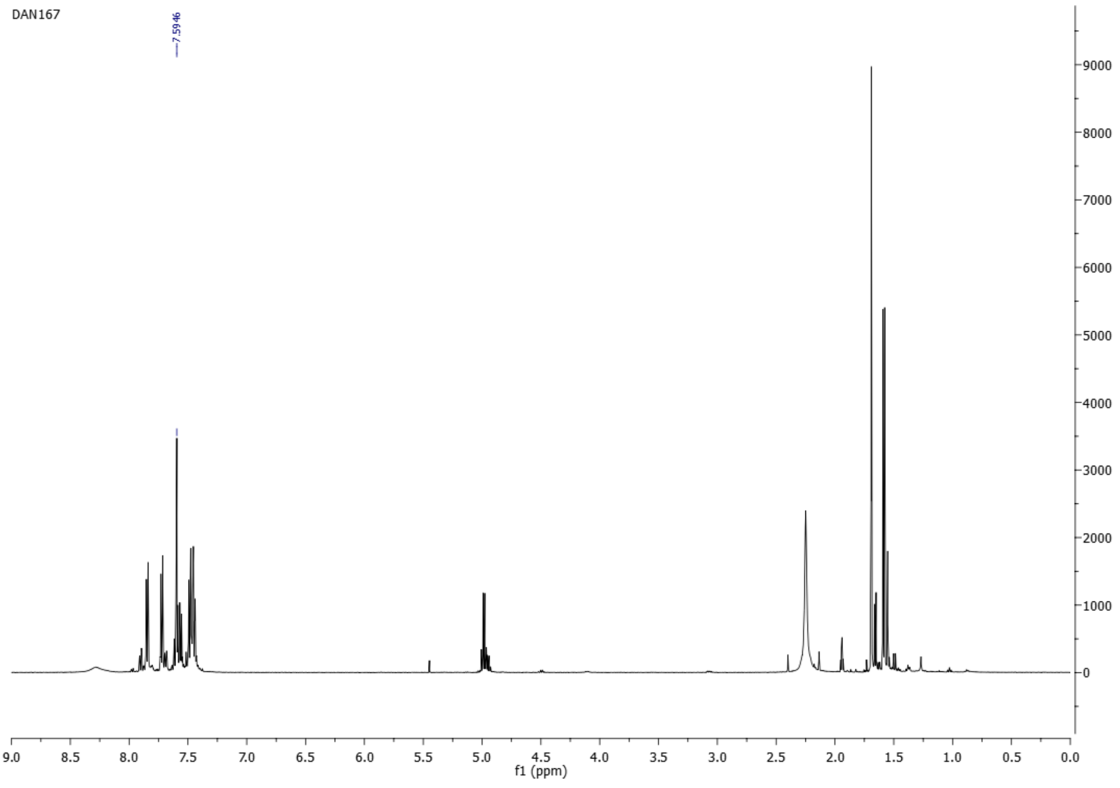
DAN167
7.5946
f1 (ppm)

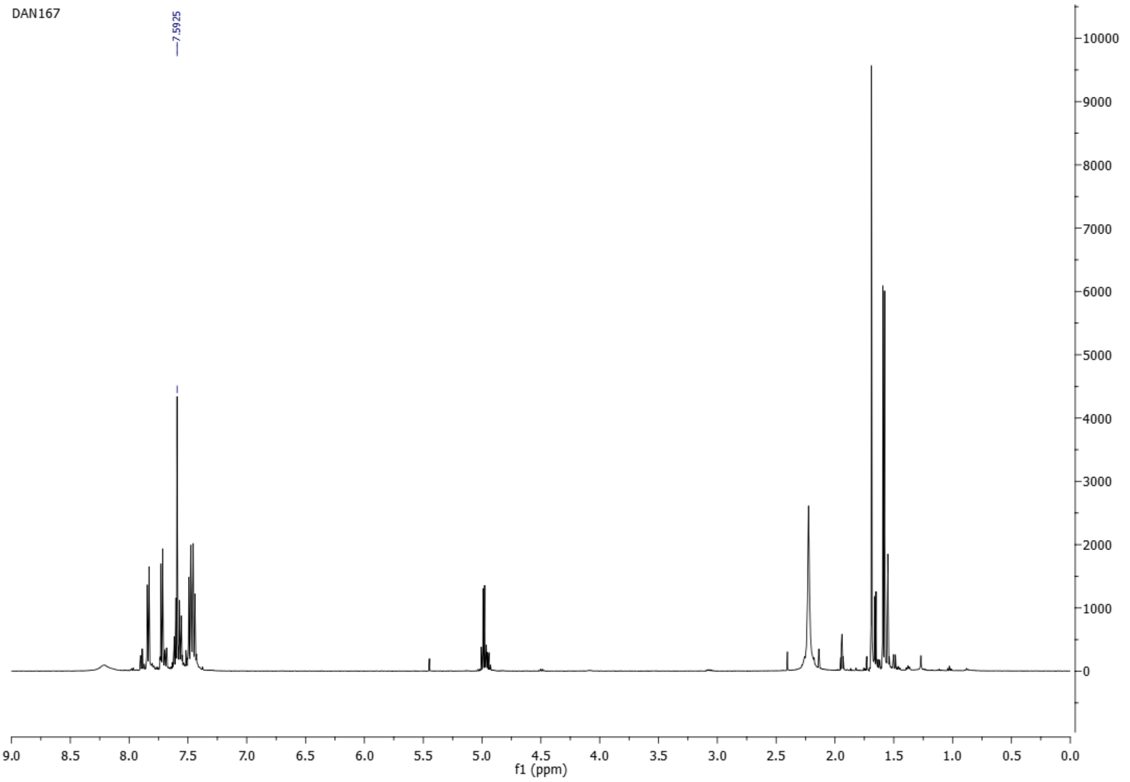

DAN167
7.5925
10000
9000
8000
7000
6000
5000
4000
3000
2000
1000
0
9.0
8.5
8.0
7.5
7.0
6.5
6.0
5.5
5.0
4.5
4.0
3.5
3.0
2.5
2.0
1.5
1.0
0.5
0.0
f1 (ppm)

Kinetic graphics:

A plot of $1/\sqrt{[A]}$ vs time showed that the reaction exhibited a 3/2 order behavior in azlactone such as was suggested by Mazurkiewick et al. [4]. Results based on average of three runs.

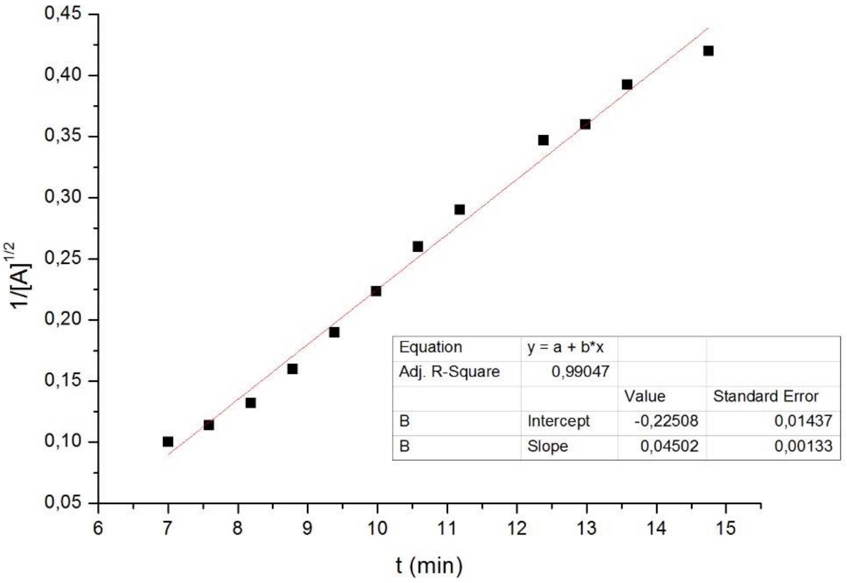

$1/[A]^{1/2}$= (0.0450±0.0013) – (0.225±0.014)t

R^2= 0.99047

Graphic using initial rates technique. Results based on average of three runs:

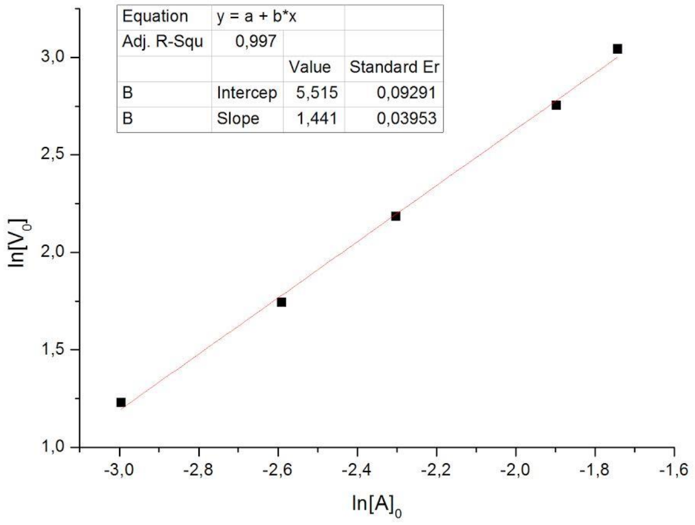

- Demonstration of reaction order equations for the proposed mechanism:

To obtain the expected reaction order based on the proposed mechanism, one may assume, to simplify notation, in the proposed mechanism in Figure 3 of the main text:

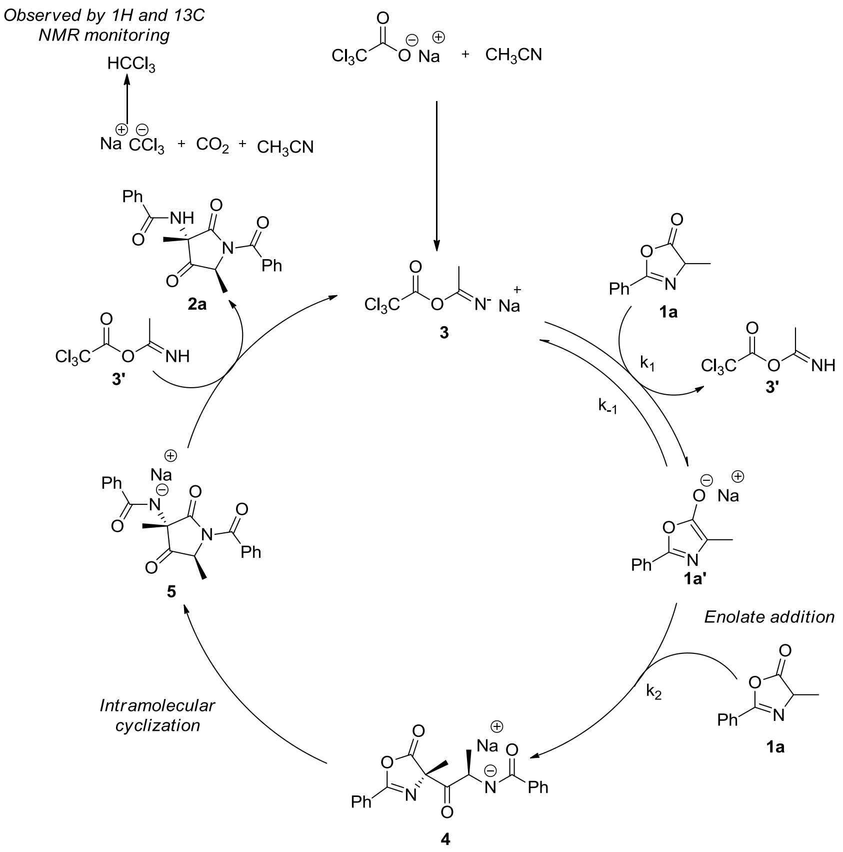

1a is termed A, **1a'** is termed A$^-$, **3** is termed B, and **3'** is termed BH. In such terms, the time dependence of [A$^-$] may be written:

$$\frac{d[A^-]}{dt} = k_1[A][B] - k_{-1}[A^-][BH] - k_2[A][A^-] \qquad (S1)$$

Considering the equilibrium for the formation of A$^-$ in the first step is quickly achieved, so that $k_{-1}[A][B] \gg k_2[A][A^-]$, the second step may be considered the rate determining step, and one may write down the approximation:

$$-\frac{d[A^-]}{dt} = k_2[A][A^-] \qquad (S2)$$

Considering A$^-$ as a steady-state, so that only a small concentration of the anion will be present (due to the high value of k_{-1}), then $d[A^-]/dt = 0$, so that:

$$k_1[A][B] = k_{-1}[A^-][BH] + k_2[A][A^-] \qquad (S3)$$

Additionally, because $k_{-1}[A][B] \gg k_2[A][A^-]$, then $k_2[A][A^-]$ may be considered negligibly small; and, also due to the same relation above, it may be assumed that only a small fraction of the basis B will exist in the protonated state BH, so that [*B*]=[*B*]$_0$, where [*B*]$_0$ is the initial concentration of the basis added to the reaction vessel, and also that $[A^-] \cong [BH]$. Applying the above considerations, eq. (S3) becomes:

$$k_1[A][B]_0 = k_{-1}[A^-]^2$$

Or:

$$[A^-] = \left(\frac{k_1}{k_{-1}}[A][B]_0\right)^{1/2} \qquad (S4)$$

Substituting (S4) in (S2):

$$-\frac{d[A^-]}{dt} = k_2\left(\frac{k_1}{k_{-1}}\right)^{1/2}[A]^{3/2}[B]_0^{1/2} \qquad (S5)$$

Equation (S5) indicates that the reaction order for compound 1a would be 3/2 if the reaction mechanism is correct.

Isolating the terms containing [A-] and t, and integrating in both sides:

$$\frac{1}{[A]^{1/2}} - \frac{1}{[A]_{t=0}{}^{1/2}} = \frac{k_2}{2}\left(\frac{k_1}{k_{-1}}\right)^{1/2}[B]_0^{1/2}\, t \qquad (S6)$$

Or

$$[A]^{-1/2} = [A]_{t=0}{}^{-1/2} + \frac{k_2}{2}\left(\frac{k_1}{k_{-1}}\right)^{1/2}[B]_0^{1/2}\, t \qquad (S7)$$

Setting $[A]_{t=0}{}^{-1/2} \equiv a$ and $\frac{k_2}{2}\left(\frac{k_1}{k_{-1}}\right)^{1/2}[B]_0^{1/2} \equiv b$ and rearranging, one may get equation (1) in the main text.

Finally, from equation S6 one may state that a plot of $[A]^{-1/2}$ v. time should result in a linear relationship if the reaction order was actually $^3/_2$ for compound 1a. It was taken into consideration the possibility of other reaction orders resulting in better description of the experimental data of [A] versus time, and fits to different order using the corresponding integrated rate laws were performed, as presented in Table S1.

Table S1. Reaction order most probable possibilities, fitted equation to data and the obtained correlation factor (R^2).

Reaction order	Fitted equation	R^2
**1**	$\ln([A]/[A]_0)$ v. t	0.94154
**2**	$1/[A]$ v. t	0.97506
**$^3/_2$**	$1/[A]^{1/2}$ v. t	0.99047

One may observe in Table S1 that the integrated rate law that results in the best fit for the experimental data, measured by the R2, is the one that corresponds to the $^3/_2$ reaction order (in comparison to the expected relations for other reaction orders). The adjusted data for the $^3/_2$ reaction order is presented in Figure 4 of the main text.

6. Reaction reversibility evaluation

Reversibility study was carried out by adding the purified product **2h** in the optimized reaction conditions (30 mol % of NaTCA, acetonitrile as solvent, room temperature for 1h). The crude reaction mixture was diluted with CH_2Cl_2 and washed with water. The organic layer was dried over anhydrous Na_2SO_4, filtered off and concentrated under reduced pressure. An aliquot was dissolved in $CDCl_3$ and analyzed by ^1H NMR in order to evaluate the corresponding d.r. No reaction takes place (the reaction isn't reversible) and no differences were detected in the diastereomeric ratio (only one diastereomer could be detected, the same that the original product **2h**).

Reaction reversibility study.

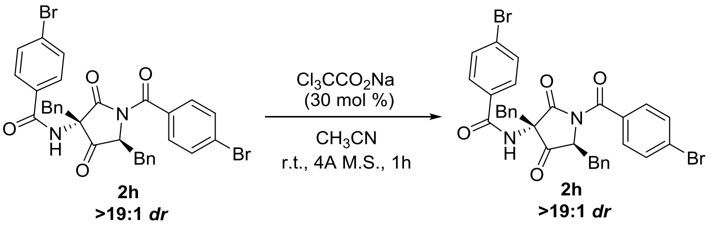

NMR data of product **2h** after the reversibility test.

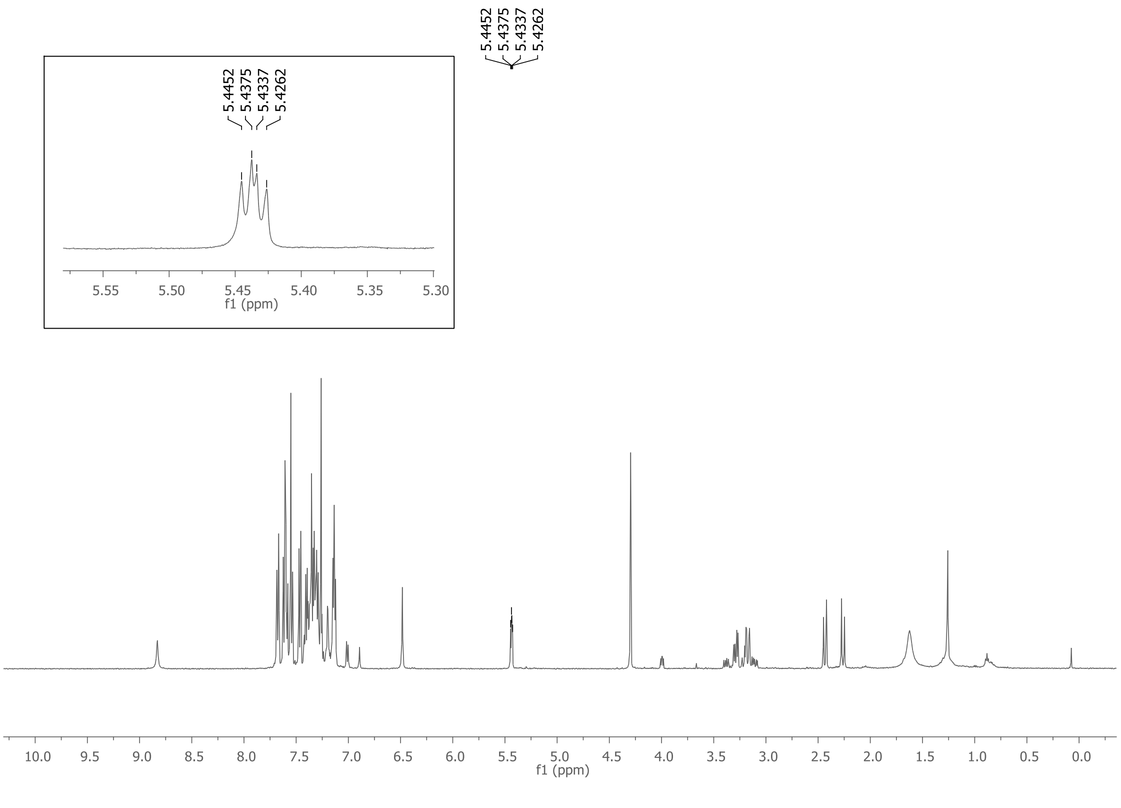

7- X-ray data

Figure 1. ORTEP representation of asymmetric unit of **2a** crystal structure.

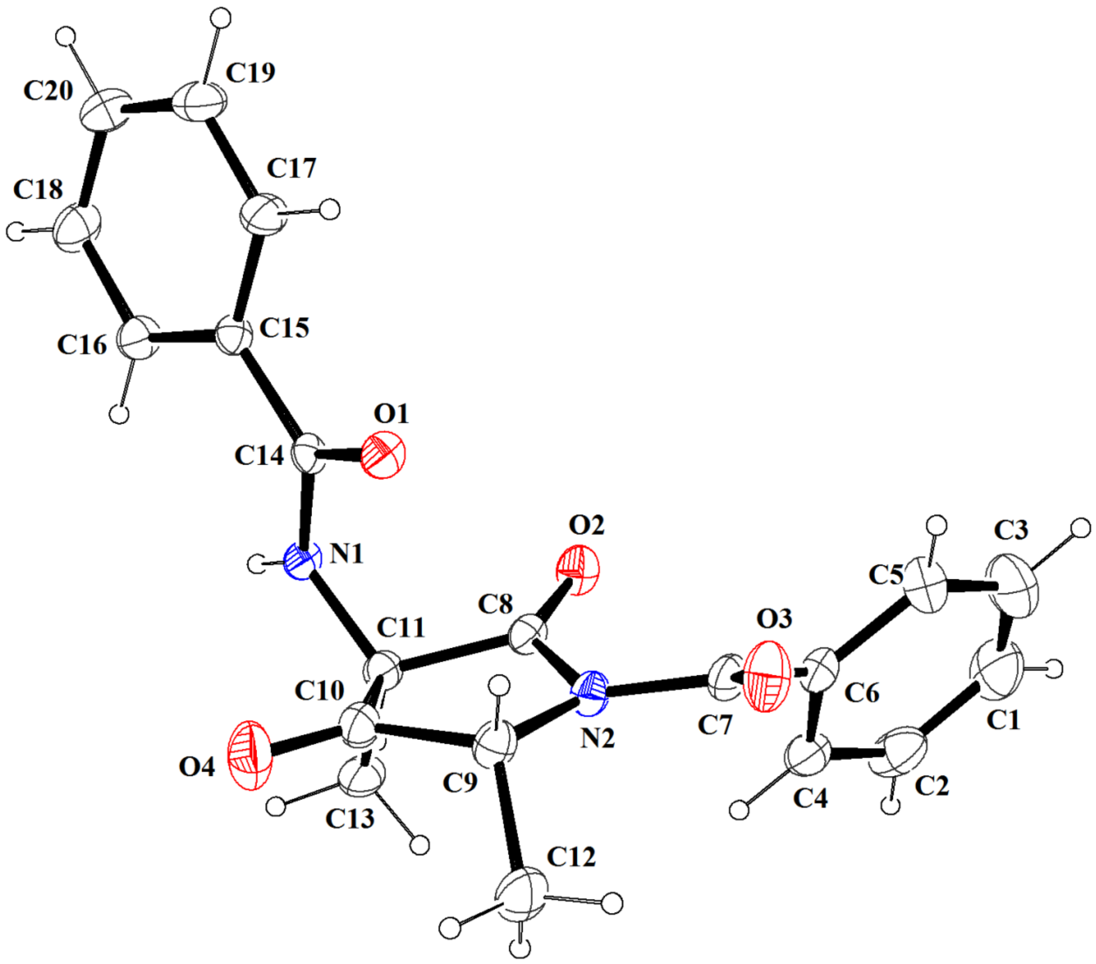

Figure 2. ORTEP representation showing **(a)** the asymmetric unit that contains two independents molecules of **6** and in which one of them have a disordered phenylic ring and **(b)** the independent molecule non-disordered emphasizing the chiral center formed.

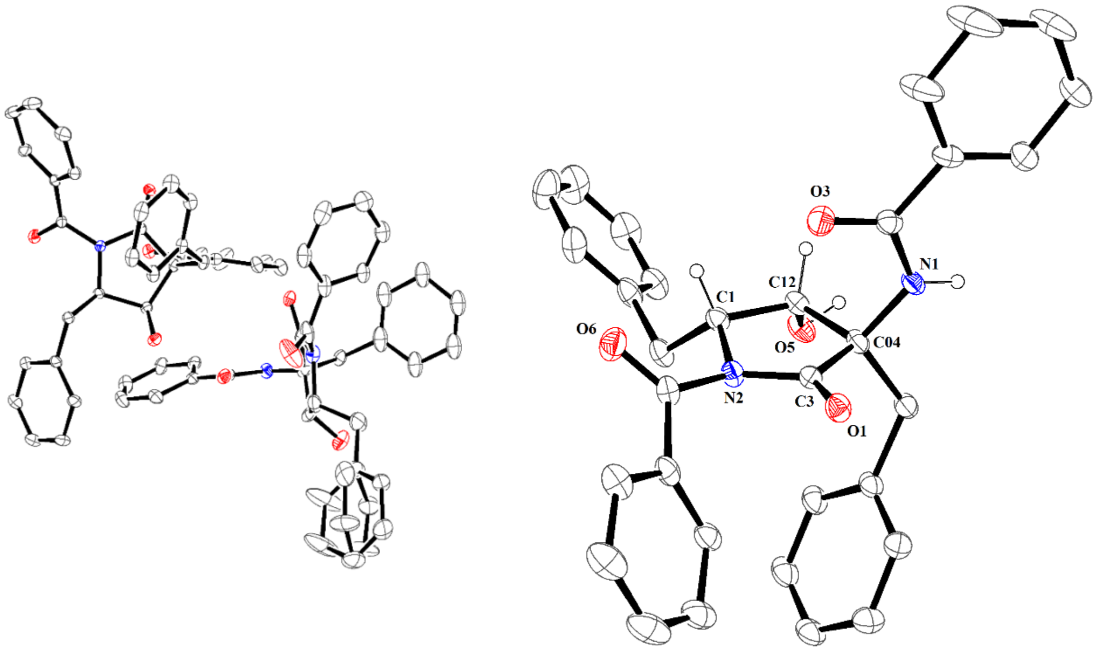

(a) **(b)**

Obs: Some hydrogen atoms had been omitted for more clarification and the anisotropic displacement ellipsoids are drawn at the 30% of probability level.

Table S2. Crystal data, data collection and structure refinement details

**Formula**	$C_{160.66}H_{142.85}N_{16.10}O_{32.23}$ – **2a**	$C_{256}H_{224}N_{16}O_{32}$ **-6**
**Formula weight/g mol^{-1}**	351.47	1009.18
**Temperature/K**	293	150
**Crystal system**	Orthorhombic	Monoclinic
**Space group**	*Pbca*	$P2_1/n$
**a/Å**	16.6696 (5)	14.4834 (7)
**b/Å**	9.8327 (3)	14.1638 (6)
**c/Å**	22.2624 (7)	26.2185 (14)
**β (°)**	90	94.314 (5)
**V/Å^3**	3648.97 (19)	5363.2 (4)
**Z**	8	4
**Radiation type**	Mo *K*α	Cu *K*α
**Crystal size/mm**	0.98 x 0.15 x 0.11	0.33 x 0.12 x 0.08
**d_{calc}/g cm^{-3}**	1.276	1.250
**No. of measured, independent and observed [I > 2σ(I)] reflections**	65705, 5009, 3780	48269, 10392, 8163
R_{int}	0.046	0.065
**Observed reflections**	5009	10392
**N°. of parameters refined**	251	704
$R[F^2>2\sigma(F^2)]$	0.056	0.106
$wR(F^2)$	0.136	0.306
**S**	1.06	1.03
**RMS e. Å^{-3}**	0.033	0.083

Table S3. Selected geometrical parameters in **2a** crystal structure.

**Geometric parameters**			
**Bond distance / Å**			
**O1—C14**	1.2390 (16)	**N1—C14**	1.3382 (18)
**N1—C11**	1.4445 (19)	**O2—C8**	1.1986 (19)
**C14—C15**	1.488 (2)	**N2—C8**	1.3948 (19)
**N2—C7**	1.409 (2)	**N2—C9**	1.476 (2)
**C15—C17**	1.384 (2)	**C15—C16**	1.390 (2)

**C11—C10**	1.523 (2)	**C11—C8**	1.529 (2)
**C11—C13**	1.529 (2)	**O3—C7**	1.210 (2)
**O4—C10**	1.195 (2)	**C7—C6**	1.474 (3)
**C6—C4**	1.380 (3)	**C6—C5**	1.383 (3)
**C16—C18**	1.379 (3)	**C9—C10**	1.517 (2)
**C9—C12**	1.522 (3)	**C17—C19**	1.381 (3)
**C18—C20**	1.365 (3)	**C4—C2**	1.384 (3)
**C20—C19**	1.370 (3)	**C5—C3**	1.370 (3)
**C2—C1**	1.372 (4)	**C3—C1**	1.356 (3)

Bond angles / °

**C14—N1—C11**	117.91 (12)	**O4—C10—C9**	124.86 (17)
**O1—C14—N1**	118.60 (13)	**O4—C10—C11**	124.87 (16)
**O1—C14—C15**	121.48 (13)	**C9—C10—C11**	110.22 (14)
**C8—N2—C7**	124.64 (14)	**C8—N2—C9**	113.67 (12)
**C7—N2—C9**	119.12 (13)	**C17—C15—C16**	118.69 (15)
**C17—C15—C14**	117.80 (13)	**C16—C15—C14**	123.51 (14)
**N1—C11—C10**	112.23 (13)	**N1—C11—C8**	111.61 (12)
**C10—C11—C8**	102.94 (12)	**N1—C11—C13**	110.51 (13)
**C10—C11—C13**	110.16 (15)	**C8—C11—C13**	109.13 (14)
**O2—C8—N2**	126.34 (14)	**O2—C8—C11**	125.54 (14)
**N2—C8—C11**	108.12 (13)	**O3—C7—N2**	119.03 (17)
**O3—C7—C6**	122.38 (16)	**N2—C7—C6**	118.48 (15)
**C4—C6—C5**	119.6 (2)	**C4—C6—C7**	121.41 (17)
**C5—C6—C7**	118.67 (19)	**C18—C16—C15**	120.22 (17)
**N2—C9—C10**	102.69 (13)	**N2—C9—C12**	113.09 (15)
**C10—C9—C12**	112.58 (17)	**C19—C17—C15**	120.28 (17)
**C20—C18—C16**	120.52 (18)	**C6—C4—C2**	119.6 (2)
**C18—C20—C19**	119.84 (18)	**C20—C19—C17**	120.42 (19)
**C3—C5—C6**	120.1 (2)	**C1—C2—C4**	119.7 (3)
**C1—C3—C5**	120.25 (15)	**C3—C1—C2**	120.7 (2)

Torsion angles / °

**C11—N1—C14—O1**	−2.2 (2)	**C8—N2—C9—C10**	8.59 (19)
**C11—N1—C14—C15**	176.35 (13)	**C7—N2—C9—C10**	171.25 (14)
**O1—C14—C15—C17**	12.4 (2)	**C8—N2—C9—C12**	130.17 (18)
**N1—C14—C15—C17**	−166.07 (15)	**C7—N2—C9—C12**	−67.2 (2)
**O1—C14—C15—C16**	−168.00 (16)	**C16—C15—C17—C19**	−1.3 (3)

**N1—C14—C15—C16**	13.5 (2)	**C14—C15—C17—C19**	178.29 (18)
**C14—N1—C11—C10**	56.61 (18)	**N2—C9—C10—O4**	178.97 (19)
**C14—N1—C11—C8**	−58.36 (18)	**C12—C9—C10—O4**	57.0 (3)
**C14—N1—C11—C13**	−179.99 (15)	**N2—C9—C10—C11**	1.73 (19)
**C7—N2—C8—O2**	3.8 (3)	**C12—C9—C10—C11**	−120.19 (18)
**C9—N2—C8—O2**	165.38 (16)	**N1—C11—C10—O4**	52.6 (2)
**C7—N2—C8—C11**	−177.02 (14)	**C8—C11—C10—O4**	172.74 (19)
**C9—N2—C8—C11**	−15.47 (18)	**C13—C11—C10—O4**	−71.0 (2)
**N1—C11—C8—O2**	−45.2 (2)	**N1—C11—C10—C9**	−130.17 (15)
**C10—C11—C8—O2**	−165.78 (16)	**C8—C11—C10—C9**	−10.02 (18)
**C13—C11—C8—O2**	77.2 (2)	**C13—C11—C10—C9**	106.23 (17)
**N1—C11—C8—N2**	135.63 (13)	**C15—C16—C18—C20**	1.3 (3)
**C10—C11—C8—N2**	15.06 (16)	**C5—C6—C4—C2**	0.1 (3)
**C13—C11—C8—N2**	−101.94 (16)	**C7—C6—C4—C2**	−173.32 (18)
**C8—N2—C7—O3**	145.53 (18)	**C16—C18—C20—C19**	−1.6 (3)
**C9—N2—C7—O3**	−15.1 (2)	**C18—C20—C19—C17**	0.5 (4)
**C8—N2—C7—C6**	−38.2 (2)	**C15—C17—C19—C20**	1.0 (3)
**C9—N2—C7—C6**	161.15 (15)	**C4—C6—C5—C3**	1.1 (3)
**O3—C7—C6—C4**	131.9 (2)	**C7—C6—C5—C3**	174.68 (16)
**N2—C7—C6—C4**	−44.2 (2)	**C6—C4—C2—C1**	−0.8 (3)
**O3—C7—C6—C5**	−41.6 (3)	**C6—C5—C3—C1**	−1.6 (3)
**N2—C7—C6—C5**	142.32 (18)	**C5—C3—C1—C2**	0.8 (3)
**C17—C15—C16—C18**	0.2 (3)	**C4—C2—C1—C3**	0.4 (4)
**C14—C15—C16—C18**	−179.39 (16)	**C8—N2—C9—C10**	8.59 (19)
**C11—N1—C14—O1**	−2.2 (2)	**C7—N2—C9—C10**	171.25 (14)
**C11—N1—C14—C15**	176.35 (13)	**C8—N2—C9—C12**	130.17 (18)
**O1—C14—C15—C17**	12.4 (2)	**C7—N2—C9—C12**	−67.2 (2)
**N1—C14—C15—C17**	−166.07 (15)	**C16—C15—C17—C19**	−1.3 (3)
**O1—C14—C15—C16**	−168.00 (16)	**C14—C15—C17—C19**	178.29 (18)
**N1—C14—C15—C16**	13.5 (2)	**N2—C9—C10—O4**	178.97 (19)
**C14—N1—C11—C10**	56.61 (18)	**C12—C9—C10—O4**	57.0 (3)
**C14—N1—C11—C8**	−58.36 (18)	**N2—C9—C10—C11**	1.73 (19)
**C14—N1—C11—C13**	−179.99 (15)	**C12—C9—C10—C11**	−120.19 (18)
**C7—N2—C8—O2**	3.8 (3)	**N1—C11—C10—O4**	52.6 (2)
**C9—N2—C8—O2**	165.38 (16)	**C8—C11—C10—O4**	172.74 (19)
**C7—N2—C8—C11**	−177.02 (14)	**C13—C11—C10—O4**	−71.0 (2)

**C9—N2—C8—C11**	−15.47 (18)	**N1—C11—C10—C9**	−130.17 (15)
**N1—C11—C8—O2**	−45.2 (2)	**C8—C11—C10—C9**	−10.02 (18)
**C10—C11—C8—O2**	−165.78 (16)	**C13—C11—C10—C9**	106.23 (17)
**C13—C11—C8—O2**	77.2 (2)	**C15—C16—C18—C20**	1.3 (3)
**N1—C11—C8—N2**	135.63 (13)	**C5—C6—C4—C2**	0.1 (3)
**C10—C11—C8—N2**	15.06 (16)	**C7—C6—C4—C2**	−173.32 (18)
**C13—C11—C8—N2**	−101.94 (16)	**C16—C18—C20—C19**	−1.6 (3)
**C8—N2—C7—O3**	145.53 (18)	**C18—C20—C19—C17**	0.5 (4)
**C9—N2—C7—O3**	−15.1 (2)	**C15—C17—C19—C20**	1.0 (3)
**C8—N2—C7—C6**	−38.2 (2)	**C4—C6—C5—C3**	1.1 (3)
**C9—N2—C7—C6**	161.15 (15)	**C7—C6—C5—C3**	174.68 (16)
**O3—C7—C6—C4**	131.9 (2)	**C6—C4—C2—C1**	−0.8 (3)
**N2—C7—C6—C4**	−44.2 (2)	**C6—C5—C3—C1**	−1.6 (3)
**O3—C7—C6—C5**	−41.6 (3)	**C5—C3—C1—C2**	0.8 (3)
**N2—C7—C6—C5**	142.32 (18)	**C4—C2—C1—C3**	0.4 (4)
**C17—C15—C16—C18**	0.2 (3)	**C8—N2—C9—C10**	8.59 (19)
**C14—C15—C16—C18**	−179.39 (16)	**C7—N2—C9—C10**	171.25 (14)

Hydrogen bonds

**D—H···A**	**D—H**	**H···A**	**D···A**	**D-H···A**
**C12—H12A···O3**	0.96	2.65	3.157 (3)	113
**N1—H1A···O1^{i}**	0.878 (19)	1.95 (2)	2.8071 (15)	164.3 (16)

Symmetry code: *(i) −x+3/2, y−1/2, z.*

Table S4. Selected geometrical parameters in **6** crystal structure.

Geometric parameters

Bond distance / Å

**O1—C3**	1.226 (4)	**C27—C35**	1.546 (8)
**O2—C5**	1.232 (5)	**O3—C6**	1.231 (4)
**C28—C38**	1.385 (7)	**O4—C30**	1.258 (5)
**C28—C31**	1.396 (7)	**O5—C12**	1.418 (4)
**C29—C41**	1.402 (8)	**N1—C6**	1.362 (4)
**N1—C04**	1.476 (4)	**C32—C51**	1.400 (6)
**O6—C8**	1.214 (5)	**N2—C3**	1.379 (4)
**C33—C39**	1.400 (7)	**N2—C8**	1.430 (4)
**N2—C1**	1.490 (4)	**C34—C43**	1.372 (10)
**N3—C30**	1.347 (5)	**N3—C010**	1.454 (6)
**C36—C41**	1.389 (7)	**C04—C3**	1.533 (5)

**C04—C15**	1.564 (5)	**C04—C12**	1.573 (5)
**O11—C35**	1.434 (5)	**C39—C47**	1.374 (8)
**N6—C5**	1.384 (5)	**N6—C23**	1.420 (7)
**C40—C47**	1.385 (7)	**N6—C27**	1.509 (7)
**C1—C10**	1.539 (4)	**C1—C12**	1.550 (4)
**C42—C54**	1.405 (7)	**C2—C9**	1.393 (6)
**C2—C16**	1.405 (5)	**C45—C55**	1.395 (5)
**C2—C15**	1.530 (5)	**C4—C20**	1.398 (5)
**C46—C10A**	1.545 (8)	**C4—C22**	1.400 (5)
**C4—C8**	1.490 (5)	**C5—C010**	1.549 (6)
**C6—C14**	1.512 (5)	**C48—C55**	1.374 (7)
**C7—C40**	1.401 (6)	**C48—C54**	1.389 (9)
**C7—C18**	1.409 (6)	**C7—C30**	1.494 (6)
**C49—C56**	1.393 (9)	**C9—C31**	1.404 (6)
**C10—C21**	1.524 (5)	**C51—C53**	1.389 (8)
**C52—C59**	1.360 (11)	**C11—C26**	1.395 (6)
**C52—C57**	1.390 (9)	**C11—C34**	1.404 (8)
**C11—C23**	1.488 (8)	**C53—C58**	1.397 (7)
**C010—C37**	1.560 (5)	**C010—C35**	1.568 (6)
**C14—C45**	1.384 (6)	**C56—C59**	1.468 (10)
**C14—C42**	1.396 (6)	**C16—C38**	1.404 (5)
**C10A—C102**	1.325 (13)	**C17—C21**	1.396 (6)
**C10A—C11A**	1.353 (15)	**C17—C58**	1.405 (6)
**C10A—C14A**	1.374 (10)	**C10A—C200**	1.404 (12)
**C18—C33**	1.379 (7)	**C201—C12A**	1.335 (17)
**C201—C15A**	1.346 (15)	**O10—C23**	1.223 (6)
**C201—C300**	1.360 (13)	**C20—C29**	1.390 (6)
**C201—C400**	1.373 (14)	**C21—C32**	1.403 (6)
**C200—C400**	1.384 (14)	**C22—C36**	1.393 (5)
**C24—C50**	1.383 (8)	**C102—C300**	1.377 (13)
**C24—C43**	1.402 (9)	**C25—C57**	1.389 (10)
**C14A—C15A**	1.414 (13)	**C25—C37**	1.521 (8)
**C11A—C12A**	1.446 (16)	**C26—C50**	1.377 (8)
**C27—C46**	1.541 (7)		

Bond angles / °

**C6—N1—C04**	120.8 (3)	**C51—C32—C21**	121.2 (4)

**C3—N2—C8**	123.0 (3)	**C3—N2—C1**	113.7 (3)
**C18—C33—C39**	119.4 (5)	**C8—N2—C1**	117.7 (3)
**C30—N3—C010**	120.7 (4)	**C43—C34—C11**	120.4 (5)
**N1—C04—C3**	110.5 (3)	**N1—C04—C15**	107.2 (3)
**O11—C35—C27**	118.8 (5)	**C3—C04—C15**	109.2 (3)
**O11—C35—C010**	113.1 (4)	**N1—C04—C12**	110.1 (3)
**C27—C35—C010**	106.5 (4)	**C3—C04—C12**	105.0 (2)
**C15—C04—C12**	114.8 (3)	**C5—N6—C23**	122.9 (4)
**C41—C36—C22**	119.6 (4)	**C5—N6—C27**	113.0 (4)
**C23—N6—C27**	119.5 (4)	**N2—C1—C10**	111.4 (3)
**C25—C37—C010**	119.1 (4)	**N2—C1—C12**	104.3 (3)
**C10—C1—C12**	114.6 (3)	**C9—C2—C16**	118.2 (3)
**C28—C38—C16**	120.7 (4)	**C9—C2—C15**	119.8 (3)
**C16—C2—C15**	121.9 (4)	**O1—C3—N2**	125.9 (3)
**C47—C39—C33**	120.6 (5)	**O1—C3—C04**	125.6 (3)
**N2—C3—C04**	108.4 (3)	**C20—C4—C22**	119.6 (3)
**C47—C40—C7**	120.1 (4)	**C20—C4—C8**	117.9 (4)
**C22—C4—C8**	122.1 (3)	**O2—C5—N6**	126.0 (4)
**C36—C41—C29**	120.7 (4)	**O2—C5—C010**	125.4 (3)
**O2—C5—C010**	125.4 (3)	**N6—C5—C010**	108.6 (4)
**O3—C6—N1**	120.8 (3)	**C14—C42—C54**	120.2 (5)
**O3—C6—C14**	121.6 (3)	**N1—C6—C14**	117.5 (3)
**C40—C7—C18**	119.0 (4)	**C34—C43—C24**	120.0 (6)
**C40—C7—C30**	117.5 (4)	**C18—C7—C30**	123.5 (4)
**O6—C8—N2**	119.1 (3)	**C14—C45—C55**	120.3 (4)
**O6—C8—C4**	122.8 (3)	**N2—C8—C4**	117.9 (3)
**C2—C9—C31**	121.5 (4)	**C27—C46—C10A**	115.2 (5)
**C21—C10—C1**	111.4 (3)	**C39—C47—C40**	120.4 (5)
**C26—C11—C34**	118.7 (5)	**C55—C48—C54**	119.8 (4)
**C26—C11—C23**	121.3 (5)	**C34—C11—C23**	119.5 (4)
**O5—C12—C1**	111.9 (3)	**C56—C49—C25**	122.0 (7)
**O5—C12—C04**	114.1 (3)	**C1—C12—C04**	106.1 (3)
**C26—C50—C24**	120.0 (5)	**N3—C010—C5**	110.4 (3)
**C53—C51—C32**	119.9 (4)	**N3—C010—C37**	105.8 (3)
**C5—C010—C37**	109.5 (3)	**N3—C010—C35**	110.0 (4)
**C59—C52—C57**	120.4 (7)	**C5—C010—C35**	105.0 (4)

**C37—C010—C35**	116.1 (3)	**C45—C14—C42**	119.2 (4)
**C51—C53—C58**	119.9 (4)	**C45—C14—C6**	123.4 (3)
**C42—C14—C6**	117.4 (4)	**C2—C15—C04**	117.4 (3)
**C48—C54—C42**	119.7 (5)	**C48—C55—C45**	120.7 (5)
**C38—C16—C2**	120.4 (4)	**C49—C56—C59**	114.9 (8)
**C21—C17—C58**	121.2 (4)	**C25—C57—C52**	119.8 (8)
**C33—C18—C7**	120.5 (4)	**C53—C58—C17**	119.7 (5)
**C29—C20—C4**	120.3 (4)	**C52—C59—C56**	122.9 (6)
**C17—C21—C32**	118.1 (4)	**C11A—C10A—C14A**	116.1 (7)
**C17—C21—C10**	119.6 (3)	**C102—C10A—C200**	120.7 (12)
**C32—C21—C10**	122.3 (4)	**C102—C10A—C46**	120.7 (9)
**C36—C22—C4**	120.3 (4)	**C11A—C10A—C46**	123.0 (6)
**C14A—C10A—C46**	120.3 (6)	**C200—C10A—C46**	118.4 (9)
**O10—C23—N6**	119.0 (5)	**C12A—C201—C15A**	119.7 (9)
**O10—C23—C11**	122.3 (5)	**C300—C201—C400**	112.9 (11)
**N6—C23—C11**	118.6 (4)	**C50—C24—C43**	119.9 (6)
**C400—C200—C10A**	116.5 (14)	**C57—C25—C49**	120.1 (6)
**C57—C25—C37**	121.0 (7)	**C201—C400—C200**	125.1 (14)
**C49—C25—C37**	118.6 (6)	**C50—C26—C11**	120.9 (5)
**C10A—C102—C300**	118.7 (14)	**N6—C27—C46**	111.6 (4)
**N6—C27—C35**	105.0 (4)	**C201—C300—C102**	125.7 (15)
**C46—C27—C35**	111.8 (5)	**C10A—C14A—C15A**	120.2 (9)
**C38—C28—C31**	119.7 (4)	**C10A—C11A—C12A**	122.4 (11)
**C20—C29—C41**	119.5 (4)	**C201—C12A—C11A**	118.4 (12)
**O4—C30—N3**	119.8 (4)	**O4—C30—C7**	122.8 (3)
**C201—C15A—C14A**	121.0 (9)	**N3—C30—C7**	117.4 (4)
**C28—C31—C9**	119.6 (4)		

Torsion angles / °

**C6—N1—C04—C3**	−51.1 (4)	**C5—N6—C27—C35**	10.4 (5)
**C6—N1—C04—C15**	−170.0 (3)	**C23—N6—C27—C35**	167.1 (4)
**C6—N1—C04—C12**	64.5 (4)	**C4—C20—C29—C41**	−0.7 (6)
**C3—N2—C1—C10**	139.3 (3)	**C010—N3—C30—O4**	4.2 (6)
**C8—N2—C1—C10**	−66.1 (4)	**C010—N3—C30—C7**	−176.2 (3)
**C3—N2—C1—C12**	15.2 (3)	**C40—C7—C30—O4**	−21.4 (5)
**C8—N2—C1—C12**	169.8 (3)	**C18—C7—C30—O4**	156.9 (4)

**C8—N2—C3—O1**	12.9 (5)	**C40—C7—C30—N3**	158.9 (4)
**C1—N2—C3—O1**	166.0 (3)	**C18—C7—C30—N3**	−22.8 (5)
**C8—N2—C3—C04**	−169.8 (3)	**C38—C28—C31—C9**	0.8 (6)
**C1—N2—C3—C04**	−16.7 (4)	**C2—C9—C31—C28**	−1.3 (6)
**N1—C04—C3—O1**	−53.2 (4)	**C17—C21—C32—C51**	−0.5 (6)
**C15—C04—C3—O1**	64.4 (4)	**C10—C21—C32—C51**	−179.6 (4)
**C12—C04—C3—O1**	−171.9 (3)	**C7—C18—C33—C39**	−1.8 (7)
**N1—C04—C3—N2**	129.5 (3)	**C26—C11—C34—C43**	−0.2 (7)
**C15—C04—C3—N2**	−112.9 (3)	**C23—C11—C34—C43**	172.3 (4)
**C12—C04—C3—N2**	10.8 (3)	**N6—C27—C35—O11**	127.2 (5)
**C23—N6—C5—O2**	10.5 (6)	**C46—C27—C35—O11**	6.0 (7)
**C27—N6—C5—O2**	166.2 (4)	**N6—C27—C35—C010**	−1.8 (5)
**C23—N6—C5—C010**	−170.5 (4)	**C46—C27—C35—C010**	−123.0 (4)
**C27—N6—C5—C010**	−14.7 (4)	**N3—C010—C35—O11**	102.9 (5)
**C04—N1—C6—O3**	7.7 (5)	**C5—C010—C35—O11**	−138.3 (4)
**C04—N1—C6—C14**	−172.1 (3)	**C37—C010—C35—O11**	−17.2 (7)
**C3—N2—C8—O6**	130.5 (4)	**N3—C010—C35—C27**	−124.9 (4)
**C1—N2—C8—O6**	−21.6 (5)	**C5—C010—C35—C27**	−6.1 (5)
**C3—N2—C8—C4**	−54.6 (4)	**C37—C010—C35—C27**	115.0 (4)
**C1—N2—C8—C4**	153.3 (3)	**C4—C22—C36—C41**	−0.7 (6)
**C20—C4—C8—O6**	−24.5 (5)	**C57—C25—C37—C010**	92.3 (6)
**C22—C4—C8—O6**	148.8 (4)	**C49—C25—C37—C010**	−94.2 (6)
**C20—C4—C8—N2**	160.8 (3)	**N3—C010—C37—C25**	158.4 (5)
**C22—C4—C8—N2**	−25.9 (5)	**C5—C010—C37—C25**	39.4 (6)
**C16—C2—C9—C31**	0.4 (6)	**C35—C010—C37—C25**	−79.2 (6)
**C15—C2—C9—C31**	−178.2 (4)	**C31—C28—C38—C16**	0.6 (6)
**N2—C1—C10—C21**	162.0 (3)	**C2—C16—C38—C28**	−1.5 (6)
**C12—C1—C10—C21**	−79.9 (4)	**C18—C33—C39—C47**	2.3 (8)
**N2—C1—C12—O5**	117.8 (3)	**C18—C7—C40—C47**	0.3 (6)
**C10—C1—C12—O5**	−4.3 (4)	**C30—C7—C40—C47**	178.7 (4)
**N2—C1—C12—C04**	−7.2 (3)	**C22—C36—C41—C29**	−0.1 (6)

**C10—C1—C12—C04**	−129.3 (3)	**C20—C29—C41—C36**	0.9 (7)
**N1—C04—C12—O5**	115.8 (3)	**C45—C14—C42—C54**	−0.9 (8)
**C3—C04—C12—O5**	−125.3 (3)	**C6—C14—C42—C54**	−178.7 (5)
**C15—C04—C12—O5**	−5.3 (4)	**C11—C34—C43—C24**	−1.8 (8)
**N1—C04—C12—C1**	−120.6 (3)	**C50—C24—C43—C34**	2.4 (8)
**C3—C04—C12—C1**	−1.6 (3)	**C42—C14—C45—C55**	0.5 (7)
**C15—C04—C12—C1**	118.4 (3)	**C6—C14—C45—C55**	178.1 (4)
**C30—N3—C010—C5**	−53.1 (5)	**N6—C27—C46—C10A**	150.9 (6)
**C30—N3—C010—C37**	−171.5 (4)	**C35—C27—C46—C10A**	−91.8 (7)
**C30—N3—C010—C35**	62.4 (5)	**C33—C39—C47—C40**	−1.6 (8)
**O2—C5—C010—N3**	−49.8 (5)	**C7—C40—C47—C39**	0.2 (7)
**N6—C5—C010—N3**	131.2 (3)	**C57—C25—C49—C56**	−0.4 (9)
**O2—C5—C010—C37**	66.4 (5)	**C37—C25—C49—C56**	−174.1 (5)
**N6—C5—C010—C37**	−112.7 (4)	**C11—C26—C50—C24**	−1.1 (7)
**O2—C5—C010—C35**	−168.3 (4)	**C43—C24—C50—C26**	−0.9 (8)
**N6—C5—C010—C35**	12.6 (4)	**C21—C32—C51—C53**	1.2 (7)
**O3—C6—C14—C45**	179.2 (4)	**C32—C51—C53—C58**	−0.5 (8)
**N1—C6—C14—C45**	−1.0 (5)	**C55—C48—C54—C42**	2.7 (10)
**O3—C6—C14—C42**	−3.0 (5)	**C14—C42—C54—C48**	−0.7 (10)
**N1—C6—C14—C42**	176.7 (4)	**C54—C48—C55—C45**	−3.1 (9)
**C9—C2—C15—C04**	−105.2 (4)	**C14—C45—C55—C48**	1.6 (7)
**C16—C2—C15—C04**	76.3 (4)	**C25—C49—C56—C59**	−0.7 (8)
**N1—C04—C15—C2**	154.7 (3)	**C49—C25—C57—C52**	1.2 (9)
**C3—C04—C15—C2**	34.9 (4)	**C37—C25—C57—C52**	174.7 (5)
**C12—C04—C15—C2**	−82.7 (4)	**C59—C52—C57—C25**	−0.9 (9)
**C9—C2—C16—C38**	1.0 (5)	**C51—C53—C58—C17**	−0.9 (8)
**C15—C2—C16—C38**	179.5 (3)	**C21—C17—C58—C53**	1.6 (7)
**C40—C7—C18—C33**	0.5 (6)	**C57—C52—C59—C56**	−0.3 (9)
**C30—C7—C18—C33**	−177.8 (4)	**C49—C56—C59—C52**	1.0 (8)
**C22—C4—C20—C29**	−0.1 (5)	**C27—C46—C10A—C102**	−87.7 (11)
**C8—C4—C20—C29**	173.4 (3)	**C27—C46—C10A—C11A**	27.0 (14)

**C58—C17—C21—C32**	−0.9 (6)	**C27—C46—C10A—C14A**	−143.6 (8)
**C1—C10—C21—C17**	−75.9 (4)	**C102—C10A—C200—C400**	6 (2)
**C1—C10—C21—C32**	103.2 (4)	**C46—C10A—C200—C400**	−178.5 (14)
**C20—C4—C22—C36**	0.9 (5)	**C300—C201—C400—C200**	3 (3)
**C8—C4—C22—C36**	−172.4 (3)	**C10A—C200—C400—C201**	−7 (3)
**C5—N6—C23—O10**	133.1 (5)	**C200—C10A—C102—C300**	−1.8 (17)
**C27—N6—C23—O10**	−21.2 (6)	**C46—C10A—C102—C300**	−176.9 (13)
**C5—N6—C23—C11**	−51.5 (6)	**C400—C201—C300—C102**	2 (3)
**C27—N6—C23—C11**	154.3 (4)	**C10A—C102—C300—C201**	−3 (3)
**C26—C11—C23—O10**	140.5 (5)	**C11A—C10A—C14A—C15A**	15.6 (16)
**C34—C11—C23—O10**	−31.9 (7)	**C46—C10A—C14A—C15A**	−173.2 (9)
**C26—C11—C23—N6**	−34.8 (6)	**C14A—C10A—C11A—C12A**	−8.1 (15)
**C34—C11—C23—N6**	152.8 (4)	**C46—C10A—C11A—C12A**	−179.1 (11)
**C34—C11—C26—C50**	1.7 (6)	**C15A—C201—C12A—C11A**	11 (2)
**C23—C11—C26—C50**	−170.7 (4)	**C10A—C11A—C12A—C201**	−5.2 (17)
**C5—N6—C27—C46**	131.8 (5)	**C12A—C201—C15A—C14A**	−4 (2)
**C23—N6—C27—C46**	−71.6 (6)	**C10A—C14A—C15A—C201**	−10.3 (17)

Hydrogen bonds

D—H···A	D—H	H···A	D···A	D-H···A
**N1—H1A···O2**	0.87 (4)	2.23 (4)	3.077 (4)	165 (3)
**N3—H3A···O1^{i}**	0.83 (5)	2.11 (5)	2.933 (4)	169 (4)
**O5—H5O···O4**	0.88 (6)	1.85 (6)	2.719 (4)	172 (5)
**O11—H11···O3^{i}**	0.8200	2.1900	2.911 (5)	148.00
**C10—H10A···O5**	0.9700	2.3700	2.720 (5)	100.00
**C15—H15A···O5**	0.9700	2.4400	2.815 (5)	103.00
**C16—H16···O5**	0.9300	2.4400	3.157 (5)	134.00
**C35—H35···O4**	0.9800	2.4700	2.936 (6)	108.00
**C37—H37A···O11**	0.9700	2.4600	2.848 (6)	104.00
**C42—H42···O3**	0.9300	2.5000	2.808 (5)	100.00
**C45—H45···O2**	0.9300	2.5300	3.410 (5)	159.00
**C46—H46B···O11**	0.9700	2.3900	2.818 (9)	106.00

Symmetry code: *(i) −x+3/2, y+1/2, −z+1/2.*

8- References:

(1) Melhado, A. D.; Amarante, G. W.; Wang, Z. J.; Luparia, M.; Toste, F. D. *J. Am. Chem. Soc.* **2011**, *133*, 3517.

(2) Kobayashi, S.; Bryant, L. L.; Tsukamoto, Y.; Saegusa, T. *Macromolecules* **1986**, *19*, 1547.

(3) Ewenson, A.; Cohen-Suissa, R.; Levian-Teitelbaum, D.; Selinger, Z.; Chorev, M.; Gilon, C. *Int. J. Peptide Protein Res*. **1988**, *31*, 269.

(4) Mazurkiewicz, R.; Pierwocha, A. W.; Fryczkowska, B. J Polish*. Chem.* **1998**, *72*, 113.
